# FORWARDS-1: an adaptive, single-blind, placebo-controlled ascending dose study of acute baclofen on safety parameters in opioid dependence during methadone-maintenance treatment—a pharmacokinetic-pharmacodynamic study

**DOI:** 10.1186/s13063-022-06821-9

**Published:** 2022-10-18

**Authors:** L. M. Paterson, D. Barker, S. Cro, P. Mozgunov, R. Phillips, C. Smith, L. Nahar, S. Paterson, A. R. Lingford-Hughes

**Affiliations:** 1 Division of Psychiatry, Department of Brain Sciences, Imperial College London, London, UK; 2 Imperial Clinical Trials Unit, Imperial College London, London, UK; 3 MRC Biostatistics Unit, University of Cambridge, Cambridge, UK; 4 Toxicology Unit, Imperial College London, London, UK

**Keywords:** Opiate, Addiction, Dependence, GABA-B, Baclofen, Methadone, Phase I, Dose escalation, Adaptive, Bayesian

## Abstract

**Background:**

Treatment of opiate addiction with opiate substitution treatment (e.g. methadone) is beneficial. However, some individuals desire or would benefit from abstinence but there are limited options to attenuate problems with opiate withdrawal. Preclinical and preliminary clinical evidence suggests that the GABA-B agonist, baclofen, has the desired properties to facilitate opiate detoxification and prevent relapse. This study aims to understand whether there are any safety issues in administering baclofen to opioid-dependent individuals receiving methadone.

**Methods:**

Opiate-dependent individuals (DSM-5 severe opioid use disorder) maintained on methadone will be recruited from addiction services in northwest London (NHS and third sector providers). Participants will be medically healthy with no severe chronic obstructive pulmonary disease or type 2 respiratory failure, no current dependence on other substances (excluding nicotine), no current severe DSM-5 psychiatric disorders, and no contraindications for baclofen or 4800 IU vitamin D (placebo). Eligible participants will be randomised in a 3:1 ratio to receive baclofen or placebo in an adaptive, single-blind, ascending dose design. A Bayesian dose-escalation model will inform the baclofen dose (10, 30, 60, or 90 mg) based on the incidence of ‘dose-limiting toxicity’ (DLT) events and participant-specific methadone dose. A range of respiratory, cardiovascular, and sedative measures including the National Early Warning Score (NEWS2) and Glasgow Coma Scale will determine DLT. On the experimental day, participants will consume their usual daily dose of methadone followed by an acute dose of baclofen or placebo (vitamin D3) ~ 1 h later. Measures including oxygen saturation, transcutaneous CO_2_, respiratory rate, QTc interval, subjective effects (sedation, drug liking, craving), plasma levels (baclofen, methadone), and adverse events will be obtained using validated questionnaires and examinations periodically for 5 h after dosing.

**Discussion:**

Study outcomes will determine what dose of baclofen is safe to prescribe to those receiving methadone, to inform a subsequent proof-of-concept trial of the efficacy baclofen to facilitate opiate detoxification. To proceed, the minimum acceptable dose is 30 mg of baclofen in patients receiving ≤ 60 mg/day methadone based on the clinical experience of baclofen’s use in alcoholism and guidelines for the management of opiate dependence.

**Trial registration:**

Clinicaltrials.gov NCT05161351. Registered on 16 December 2021.

**Supplementary Information:**

The online version contains supplementary material available at 10.1186/s13063-022-06821-9.

## Background

Opiate addiction is a major health challenge, and its adverse impact on health and social well-being clearly evident with death rates rising to record levels [1]. It is estimated that opioid-dependent individuals face mortality risks that are 6–20 times higher than the general population; about half of any cohort of opioid users die before they reach 50 years of age [2, 3].

Heroin is the most common illicit drug for which people seek treatment. The harm minimisation approach to treatment with opiate substitution medication (OST) and psychosocial support has been highly effective, but there is now an increasing focus on achieving abstinence [4]. Indeed, abstinence is likely to be better for overall health, particularly in the ageing opiate user population who have been receiving long-term OST and ‘have increasingly complex health and social care needs’ [5]. Chronic opioid exposure is associated with impaired respiratory function, lethal disorders of sleep, cardiovascular disorders, and compromised immune function, particularly when comorbid with HIV [6]. It is also associated with impaired cognitive functioning in domains such as inhibitory control, verbal working memory, cognitive impulsivity, and cognitive flexibility, as well as in decision-making, emotional, and reward processes [7].

Large numbers of individuals receiving OST benefit considerably from their treatment and do not detoxify. However, many desire and would benefit from abstinence but find this hard to achieve and maintain. Opiate withdrawal can be difficult to tolerate due to disturbed sleep, anxiety, and craving. These problems may persist into abstinence, increasing the risk of relapse. Slow tapering of OST can attenuate symptoms during withdrawal. Alternatively, during detoxification, a range of prescribed adjunctive medications may be used to ameliorate symptoms, including hypnotics, sedatives, or α2-adrenoceptor agonists (lofexidine), but their efficacy is limited and/or can only be used short term. Medications for symptomatic relief are associated with tolerance or abuse liability (Z-drugs, benzodiazepines, pregabalin) and significant hypotension (clonidine) or are contraindicated in women of childbearing age (valproate). In the UK, lofexidine was previously prescribed to assist with detoxification but has been unavailable since 2018, exposing an unmet need. With this loss of lofexidine, it is now even more crucial to develop a non-opioid approach to facilitate detoxification. This is particularly relevant when opioid dependence is uncertain since giving opioids can be dangerous (e.g. respiratory depression) or impracticable (e.g. in custody). There is also a need for new treatments because outcomes for successful detoxification are currently poor, with only a minority (30%) of heroin users who enter treatment achieving stable abstinence in 10 to 30 years [8].

Evidence suggests that the GABA-B agonist, baclofen, has the desired properties to facilitate opiate detoxification and prevent relapse in opiate dependence. Baclofen is a generic medication that is currently licenced for spasticity and is well tolerated [9]. Baclofen is currently prescribed off-label to treat alcoholism [10–12], such that rapid expansion and adoption by addiction services would be possible if trial outcomes of safety and efficacy in this indication are favourable. Preclinical evidence shows that baclofen can decrease the self-administration of heroin, antagonise conditioned place preference (CPP) to morphine, reduce stress- and drug-induced reinstatement of opioid CPP, and attenuate morphine withdrawal [13]. There is mechanistic support for baclofen targeting dysregulated neurobiology during opiate withdrawal, like lofexidine [14, 15]. In addition, clinical evidence suggests baclofen may reduce anxiety, muscle aches, insomnia and sleep disturbance, restless legs, and craving [16–25]. Such symptoms contribute to why many find detoxification and early abstinence challenging and support further exploration of the therapeutic potential of baclofen in opiate dependence.

Preliminary data from human lab-based studies and clinical trials of baclofen that include patients on methadone have suggested it may be effective in attenuating opiate withdrawal and in relapse prevention and may thus be safe in combination. For example, baclofen (40–60 mg) was shown to attenuate ‘relatively mild’ opiate withdrawal symptoms by reducing or not taking methadone in a lab-based study [26]. Baclofen (mean dose: 68 mg/day) was also shown to improve opiate withdrawal after abruptly stopping methadone in 2 of 5 participants in an open-label inpatient study [27] and to compare favourably with clonidine in improving ‘mental’ and physical withdrawal in opiate-dependent individuals withdrawing from illicit opiate use (baclofen ≤ 40 mg/day) [28, 29]. Another study investigating cocaine addiction in which 7 of 17 participants were taking methadone [30] found equivocal effects of baclofen (30–60 mg/day) on cocaine-related outcomes but reported no safety issues. In abstinence, a relapse prevention study reported that baclofen (60 mg/day) showed no benefit in opiate-positive urinalysis, but some improvements in treatment retention and symptoms were observed [31]. In all these studies, baclofen was described as well tolerated, with few side effects reported.

To date, there have been no formal RCT studies directly assessing the central nervous system (CNS) depressant effects of oral baclofen in combination with opioids. In related studies, either the dose, route of administration, or duration of baclofen use differs from our proposed indication, or those with substance use disorders are excluded [32–36]. In studies of baclofen alone, toxicity has been reported in individuals with severe renal impairment and was associated with altered consciousness and rarely with respiratory depression [37]. Another study suggested an association between respiratory depression and baclofen use in spasticity with chronic kidney disease, related to increased circulating baclofen levels [38]. Respiratory depression has been described in adolescents consuming between 60 and > 600 mg baclofen in a non-lethal ‘mass intoxication’ [39]. As chronic opioid use, including methadone, is also associated with central sleep apnoeas [40], it is relevant that chronic baclofen has been reported to be associated with central sleep apnoea in four individuals receiving baclofen for alcohol withdrawal (of which *n* = 3 were taking > 150 mg daily) [41]. In contrast, in a study of susceptible snorers, baclofen (25 mg) did not alter sleep-disordered breathing [42].

Whilst no particular safety concerns were raised in any of the studies of baclofen in opioid dependence described above [26–31], the populations studied differed from that within the UK, where individuals tend to be maintained on OST for long periods, and typical community detoxification involves gradual OST reduction over the course of 12 weeks or so. Therefore, it is important to establish that baclofen can be used safely in a typical UK population of opiate-dependent patients undergoing community-based detoxification, particularly from methadone as this is a sedative drug like baclofen. We will then be in a better position to assess its potential efficacy in facilitating opiate detoxification and relapse prevention in a future proof-of-concept efficacy trial.

Therefore, this study aims to understand whether any respiratory, sedative, and cardiovascular effects occur following baclofen administration in combination with methadone. We have adopted a single ascending dose of baclofen protocol incorporating pharmacokinetic-pharmacodynamic (PK-PD) measures to consider the following:

1. The potential for CNS depressant effects of combining baclofen and methadone, in particular, the potential for interaction to cause respiratory depression, marked sedation, and cardiovascular effects.
2. Dose of baclofen: The range of baclofen doses suggested to be effective in alcoholism (30–60 mg/day) is broadly consistent with those administered in the described opiate withdrawal studies (40–80 mg/day). Therefore, we aim for 30 mg as a minimum target dose in our indication, but uncertainty exists around the target maintenance dose.
3. GABA-B receptor sensitivity: We have shown that pharmacodynamic responses to baclofen are markedly blunted in alcoholism suggesting possible alterations in GABA-B receptor function [43]. The same may also apply to opiate dependence [44], with potential consequences for dosing and efficacy signal.
4. The potential for abuse liability: there are particular risks in this vulnerable population of misuse and diversion, and these must be mitigated in any future study.

## Methods

The study methods follow the SPIRIT reporting guidelines [45] and are verified with a completed SPIRIT Checklist. In addition, the estimand framework is incorporated to include handling and reporting of post-randomisation events, in accordance with the ICH E9(R1) clinical trials addendum [46]. Full details can be found in the statistical analysis plan (SAP, see Additional file 1).

### Trial design

The trial will be a single-blind, adaptive, randomised, parallel-group, placebo-controlled ascending dose study of a single dose of baclofen in opiate-dependent individuals stably maintained on methadone. The study will evaluate the safety of combining baclofen and methadone by investigating the impact on respiratory, cardiovascular and PK-PD parameters. Participants will be randomised in a 3:1 ratio to baclofen or placebo. Participants allocated to baclofen will be dosed (10, 30, 60, or 90 mg) in groups of up to 3, with a maximum available sample size of 64 (up to 48 on baclofen and 16 on placebo). An adaptive model (described below) will inform the dosage of baclofen for each patient group based on the trial data accumulated to date and the participant’s methadone dose. The dose setting committee (DSC) will retain the ability to override the model’s dose recommendation if clinically indicated. Data from this study will inform us what dose of baclofen is safe to prescribe to those receiving methadone in a subsequent proof-of-concept trial of the efficacy of baclofen to facilitate opiate detoxification. Based on the clinical experience of using baclofen in alcoholism and guidelines for the management of opiate dependence, we have selected the minimum acceptable combination doses of baclofen and methadone as 30 mg and < 60 mg/day, respectively [10, 47–49]. We will recruit individuals prescribed a range of methadone doses up to 120 mg/day and will assess doses of up to 90 mg baclofen to provide the full range of prescribing freedom, if required, in the subsequent proof-of-concept trial.

### Primary study objective

To establish whether we can safely proceed with prescribing a minimum of 30 mg baclofen to patients receiving a range of doses of methadone. Safety will be determined through the incidence of dose-limiting toxicity (DLT) events relating to CNS depressant activity including the requirement for intervention, respiratory function, cardiovascular function, and sedation.

### Secondary study objectives

Secondary objectives will include the determination of evidence of [i] sub-threshold dose-limiting toxicity events relating to respiratory, cardiovascular, or sedation changes in response to baclofen; [ii] abuse liability signal for baclofen; and [iii] reduced sensitivity to baclofen using self-reported subjective drug response. We will investigate the impact of the different baclofen levels on the variability of response in the aforementioned measures. We will also determine the impact of methadone dose level and gender.

### Exploratory objectives

Exploratory objectives include the characterisation of GABA-B receptor sensitivity in this population using pharmacokinetic-pharmacodynamic (PK-PD) endpoints at the different baclofen dose levels, relative to placebo as well as the impact of baclofen on sleep. Full details of exploratory objectives, outcomes, and hypotheses are included in Additional file 2, alongside planned analyses. Exploratory objectives will not be addressed as part of the main trial analysis and will be reported at a later date.

### Hypotheses

We anticipate no evidence of clinically significant respiratory depression or cardiovascular changes from doses up to 90 mg of baclofen in those on daily methadone doses ≤ 120 mg/day.

We hypothesise that we will observe no indication of abuse liability of baclofen relative to placebo in combination with methadone.

We hypothesise that we will observe increased self-reported measures of drug effect, including sedation, with doses at or above 60 mg baclofen in combination with methadone doses at or above 60 mg, at peak effect (2–3 h following dosing), relative to placebo.

### Primary outcome

The primary outcome is the maximum safe dose(s) of baclofen corresponding to the risk of a dose-limiting toxicity (DLT) of 15–25% in evaluable participants for prescribed doses of methadone.

Determination of whether a DLT event has occurred is made according to the incidence of *one or more* of the following outcomes:

1. Situation requiring intervention level ≥ 4 at any time, scored according to step-wise algorithm: If marked sedation or apnoea > 30 s occurs, the required level of stimulus intervention to rouse will be scored: 0, no intervention; 1, indirect noise, e.g. door opening, closure, cough; 2, interrupt patient with direct speech; 3, touch; and 4, unable to rouse patient with touch
2. National Early Warning Scale (NEWS2) score > 4 or a score of 3 in any parameter (the thresholds for triggering an ‘urgent ward-based response’)
3. Measures of respiration with a persistent change in at least one of (a) reduction in SpO_2_ [≤ 91% for more than 30 seconds or > 5% reduction in SpO_2_ for more than 30 s], (b) reduced respiratory rate (≤ 8/min), and (c) absence of inspiratory airflow for > 30 s combined with a sustained fall in SpO_2_
4. Glasgow Coma Scale (GCS) score < 12
5. Persistent electrocardiogram (ECG) QTc prolongation (> 500 ms or increase of > 60 ms)

### Secondary outcomes

Secondary outcomes include the individual components of the DLT definition and sub-threshold DLT definition (described below) for which frequencies will be separately reported for each intervention, by baclofen dose group and gender. We will describe the time course of SpO_2_, CO_2_, and respiratory rate following baclofen dosing, relative to placebo. We will also assess sedation as measured by the mean T-SHAS score (total score on Subjective High Assessment Scale [50] and symptoms as measured by the Drug Effects Questionnaire (DEQ, [51], mean ‘drug liking’, and ‘want more score’. Secondary outcomes will be assessed at each time point by intervention group.

A sub-threshold DLT is defined as:

1. SpO_2_—instances of < 92% or of > 5% reduction for > 10 s
2. CO_2_—instances of ETCO_2_% per breath exceeding 6.5% [52] or a partial pressure CO_2_ increase by 1kPa
3. Respiratory rate—instances of absence of inspiratory airflow for more than 10 s or respiratory rate drops < 9/min

### Rationale for outcome selection

#### Respiratory function, sedation, and cardiovascular effects

Standard clinical practice for measuring respiratory depression involves monitoring for hypoventilation using pulse oximetry and respiratory rate. Measuring carbon dioxide partial pressure (pCO_2_) provides a more sensitive and earlier indicator of respiratory depression. In addition, evidence suggests that individuals with opiate dependence may display hyposensitivity to pCO_2_ [52, 53]. Whilst there is no unified definition of ‘respiratory depression’, indicators of significant respiratory depression are as follows: persistent reductions in SpO_2_ (e.g. < 90% for more than 10 s [52]), absence of inspiratory airflow (apnoea) > 30 s combined with a sustained fall in SpO_2_, sustained ETCO_2_% per breath exceeding 6.5% [52], or sustained CO_2_ partial pressure increase by 1 kPa (normal range 4.7–6.0 kPa) and respiratory rate ≤ 8/min (from the National Early Warning Score (NEWS2) which is widely used in the UK for assessment and response to acute illness [54].

Sedation is the most common side effect of baclofen, particularly at higher doses. In healthy controls, we observed mild to moderate sedation following acute doses of 60 mg and above, as measured by the self-reported drug effects Subjective High Assessment Scale (SHAS) scale [50] but no such sedation in alcohol-dependent participants with doses up to 90 mg [43]. We will monitor sedation using the Glasgow Coma Scale (GCS), SHAS scale, and the self-reported Leeds Sleep Evaluation Questionnaire (LSEQ [55], which includes specific items related to sedation.

Cardiovascular effects are not commonly reported in response to baclofen, except in studies of renal impairment or after very high doses, e.g. in overdose [56, 57]. A small increase in heart rate (10 bpm) and blood pressure (121 to 125 mmHg) at 2 h post-dose of 80 mg baclofen was seen which returned to normal after 6 h [58]. In our acute study, we observed no overall significant effects on heart rate or blood pressure [43]. We will monitor cardiovascular parameters and in particular QT interval given methadone’s potential for QT prolongation, though there are no reports of such an effect with baclofen.

#### Abuse liability

Concerns have been raised about the potential for abuse liability of baclofen [59, 60] following clinical reports of ‘liking’ in alcohol dependent populations, though no evidence was reported in randomised controlled trials of baclofen in alcohol dependence. Possible evidence of abuse liability of baclofen in alcohol dependence was observed when used in combination with alcohol, but not baclofen alone [61]. Baclofen (80 mg) in heavy social drinkers resulted in no change in ‘liking’, but ‘good drug effect’ and ‘elevated mood’ were reported [58]. Evidence from our study in alcohol dependence reported no significant ‘alcohol-like’ or ‘drunk’ effects on the subjective high assessment scale (SHAS) or drug effects questionnaire (DEQ [51], following one dose of 60 or 90 mg of baclofen, though there was a suggestion of increased ‘high’ and ‘liking’ effects on DEQ after the 90 mg dose [43]; therefore, we will use the DEQ to assess abuse liability (secondary objective).

#### Dosing considerations and GABA-B sensitivity

In prescribing baclofen for alcohol dependence, variability in response and the lack of robust biomarkers to inform the maintenance dose means that the target dose for an individual is not well characterised. Several trials have shown baclofen to be efficacious in relapse prevention at 30 mg daily taken in 3 divided doses [62–64] though higher doses (≤ 300 mg/day) have also been used, and meta-analyses have established that doses ≥ 60 mg are no more effective than lower doses and likely incur more adverse effects in alcoholism [10–12]. Safety concerns led the French authorities to approve use of baclofen in alcoholism only up to 80 mg/day [65]. Based on this evidence, in this trial, our minimum target safe dose of baclofen is 30 mg, with doses of 60 and 90 mg assessed in accordance with our study of alcohol dependence.

To further inform any decisions about what dose of baclofen is optimal, plasma levels of baclofen and methadone will be assessed alongside plasma growth hormone (GH) levels (ng/ml, see the ‘Exploratory objectives’). The latter reflects GABA-B sensitivity to baclofen, and the measures complement our pharmacokinetic-pharmacodynamic (PK-PD) study in alcoholism that showed blunted GH response to baclofen with no difference in plasma baclofen levels compared with controls [43]. This suggests the GABA-B system may be less sensitive in alcoholism and therefore possibly in opiate addiction, as previously suggested [44].

### Primary estimand

The primary estimand for the primary objective is described by the following attributes:

1. Population: Opiate-dependent individuals stably maintained on methadone meeting the inclusion/exclusion criteria who are able to receive baclofen.
2. Treatment condition: baclofen at the dose received. The allocation dose is specific to the entry cohort as recommended by the Dose Setting Committee (DSC), based upon the dose-combination toxicity model.
3. Outcome: Incidence of dose-limiting toxicity (DLT).
4. Handling of intercurrent events:
  - Treatment not received—intention to use the principal stratum strategy and target incidence of DLT only within the principal stratum (i.e. subset) of patients who would receive study treatment (baclofen at any dose). Thus, only patients who receive treatment will be included in the analysis.
  - Dose received not as recommended—intention to target incidence of DLT for received doses (see treatment condition). Thus, if a dose is not received as recommended, patients will be analysed using the actual dose received. The dose-combination toxicity model is flexible and can include continuous doses of baclofen.
5. Population-level summary measure: The target safe baclofen dose from the dose-combination toxicity model which may or may not vary by methadone dose; if this varies by methadone dose, the target safe baclofen doses will be accompanied by associated methadone dose ranges. Supporting population-level summary measures are (i) mean probability of DLT with accompanying 95% Credible Intervals, (ii) probability of DLT being in the target range of 15–25%, and (iii) probability of DLT being above the target range of 25%, assessed for 60 mg of methadone in combination with 30 mg of baclofen and for 120 mg of methadone in combination with 90 mg of baclofen from the dose-combination toxicity model. The frequency and percentage of patients experiencing a DLT, and the DLT parameters they occurred in, over all doses of baclofen, by baclofen dose cohort (as received) and by gender (across all methadone doses) are also of interest.

### Participants

All participants (male or female; > 21 years) will have met the DSM-5 diagnosis of severe opioid use disorder and be currently treated with and able to maintain the same dose of methadone (≤ 120 mg/day) during the study. They will be medically healthy and able to receive baclofen (≤ 90 mg) or 4800 IU vitamin D (placebo). They will be able to read, comprehend and record information written in English. The exclusion criteria include current DSM-5 substance dependence disorder (except opiates, nicotine), current or past severe DSM-5 psychiatric disorder, active suicidality, significant head injury, pregnancy or breast-feeding, severe chronic obstructive pulmonary disease or type 2 respiratory failure, pulse rate < 40 or > 100 BPM, systolic blood pressure > 160 and < 100 and a diastolic blood pressure > 95 and < 60 in the semi-supine position, oxygen saturation < 92% at rest, QTcB or QTcF > 500 ms, regular on-top use of heroin or other substances, or use of any medications which in the opinion of the investigators will interfere with subject safety or study integrity. Intoxication or positive drug/alcohol screen on a study day would preclude them for that day but not the whole study. A lifetime history of other substance use disorders and current moderate or mild DSM-5 depressive, anxiety, sleep, or personality disorders are acceptable given high levels of comorbidity.

### Recruitment

Opiate-dependent individuals will be recruited from community-based addiction services within the UK including those in the NHS, e.g. Central North West London NHS Foundation Trust (CNWL) and its partners, and the third sector, e.g. Change Grow Live (CGL) based in northwest London, UK, and surrounding areas. They will be recruited by the directed advertisement at those services, via referral, or via an investigator-led approach at NHS trusts, voluntary sector or partner organisations through participant identification centres (PIC) or equivalent, subsequent to relevant approvals. To achieve adequate recruitment, we will include more PICs that are suitable for participants to travel for the study. Increasing visibility of the study and additional recruitment channels may also involve widespread advertisement in social media, dedicated study Facebook pages or social media presence, and advertising on relevant websites related to addiction.

### Study schedule overview

Potentially eligible participants will undergo telephone pre-screening and be invited to attend an in-person screening visit at one of the study sites. After the in-person screening, eligible participants will be enrolled into the study, randomised, and invited to attend the experimental visit, which will consist of a single experimental visit plus next-day telephone follow-up (Table 1). Participants will receive compensation for taking part; £50 for screening and £100 for a completed experimental visit, either by bank transfer or vouchers (as requested). As all participants will continue to receive their usual treatment for their opiate dependence during the study, there will be no-post trial care from the research team.

**Table 1:** Schedule of events

Event	Screening	Enrolment	Experimental	Follow-up
General				
Consent and eligibility	x		x	
General health	x		x	x
Demographics	x			
Clinical assessments				
Structured clinical interview (MINI)	x			
Medical examination	x			
Vital statistics (height, weight)	x			
Vital signs (BP, HR, SpO_2_)	x		x	
Respiratory function	x		x	
Blood sampling (clinical)	(x)			
Urine screen (DOA and pregnancy)	x		x	
Breath alcohol	x		x	
Methadone administration			x	
Baclofen or Placebo administration			x	
Drug and alcohol history				
Methadone dose check	x		x	
TLFB (drug and alcohol use)	x			
FTND	x			
AUDIT	x			
ASSIST (shortened)	x			
SDS	x			
OCDUS-H	x			
Randomisation				
Randomisation and enrolment		x		
Mood, sleep, and personality				
BDI	x			
STAI	x			
PSQI	x			
RLS Scale	x			
STOP-BANG	x			
ESS	x			
State measures				
VAS drug effects (SHAS, DEQ)			x	
VAS craving, anxiety	x		x	
COWS	x		x	
LSEQ, sleep quality			x	x
Adverse events			x	x
Blood measures				
Growth hormone			x	
Baclofen/methadone			x	
Actigraphy measures				
Actigraphy/SpO_2_ finger monitor	x	(x)	x	x

### Schedule of events

#### Screening and participant characterisation

Informed consent will be obtained by one of the qualified research team. Following this, study eligibility will be ascertained and medical, psychiatric, and dependence history and current status obtained using the Modified Mini International Neuropsychiatric Interview (MINI) [66]; Fagerstrom test for nicotine dependence (FTND) [67]; Alcohol Use Disorders Identification Test (AUDIT) for current alcohol use [68]; Alcohol, Smoking, Substance Involvement & Screening Test (ASSIST) for illicit drug use [69]; Severity of Dependence Scale (SDS) [70]; Obsessive-Compulsive Drug Use Scale (OCDUS-H) [71]; Beck Depression Inventory (BDI-II) [72]; Spielberger Trait Anxiety Scale (STAI) [73]; Pittsburgh Sleep Quality Index (PSQI) [74]; Epworth Sleepiness Scale (ESS) [75]; STOP-BANG questionnaire for sleep disordered breathing [76]; Visual Analogue Scales (VAS) of craving and anxiety; Clinical Opiate Withdrawal Scale (COWS) [77]; and Restless Legs Severity scale (RLS scale) [78] (see Table 1). Participants will be given an actiwatch, a non-invasive wrist-worn device that records sleep-wake activity, to be worn until the day following the experimental visit.

#### Randomisation and blinding

Eligible participants will be enrolled into the study by a member of the research team and randomised to receive a single, oral, acute dose of baclofen or placebo (3:1 ratio respectively) as directed by the randomisation schedule. The concealed randomisation allocation sequence will be generated and maintained by the online software application Sealed Envelope [79]. Blocked randomisation will be used to maintain the 3:1 ratio throughout the study. Participants will remain blinded throughout the study, whilst study clinicians and researchers will be aware of the allocation once randomisation has occurred (single-blind). Participants will not be told that the placebo tablets contain vitamin D as this could also create expectation effects. Instead, they will be told that these are ‘dummy’ pills, and we will check for vitamin D contraindications at screening.

#### Intervention

Participants allocated to receive baclofen will receive an acute dose of either 10, 30, 60, or 90 mg, and those allocated placebo will receive an equivalent number of vitamin D tablets (up to a maximum of 120 micrograms or 4800 IU). The baclofen dose will be recommended by the Bayesian dose-escalation adaptive model (described below), in which dose allocation will be informed by the accumulating occurrences of dose-limiting toxicity (DLT) events at increasing doses of baclofen, along with the corresponding participant doses of prescribed methadone [80]. Baclofen (generic) will be supplied as white, round, scored 10 mg tablets in the required dose. Placebo tablets will be vitamin D3 (colecalciferol; 20 μg/800 IU, white, round, scored tablets) that are a near identical match to baclofen. Both baclofen and vitamin D3 will be supplied by Imperial College Healthcare NHS Trust pharmacy (Hammersmith Hospital).

#### Experimental day

Following randomisation, participants will attend the Imperial Clinical Research Facility (ICRF) for a single experimental visit. They will consume their usual daily dose of methadone under observation soon after arrival, followed by an acute oral dose of baclofen or placebo approximately 1 h later. Measures of respiratory, cardiovascular, and subjective effects and adverse events will be obtained periodically for up to 5 h after baclofen dose, according to Table 2. Blood samples will be obtained where possible for assessment of growth hormone, baclofen and methadone levels. Samples will be stored until the analysis is complete. Any remaining samples will either be destroyed or stored under the relevant UK Human Tissue Authority (HTA) licence, in accordance with participant consent. Participants will be given a finger-worn oximetry device that records oxygen saturation, to be worn until the day following the experimental visit.

**Table 2:** Schedule of events during experimental day

***Procedure***	Time point (min post-baclofen/placebo)								
	^**a**^Baseline	15	30	60	90	120	180	240	300
*NEWS2*	x	–	–	x	–	x	x	x	x
*SpO*_*2*_	x	x^b^	x^b^	x	x^b^	x	x	x	x
*RR*	x	x^b^	x^b^	x	x^b^	x	x	x	x
*BP*	x	–	–	x	–	x	x	x	x
*HR*	x	–	–	x	–	x	x	x	x
*Body temperature*	x	–	–	x	–	x	x	x	x
*LOC*	x	–	–	x	–	x	x	x	x
*GCS*	x	–	–	x	–	x	x	x	x
*ECG*	x	–	–	–	–	x	–	–	x
*tcCO*_*2*_	x	x	x	x	x	x	x	x	x
*SHAS*	x	–	–	x	–	x	x	x	x
*DEQ*	x	–	–	x	–	x	x	x	x
*VAS craving/anxiety*	x	–	–	x	–	x	x	x	x
*Blood plasma/serum*	x	–	–	x	–	x	x	–	x
*COWS*	x	–	–	–	–	x	–	–	x

*RR* respiratory rate, *BP* blood pressure, *HR* heart rate, *LOC* level of consciousness

^a^Baseline refers to the measurements obtained prior to time 0, which represents the time at which baclofen/placebo is administered. Shaded areas are measures comprising the NEWS2 score

^b^At these time points, SpO_2_ and RR measures will be taken in addition to those associated with the NEWS2

#### Follow-up

The following day, participants will receive a follow-up phone call to check on their welfare, if they have any concerns, overnight sleep quality (LSEQ) and for any adverse events. They will be reminded to return their actiwatch and oximetry device via pre-paid envelope, or to their local addiction service, as appropriate.

### Evaluation window

The evaluation window for primary and secondary outcome measures will begin at dosing and end at 5 h post-dose, with the exception of the ‘intervention level’ (see DLT definition) which will begin at dosing and continue until the last follow-up phone call. This call will be conducted the following day, and the window will be extended if the participant is experiencing sedation or other symptoms.

### Evaluable patient

An evaluable participant is defined as one who has received study medication and has provided sufficient data to meet the primary endpoint of determining the presence or absence of a DLT and sufficient data relating to the main secondary outcome measures. Whether sufficient data has been obtained for this purpose will be determined by clinical judgement on an individual participant basis.

In the unlikely event that a participant decided to self-discharge themselves after dosing but prior to the end of the 5 h evaluation window, this participant could be counted as an evaluable patient with no DLTs provided they had completed the 2–3 h time point and that this self-discharge had occurred within a clinical picture of stable or normalising observations. Attempts to complete the follow-up phone call would be made to confirm the absence of DLT as defined by ‘intervention level’.

In the event of an inability to acquire sufficient data, as defined above, the decision on whether to include an individual’s data in the primary (DLT) or secondary analysis, or whether that individual would be replaced, would be made on a case-by-case basis.

#### Sample size

The sample size is not fixed. Up to a total of 64 patients are planned to be enrolled in the study with up to 48 patients on the experimental baclofen doses and 16 on placebo with a 3:1 allocation ratio. The final sample size will depend on the escalation/de-escalation decisions made during the trial and on the recommendation of the model on stopping earlier. The performance of the Bayesian design based on 48 participants was assessed via simulations under several clinically relevant scenarios in terms of accuracy of the number of patients that would receive (i) their individual target dose combination and (ii) a combination that is safe for them in the subsequently planned proof-of-concept efficacy trial. Simulations confirmed desirable operating characteristics with a sample size of 48; the average proportion of patients allocated to target doses was 62–93%, and the average proportion of patients allocated to safe doses was 74–99%. Under an unsafe scenario, the design terminates the trial with a probability of 90% [80].

### Statistics and data analysis plan

#### Treatment algorithm to determine the dose of baclofen

Participants allocated to baclofen will be dosed in groups of up to 3, with a maximum available sample size of 64 (up to 48 on baclofen and 16 on placebo). The doses of baclofen to be potentially explored in the trial are 10, 30, 60, and 90 mg. The lowest dose of baclofen (10 mg) will be allocated to the first group of 3 participants assigned to receive baclofen. A Bayesian dose-escalation adaptive model will inform the dosage of baclofen for each subsequent participant group based on the incidence of ‘dose-limiting toxicity’ (DLT, see above for definition) and participant-specific methadone dose. The model is a 5-parameter Bayesian logistic regression model with an interaction parameter and has been described in detail elsewhere [80]. A DLT rate of 20% with an equivalence interval of 15–25% will be targeted in order to estimate the current maximum tolerated dose (MTD), conditional on the participant’s methadone dose, in dose escalation. The model will be updated after each group of at most 3 participants on baclofen, according to the observed participants’ responses. Dose escalation constraints include (1) doses of baclofen to be tested cannot be skipped, and (2) if any of the current cohort experience a DLT, then the next group of participants will be assigned to the current (or lower) dose of baclofen at which the DLT was experienced, without dose escalation, depending on the prescribed dose of methadone for the next participants that enter the study. If there is no DLT, a dose escalation may be advised by the model, again, depending on the prescribed dose of methadone for the next participant that enters the study. The model may recommend to stop the trial earlier due to safety concerns if the probability that the risk of DLT for 60 mg of methadone in combination with 30 mg of baclofen exceeds the target range (15–25%) is 25% or more. The model may recommend to stop the trial earlier due to all doses of baclofen being safe if the probability that the risk of DLT for 120 mg of methadone in combination with 90 mg of baclofen does not exceed the target range (15–25%) is 92.5% or more, representing the ‘all-safe-stop criteria’. The ‘dose setting committee’ (DSC) comprised at least (1) the PI (ALH) or nominated delegated clinician, (2) the PI (ALH) or study manager (LP), and (3) a named trial statistician (SC, RP, PM). The DSC will review all dose recommendations from the model and retains the ability to override the algorithm’s dose recommendation if clinically indicated.

In the scenario that there is a very high probability that all doses are safe (90 mg baclofen with up to and including 120 mg methadone) before reaching the maximum recruitment of *n* = 48 receiving baclofen, the study will retain the ability to override the all-safe-stop criteria to continue recruitment until we have tested a sufficient range of doses of methadone to ensure we have covered the clinical range of potential methadone doses and/or to explore baclofen dose separation to achieve maximum precision on secondary outcomes.

#### Data analysis plan

No formal statistical testing will be conducted for this early phase study, and no power calculation has been performed; the analyses will primarily be descriptive except for possible exploratory analyses as deemed appropriate. All statistical analyses for primary and secondary outcomes are to be viewed as exploratory. The full analysis set includes all randomised participants and will be used to summarise study conduct and participant disposition. The safety analysis set will consist of all participants who are randomised and received the study drug. Participants in this analysis set will be used for demographic, baseline characteristics, and safety summaries. The DLT evaluable safety analysis set will consist of all participants who are randomised and received the study drug and are evaluable for a DLT (described above). Participants in this analysis set will be used for the primary dose-combination toxicity response analysis (included in the Bayesian analysis model). Participants will be grouped according to the actual treatment received. Using the final model update the mean estimate of the DLT probability for each baclofen dose with accompanying 95% credible intervals, the probability of DLT being in the target range of 15–25% and the probability of DLT being above the target range of 25% for 60 mg of methadone in combination with 30 mg of baclofen and the probability of DLT not exceeding the target range for 120 mg of methadone in combination with 90 mg of baclofen will be presented. The analysis set for each secondary outcome will be based on the subset of patients from the safety set for whom at least one measurable outcome has been obtained. Descriptive statistics (means/medians, with standard deviations/inter-quartile range (SD/IQR) or frequencies and percentages as appropriate for the data distribution) will be presented for each outcome by time point and treatment group. Outcomes will also be presented graphically over time and treatment group using appropriate summary statistics for the data distribution. Descriptive statistics will be also presented for secondary outcomes by baclofen dose and gender. Correlation analyses, using Pearson or Spearman’s rank correlation coefficient, will be conducted to explore the associations between outcomes and methadone dose. For repeated continuous measures linear mixed models may be used to explore the effects of time and group (placebo versus baclofen) if adequate data collection allows. Where data further allows, linear mixed models may be extended to explore the effects of baclofen dose and methadone dose on secondary outcomes. Analysis of PK-PD endpoints to determine GABA-B sensitivity will be analysed as previously described [41]. An assessment of sources of variability, e.g. age, dose on PK-PD, and safety outcomes, will be made. Analyses will be conducted using the SPSS, Stata, or R software and the statistical analysis plan (SAP) describes the trial estimands and analyses in more detail (see Additional file 1).

#### Adverse events

Adverse events will be tabulated by type (e.g. adverse event, adverse reaction, unexpected adverse reaction, serious adverse event, serious adverse reaction, unexpected serious adverse reaction) and treatment group (baclofen versus placebo) for the safety analysis set. Adverse events coded as per the MedDra dictionary will be summarised at the preferred term level and body system class and include information on the number with at least one event and the number of events. Summaries by treatment arm and severity grade will include information on the number with at least one event by maximum grade and the number of events. Risk differences between those experiencing at least one event for each event between treatment group with corresponding 95% confidence intervals (CIs) will be calculated and incident rate ratios to account for recurrent events with 95% CIs using a suitable model, e.g. a negative binomial or zero-inflated Poisson model as appropriate. These results will be presented in both a table and graphically (see Additional file 1 for more detail).

#### Missing data

Regarding missing data, every effort will be made to obtain all follow-up data for all participants. We anticipate minimal missing data as the evaluation window for primary and secondary outcome measures will begin at dosing and end at 5 h post-dose, with the exception of the ‘intervention level’ (as defined in the DLT definition) which will begin at dosing and continue until the last follow-up phone call (at 1 day). Across primary and secondary analyses data summarises will be taken from observed data only. In the rare case of missing data on the primary outcome (DLT) for any participant who withdraws after dosing, but before adequate data is obtained, sensitivity analysis will be conducted for the primary analysis to explore the robustness of alternative missing data assumptions as follows: (i) participants who receive treatment with missing data included in the sensitivity analysis and assumed to have experienced a DLT and (ii) participants who receive treatment with missing data included in the sensitivity analysis and assumed to not have experienced a DLT. Linear mixed model analyses employ maximum likelihood estimation and thus are efficient for handing missing outcome data under a missing at random (MAR) assumption.

### Data collection and management

This study and its staff will be compliant with the Data Protection Act and General Data Protection Regulation with regard to the collection, storage, processing, and disclosure of personal information. Each participant will be identified by a unique code number that will be used throughout the duration of the study*.* Participant names, addresses, and other contact details will be written in the clinical screening portion of the paper-based case report form (CRF) for identification and contact purposes. The clinical screening CRFs will be regarded as confidential, and kept in locked filing cabinets in Imperial College. The contact details will then be removed from the CRF and into participant notes for storage. Only the PI, and selected study team members will have access to anonymised codes and their link to personal ID. This will be kept locked in a file on site and electronically on secure servers accessed by research team members only (password protected).

All data will be collected in a pseudonymised and coded manner and stored within paper-based CRFs and/or via electronic data capture within purpose-built secure-access online databases; OpenClinica for primary and secondary outcomes, REDCap for exploratory outcomes [81, 82], saved electronically on secure University (Imperial College) computer systems and facilities. Primary outcome source data (DLT outcomes) will be entered into OpenClinica by a trained researcher and must be validated by a senior researcher (source data verification) before the DSC can update the adaptive model based on this outcome.

Procedures will ensure the safe acquisition, storage, and transmission of data. University computers and servers are all password protected and study data can only be accessed by researchers involved in the study or those involved in governance procedures. Primary research data and records will be retained in their original form for a minimum of 10 years after the study has been completed.

### Monitoring of harms

All adverse events will be collected and reported (see also adverse events section). All non-serious toxicities and harms, whether expected or not, will be recorded in the relevant case report form and sent to the study coordination centre within 1 month of the form being due. Fatal or life-threatening SAEs and SUSARs will be reported on the day that the local site is aware of the event. The SAE form asks for the nature of event, date of onset, severity, corrective therapies given, outcome, and causality (i.e. unrelated, unlikely, possible, probably, definitely). The responsible investigator will sign the causality of the event. Additional information will be sent within 5 days if the reaction has not been resolved at the time of reporting. An SAE form will be completed and sent to the study coordination centre for all SAEs within 24 h. However, relapse and death or hospitalisation due to substance use disorder that cannot reasonably be attributed to study medication, and hospitalisations for elective treatment of a pre-existing condition do not need reporting as SAEs. Any participants who experience serious and enduring harm or injury as a result of taking part in this study will be eligible to claim compensation.

### Monitoring and audit

A data monitoring committee (DMC) has been convened and will meet regularly to discuss study progress, recruitment targets and future planning. Members are Prof. Alex Baldacchino (chair), Prof. Eilish Gilvarry, and Dr. Hakim Dehbi. They are independent from the sponsor and competing interests and have the required expertise in clinical trials and management of opiate dependence. If a trial stopping rule was triggered, a DMC meeting would be convened to review the trial management group’s decision to stop/continue the trial.

The study will be subject to monitoring inspection and audit by Imperial College London Research Governance and Integrity Team under their remit as sponsor, the Study Coordination Centre, and other regulatory bodies to ensure adherence to GCP. Monitoring by the sponsor will be conducted after *n* = 6 participants have been consented and then at 6 monthly intervals.

### Dissemination policy

The study will be reported in accordance with the CONSORT guidelines. We plan to disseminate findings via local, national, and international conference presentations such as at meetings of the Society for Study of Addiction, British Association for Psychopharmacology, Royal College Of Psychiatrists to reach academic, clinical, service user, and public audiences. We will also disseminate the results in peer-reviewed journals and during training events for addiction services. The results of the study will be reported and disseminated in peer-reviewed scientific journals, and internal reports commensurate with their author eligibility guidelines. Written feedback will be given to study participants and referring services.

## Discussion

Despite increasing morbidity and mortality, there has been a lack of pharmacological innovations in managing opiate dependence, particularly in facilitating withdrawal and relapse prevention. Preclinical and clinical evidence suggest that baclofen, a GABA-B agonist and a medication licenced to treat muscle spasticity and used off-licence to treat alcoholism, has therapeutic potential in this indication. Given that both baclofen and methadone are sedative medications, and methadone is the most common opiate substitute medication used and from which most patients will detoxify from, it is important to first establish that they can be safely given together. This single-blind, adaptive, randomised, placebo-controlled ascending dose study of a single dose of baclofen in opiate-dependent individuals stably maintained on methadone is therefore critical to evaluate the safety of combining baclofen and methadone. The trial will investigate the impact of the combination on a range of objective and pharmacokinetic-pharmacodynamic measures. Based on our clinical experience and guidance from National guidelines [47–49], for a proof-of-concept trial evaluating the efficacy of baclofen during detoxification, safe combinations of at least 30 mg baclofen with a minimum of 60 mg/day of methadone will be required.

This study was developed prior to the COVID-19 pandemic, and the number of individuals with opiate dependence successfully leaving treatment has reduced significantly in the last 2 years compared with pre-pandemic levels. During the first UK lockdown in March 2020 when addiction services were required to work largely remotely, guidance was issued that patients should be maintained on their opiate substitute dose. In the face of remote working, vulnerability of many patients, the move to unsupervised consumption for some and the stress of the COVID-19 pandemic, this was felt to be the safest approach. Consequently, opiate detoxification was rarely undertaken during 2020, and the number leaving treatment ‘opiate free’ has further declined (25%) whilst opiate deaths have increased by 20% [83]. As addiction services begin to support detoxification again it is vital that we explore approaches to pharmacologically optimise the process to improve these poor outcomes and reverse the decline in the number of opiate-dependent people leaving treatment free of opiates.

### Trial status

Current protocol version: 1.2, 15 March 2022

EudraCT number: 2021-002556-36

First patient, first visit: 11 January 2022

Anticipated last patient, last visit: 31 October 2022

## Supplementary Information

**Additional file 1.** Statistical analysis plan.

**Additional file 2.** Exploratory objectives, hypotheses, outcomes and planned analyses.

## Data Availability

Publications will be made available in the public domain. The study team will retain the exclusive use of data until the publication of major outputs has been completed. After this, data access including the full protocol, statistical codes, and participant-level data will be made available upon reasonable request to the CI, in accordance with the Data Protection Act.
