# Supplementary material for: FORWARDS-1: an adaptive, single-blind, placebo-controlled ascending dose study of acute baclofen on safety parameters in opioid dependence during methadone-maintenance treatment—a pharmacokinetic-pharmacodynamic study
Source: Trials. 2022 Oct 18;23:880. doi: 10.1186/s13063-022-06821-9 (PMC9579625; doi:10.1186/s13063-022-06821-9)
Supplement: Supplementary file 1 — Additional file 1. Statistical analysis plan. [file 13063_2022_6821_MOESM1_ESM.pdf]

FORWARDS-1 TRIAL

Evaluating the safety of acute baclofen in methadone-maintained individuals with opiate dependence

Statistical Analysis Plan

Version 2.0

Date: 09/09/2022

EudraCT Number: 2021-002556-36

REC Number: 21/WS/0080

Authors: Rachel Phillips, Suzie Cro, Pavel Mozgunov

Chief Investigator
Professor Anne Lingford-Hughes
Address: Department of Brain Sciences, Faculty of Medicine, Imperial College London, Commonwealth Building, Hammersmith Campus
Email: anne.lingford-hughes@imperial.ac.uk
Electronic signature: Anne Lingford-Hughes Digitally signed by Anne Lingford-Hughes Date: 2022.09.26 17:34:17 +01'00'
Date:

Senior statisticians:
Dr Suzie Cro
Address: Imperial Clinical Trials Unit, School of Public Health, Imperial College London, Stadium House, 68 Wood Lane
London, W12 7RH
Email: s.cro@imperial.ac.uk
Electronic signature:
Date:

Dr Pavel Mozgunov
Address: MRC Biostatistics Unit, East Forvie Building, University of Cambridge, Cambridge, CB2 0SR
Email: pavel.mozgunov@mrc-bsu.cam.ac.uk
Electronic signature:
Date:

Trial statistician
Dr Rachel Phillips
Address: Imperial Clinical Trials Unit, School of Public Health, Imperial College London, Stadium House, 68 Wood Lane
London, W12 7RH
Email: r.phillips@imperial.ac.uk

Data Monitoring Committee Statistician
Dr Hakim-Moulay Dehbi
Address: Comprehensive CTU at UCL, Institute of Clinical Trials & Methodology, Faculty of Population Health Sciences
Email: h.dehbi@ucl.ac.uk
Signature:
Date:

Document version history log

Date	Version	Changes made
17/01/2022	1.0	
09/09/2022	2.0	Minor amendments to wording of the estimand components and updates to template tables

Chief Investigator: Professor Anne Lingford-Hughes

Co-Investigators: Dr Louise Paterson, Dr Suzie Cro, Dr Claire Smith, Dr Sue Paterson, Dr Pavel Mozgunov

Study co-ordination centre:
Neuropsychopharmacology Unit
Centre for Psychiatry
Division of Brain Sciences
2nd floor Commonwealth Building
Du Cane Road
London W12 0NN
Tel: 0207 5947028

Contents

1. Description of the trial 12
1.1 Research objectives 12
1.1.1 Primary objective 12
1.1.2 Primary outcome 12
1.1.3 Secondary objectives 13
1.1.4 Secondary outcomes 14
1.1.5 Exploratory objectives 15
1.2 Trial Design 16
1.2.1 Description of overall study design 16
1.2.2 Study visits 16
1.3 Study population 18
1.3.1 Inclusion Criteria 19
1.3.2 Exclusion Criteria 19
1.4 Treatment allocation 20
1.5 Baclofen dose decision 21
1.6 Evaluation window 22
1.7 Evaluable patient 22
1.8 Data collection 23
1.9 Sample size estimation 23
1.10 Method of randomisation 23
1.11 Blinding 23
1.12 Brief description of proposed analysis 23
1.12.1 Analysis sets 24
2. Dose toxicity model 25
3. Estimands 27
3.1 Primary estimand 27
3.2 Secondary estimands 27
4. Data analysis plan 30
4.1 Recruitment and participant flow 30
4.2 Baseline characteristics 30
4.3 Withdrawals, loss to follow-up and other missing data 30
4.4 Adherence 30
4.5 Descriptive statistics for outcome measures 30
4.5.1 Primary outcome 30
4.5.2 Secondary outcomes 31
4.5.2.1 Components of DLT 31

4.5.2.2 Respiratory measures for signs of sub-threshold respiratory depression 36
4.5.2.3 Sedation measures 37
4.5.2.4 Symptom measures 38
4.6 Adverse events and serious adverse events 39
4.7 Missing data 39
5. Software 41
6. Appendix 1: Template tables and figures 42
Figure A.1: Participant flow 43
Table A.1: Randomisation by treatment arm 44
Table A.2: Baseline characteristics by treatment arm 45
Table A.3: Drug and alcohol diagnoses by treatment arm 48
Table A.4: Other medical diagnoses by treatment arm 49
Table A.5: Withdrawals (including lost to follow-up) by treatment arm 51
Table A.6: Reasons for withdrawal by treatment group 51
Table A.7: Estimated methadone ranges for each recommended baclofen dose as per model output* 52
Table A.8: Participants recommended Baclofen doses subject to escalation constraints* 53
Table A.9: DLT probabilities for hypothetical scenarios 54
Table A.10: Participants prescribed and received doses as per DSC recommendations and DLT status 55
Table A.11: Adherence to prescribed dose 56
Figure A.2: Baclofen escalation/de-escalation path over the course of the trial 57
Table A.12: DLT status by baclofen dose 58
Table A.13: DLT status by baclofen dose 58
Table A.14: DLT status by gender for baclofen participants 58
Table A.15: Stimulus intervention level by treatment arm 59
Table A.16: Stimulus intervention level by baclofen dose 61
Table A.17: Stimulus intervention level by gender 63
Table A.18: NEWS2 score summaries by treatment arm 65
Table A.19: Treatment effect estimates for NEWS2 scores at each time-point from the linear mixed effects model (model 1) 66
Table A.20: NEWS2 score summaries by baclofen dose 67
Table A.21: Treatment effect estimates for NEWS2 scores at each time-point from the linear mixed effects model (model 2) 68
Table A.22: NEWS2 score summaries by gender 69
Table A.23: Correlation between NEWS2 scores and methadone dose by treatment arm 70

Table A.24: Estimates of methadone effect on NEWS2 scores between treatment arms from the linear mixed effects model (model 1) 71
Figure A.3 Individual participant trajectories of NEWS2 scores over time by treatment arm 72
Figure A.4 Individual participant trajectories of NEWS2 scores over time by baclofen dose 72
Figure A.5 Individual participant trajectories of NEWS2 scores over time by gender 73
Figure A.6 Summaries of NEWS2 scores over time by treatment arm 74
Figure A.7 Summaries of NEWS2 scores over time by baclofen dose 74
Figure A.8 Summaries of NEWS2 scores over time by gender 75
Figure A.9 Scatterplot of NEWS2 scores and methadone dose at 120 minutes 75
Table A.25: GCS score summaries by treatment arm....................76
Table A.26: Treatment effect estimates for GCS scores at each time-point from the linear mixed effects model (model 1) 77
Table A.27: GCS score summaries by baclofen dose....................78
Table A.28: Treatment effect estimates for GCS scores at each time-point from the linear mixed effects model (model 2) 79
Table A.29: GCS score summaries by gender....................80
Table A.30: Correlation between GCS scores and methadone dose by treatment arm 81
Table A.31: Estimates of methadone effect on GCS scores between treatment arms from a linear mixed effects model (model 1) 82
Figure A.10 Individual participant trajectories of GCS scores over time by treatment arm83
Figure A.11 Individual participant trajectories of GCS scores over time by baclofen dose83
Figure A.12 Individual participant trajectories of GCS scores over time by gender...........83
Figure A.13 Summaries of GCS scores over time by treatment arm....................83
Figure A.14 Summaries of GCS scores over time by baclofen dose....................83
Figure A.15 Summaries of GCS scores over time by gender 83
Figure A.16 Scatterplot of GCS scores and methadone dose at 120 minutes 83
Table A.32: QTc summaries by treatment arm....................84
Table A.33: Treatment effect estimates for QTc scores at each time-point from the linear mixed effects model (model 1) 84
Table A.34: QTc summaries by baclofen dose....................85
Table A.35: Treatment effect estimates for QTc scores at each time-point from the linear mixed effects model (model 2) 85
Table A.36: QTc summaries by gender 86
Table A.37: Correlation between QTc scores and methadone dose by treatment arm........87
Table A.38: Estimates of methadone effect on QTc between treatment arms from a linear mixed effects model (model 1) 88
Figure A.17 Individual participant trajectories of QTc over time by treatment arm...........89

Figure A.18 Individual participant trajectories of QTc over time by baclofen dose 89
Figure A.19 Individual participant trajectories of QTc over time by gender 89
Figure A.20 Summaries of QTc over time by treatment arm 89
Figure A.21 Summaries of QTc over time by baclofen dose 89
Figure A.22 Summaries of QTc over time by gender...89
Figure A.23 Scatterplot of GCS scores and methadone dose at 120 minutes 89
Table A.39: Oxygen saturation (SpO2) levels by treatment arm...90
Table A.40: Treatment effect estimates for oxygen saturation (SpO2) levels at each time-point from the linear mixed effects model (model 1) 91
Table A.41: Oxygen saturation (Sp02) levels by baclofen dose..92
Table A.42: Treatment effect estimates for oxygen saturation (Sp02) at each time-point from the linear mixed effects model (model 2) 93
Table A.43: Oxygen saturation (Sp02) levels by gender...94
Table A.44: Correlation between oxygen saturation levels and methadone dose by treatment arm 96
Table A.45: Estimates of methadone effect on oxygen saturation (SpO2) levels between treatment arms from a linear mixed effects model (model 1)...97
Figure A.24 Individual patient trajectories of oxygen saturation (Sp02) levels over time by treatment arm 98
Figure A.25 Individual patient trajectories of oxygen saturation (Sp02) levels over time by baclofen dose 98
Figure A.26 Individual patient trajectories of oxygen saturation (Sp02) levels over time by gender..98
Figure A.27 Summaries of oxygen saturation (Sp02) levels over time by treatment arm...98
Figure A.28 Summaries of oxygen saturation (Sp02) levels over time by baclofen dose...98
Figure A.29 Summaries of oxygen saturation (Sp02) levels over time by gender..............98
Figure A.30 Scatterplot of oxygen saturation (Sp02) levels and methadone dose at 120 minutes..98
Table A.46: tcCO_2 by treatment arm 99
Table A.47: Treatment effect estimates for tcCO_2 levels at each time-point from the linear mixed effects model (model 1) 100
Table A.48: tcCO_2 by baclofen dose...101
Table A.49: Treatment effect estimates for tcCO_2 levels at each time-point from the linear mixed effects model (model 2) 102
Table A.50: tcCO_2 by gender...103
Table A.51: Correlation between tcCO_2 levels and methadone dose by treatment arm 105
Table A.52: Estimates of methadone effect on tcCO_2 levels between treatment arms from a linear mixed effects model (model 1) 106
Figure A.31 Individual patient trajectories of tcCO_2 over time by treatment arm 107
Figure A.32 Individual patient trajectories of tcCO_2 over time by baclofen dose............107

Figure A.33 Individual patient trajectories of $tcCO_2$ over time by gender 107
Figure A.34 Summaries of $tcCO_2$ over time by treatment arm..107
Figure A.35 Summaries of $tcCO_2$ over time by baclofen dose..107
Figure A.36 Summaries of $tcCO_2$ over time by gender 107
Figure A.37 Scatterplot of $tcCO_2$ levels and methadone dose at 120 minutes 107
Table A.53: Respiratory rate by treatment arm 108
Table A.54: Treatment effect estimates for respiratory rate at each time-point from the linear mixed effects model (model 1) 109
Table A.55: Respiratory rate by baclofen dose..110
Table A.56: Treatment effect estimates for respiratory rate at each time-point from the linear mixed effects model (model 2) 111
Table A.57: Respiratory rate by gender...112
Table A.58: Correlation between respiratory rate and methadone dose by treatment arm 114
Table A.59: Estimates of methadone effect on respiratory rate between treatment arms from a linear mixed effects model (model 1)..115
Figure A.38 Individual patient trajectories of respiratory rates over time by treatment arm 116
Figure A.39 Individual patient trajectories of respiratory rates over time by baclofen dose 116
Figure A.40 Individual patient trajectories of respiratory rates over time by gender........116
Figure A.41 Summaries of respiratory rates over time by treatment arm 116
Figure A.42 Summaries of respiratory rates over time by baclofen dose..........................116
Figure A.43 Summaries of respiratory rates over time by gender 116
Figure A.44 Scatterplot of respiratory rates and methadone dose at 120 minutes 116
Table A.60: Measures of respiratory function by treatment arm......................................117
Table A.61: Measures of respiratory function by baclofen dose 118
Table A.62: Measures of respiratory function by gender 119
Table A.63: Respiratory measures indicating sub-threshold respiratory depression by treatment arm 120
Table A.64: Respiratory measures indicating sub-threshold respiratory depression by baclofen dose 121
Table A.65: Respiratory measures indicating sub-threshold respiratory depression by gender..122
Table A.66: Total SHAS score by treatment arm 123
Table A.67: Treatment effect estimates for total SHAS scores at each time-point from the linear mixed effects model (model 1) 124
Table A.68: Total SHAS by baclofen dose..125
Table A.69: Treatment effect estimates for total SHAS score at each time-point from the linear mixed effects model (model 2) 126
Table A.70: Total SHAS score by gender 127

Table A.71: Correlation between total SHAS score and methadone dose by treatment arm 128

Table A.72: Estimates of methadone effect on total SHAS score between treatment arms from a linear mixed effects model (model 1)....129

Figure A.45 Individual patient trajectories of total SHAS scores over time by treatment arm 130

Figure A.46 Individual patient trajectories of total SHAS scores over time by baclofen dose 130

Figure A.47 Individual patient trajectories of total SHAS scores over time by gender 130

Figure A.48 Summaries of total SHAS scores over time by treatment arm....130

Figure A.49 Summaries of total SHAS scores over time by baclofen dose 130

Figure A.50 Summaries of total SHAS scores over time by gender 130

Figure A.51 Scatterplot of total SHAS score and methadone dose at 120 minutes....130

Table A.73: DEQ **liking** score by treatment arm....131

Table A.74: DEQ **wanting more** score by treatment arm....132

Table A.75: Treatment effect estimates for DEQ **liking** score at each time-point from the linear mixed effects model (model 1) 133

Table A.76: Treatment effect estimates for DEQ **wanting more** score at each time-point from the linear mixed effects model (model 1) 133

Table A.77: DEQ **liking** by baclofen dose 134

Table A.78: DEQ **wanting more** by baclofen dose 135

Table A.79: Treatment effect estimates for DEQ **liking** at each time-point from the linear mixed effects model (model 2) 136

Table A.80: Treatment effect estimates for DEQ **wanting more** at each time-point from the linear mixed effects model (model 2) 136

Table A.81: DEQ **liking** score by gender....137

Table A.82: DEQ **wanting more** score by gender 138

Table A.83: Correlation between DEQ **liking** and methadone dose by treatment arm....139

Table A.84: Correlation between DEQ **wanting more** and methadone dose by treatment arm 139

Table A.85: Estimates of methadone effect on DEQ **liking** between treatment arms from a linear mixed effects model (model 1) 140

Table A.86: Estimates of methadone effect on DEQ **wanting more** between treatment arms from a linear mixed effects model (model 1)....140

Figure A.52 Individual patient trajectories of DEQ **liking scores** over time by treatment arm 141

Figure A.53 Individual patient trajectories of DEQ **wanting more** scores over time by treatment arm 141

Figure A.54 Individual patient trajectories of DEQ **liking scores** over time by baclofen dose 141

Figure A.55 Individual patient trajectories of DEQ **wanting scores** over time by baclofen dose 141
Figure A.56 Individual patient trajectories of DEQ **liking scores** over time by gender ...141
Figure A.57 Individual patient trajectories of DEQ **wanting scores** over time by gender141
Figure A.58 Summaries of DEQ **liking scores** over time by treatment arm 141
Figure A.59 Summaries of DEQ **wanting more** scores over time by treatment arm 141
Figure A.60 Summaries of DEQ **liking** scores over time by baclofen dose......................141
Figure A.61 Summaries of DEQ **wanting more** scores over time by baclofen dose........141
Figure A.62 Summaries of DEQ **liking** over time by gender...142
Figure A.63 Summaries of DEQ **wanting more** scores over time by gender...................142
Figure A.64 Scatterplot of DEQ **liking** and methadone dose at 120 minutes 142
Figure A.65 Scatterplot of DEQ **wanting more** and methadone dose at 120 minutes 142
Table A.87: Summary of adverse event type by treatment arm 143
Table A.88: Adverse events by preferred term and treatment arm...................................144
Table A.89: Adverse events by preferred term, severity grading and treatment arm........145
Figure A.66: Stacked bar chart of adverse event severity 145
Table A.90: Adverse events by preferred term and treatment arm...................................146
Figure A.67: Dot plot of adverse events 147
Table A.91: Serious adverse event listings for individual participants 148
References...149

1. Description of the trial

Facilitating Opiate Recovery: Withdrawal and Abstinence through Detoxification Support study 1 (FORWARDS-1) is an adaptive, single-blind, randomised (3:1), parallel group, placebo-controlled ascending dose study of acute baclofen (10mg, 30mg, 60mg, 90mg) on safety parameters in opioid dependence during methadone-maintenance treatment. The safety parameters of taking baclofen in combination with methadone will be assessed using a Bayesian dose-combination toxicity model (see Section 2), which will inform dose-escalation continuously throughout the trial. See protocol version 1.0.

1.1 Research objectives

1.1.1 Primary objective

To determine whether we can safely proceed prescribing a minimum of 30mg baclofen to clients receiving a range of doses of methadone (a minimum of 60mg) through determination of lack of CNS depressant activity using measures of respiratory function, cardiovascular function and sedation as follows:

- Respiratory function (respiration rate, oxygen saturation, end-tidal and/or transcutaneous CO_2)
- Requirement for intervention (scored according to step-wise algorithm)
- Cardiovascular function (ECG, blood pressure, heart rate)
- Sedation (rating scales, National Early Warning Score- NEWS2, Glasgow Coma Scale)

Our minimum acceptable dose is 30mg baclofen to be safely prescribed in those receiving maintenance doses of methadone up to and including 60mg daily. Baclofen doses below 30mg/d have not demonstrated efficacy in alcoholism (Agabio et al., 2018), and although the minimum daily recommended doses outlined in Orange and NICE guidelines is 60mg/d methadone, the majority of clients in our services are on methadone doses lower than 60mg/d (average 56.3mg/d), and our target population for those on detoxification or tapering pathways are on even lower doses (i.e. <40mg/day with methadone estimated average of 25mg). Therefore, these ‘acceptable minimums’ will still capture the majority of clients that we wish to enrol.

Our target dose is for 90mg baclofen to be safely prescribed in those receiving maintenance doses of methadone up to and including 120mg daily. This would provide the full range of prescribing freedom within current guidelines for prescribing of baclofen in spasticity (BNF guidelines, max 100mg/day), and meets recommendations for off-label prescribing in alcoholism (max 80mg/day granted for French temporary license (Rolland et al., 2020), and recommended efficacious doses 30-60mg/day (Agabio et al., 2018). This is also in line with prescribing of methadone as opiate substitution therapy (OST) according to Department of Health ‘Orange’ and NICE guidelines, both of which recommend 60mg/day of methadone as clinical therapeutic target with a maximum of 120mg/d methadone.

1.1.2 Primary outcome

Primary outcome: The maximum safe dose(s) of baclofen at which 15-25% of evaluable participants experience a dose limiting toxicity (DLT) for prescribed doses of methadone, where a DLT is defined in section 1.1.2.1 and is comprised of the following components:

1) Intervention level (0 to 4) as described in section 1.1.2.2
2) National Early Warning Score (NEWS2), measured at discrete time-points
3) Glasgow Coma Scale (GCS) score, measured at discrete time-points
4) QTc on ECG trace, measured at discrete time-points
5) Measures of respiratory function, measured continuously at discrete time-points
   a) Oxygen saturation (SPO_2)
   b) Respiratory (ventilation) rate
   c) Incidence of apnoea

Definitions of the evaluation window and evaluable participants can be found in sections 1.6 and 1.7 respectively, and the discrete time-points are given in section 1.2.2.

There may be a single maximum safe dose of baclofen across tested doses of methadone 0-120mg or the maximum safe dose of baclofen may vary by methadone dose.

1.1.2.1 Definition of dose-limiting toxicity (DLT)

A 'dose limiting toxicity (DLT)' is defined as at least one of the following:

1) Situation requiring intervention level ≥4 (section 1.1.2.2) at any time
2) NEWS2 score >4 or score of 3 in any parameter (threshold for trigger of urgent ward-based response)
3) Measures of respiration with a persistent change in at least one of:
   a) Reduction in SPO_2 [≤91% for more than 30 seconds or >5% reduction in SpO_2 for more than 30 seconds
   b) Reduced respiratory rate (≤8/min)
   c) Absence of inspiratory airflow for >30s combined with a sustained fall in SpO_2
4) GCS score <12
5) Persistent QTc prolongation (>500ms or increase of >60ms; if the initial QTc value at any time-point is prolonged, the ECG should be repeated two more times- with 5 minutes between ECG readings- and the average of the 3 QTc values used to determine DLT).

1.1.2.2 Stimulus Intervention levels

If marked sedation or apnoea >30s occurs, the following levels of stimulus intervention will be utilised and scored accordingly (0; no intervention).

1) Indirect noise e.g. door opening, closure, cough etc
2) Interrupt patient with direct speech
3) Touch
4) Unable to rouse patient with touch

Any intervention at level 4 or above will meet criteria for a DLT and a clinical decision will be made as to further action.

1.1.3 Secondary objectives

Secondary aims will be investigated and findings used to inform the design of the subsequent proof-of-concept efficacy study 2 and will include:

i. To identify whether there is any evidence of sub-threshold DLT respiratory, cardiovascular or sedation changes in response to baclofen relative to placebo.

ii. To determine whether there is any evidence of abuse liability signal for baclofen relative to placebo, in combination with methadone, through determination of lack of abuse liability as measured by the drug effects questionnaire (DEQ)

iii. To determine whether there is evidence of reduced sensitivity to baclofen relative to placebo through determination of Subjective drug response (Subjective High Assessment scale, SHAS).

iv. To explore the variability in response to baclofen at different baclofen dose levels for (a) CNS depressant activity using measures of respiratory function, cardiovascular function and sedation, (b) abuse liability measured by DEQ and (c) subjective drug response (SHAS)

v. To explore the variability in response to baclofen at different methadone levels for (a) CNS depressant activity using measures of respiratory function, cardiovascular function and sedation, (b) abuse liability measured by DEQ and (c) subjective drug response (SHAS)

vi. To explore the variability in response to baclofen by gender for (a) CNS depressant activity using measures of respiratory function, cardiovascular function and sedation, and (b) abuse liability measured by DEQ and (c) subjective drug response (SHAS)

1.1.4 Secondary outcomes

1) Components of DLT for baclofen and placebo groups, and by baclofen dose group and gender:

i. Intervention level (0 to 4) as described in section 1.1.2.2
ii. National Early Warning Score (NEWS2), measured at discrete time-points
iii. Glasgow Coma Scale (GCS) score, measured at discrete time-points
iv. QTc on ECG trace, measured at discrete time-points
v. Measures of respiratory function, measured continuously at discrete time-points:
   a) Oxygen saturation (SpO_2) - reduction in SPO2 [≤91% for more than 30 seconds or >5% reduction in SpO2 for more than 30 seconds
   b) Respiratory (ventilation) rate - reduced respiratory rate (≤8/min)
   c) Incidence of apnoea - absence of inspiratory airflow for >30s combined with a sustained fall in SpO2

2) Additional respiratory measures for signs of sub-threshold respiratory depression for baclofen and placebo groups, and by baclofen dose group and gender:

i. SpO_2 - instances of <92% or of >5% reduction for more than 10 seconds
ii. CO_2 - instances of $tcCO_2$% exceeding a partial pressure CO_2 increase by 1kPa (advice from respiratory physician)
iii. Respiratory rate- instances of absence of inspiratory airflow for more than 10 seconds or respiratory rate drops <9/min
iv. Time course of SpO_2, CO_2 and respiratory rate following baclofen dosing, relative to placebo.

3) Sedation measures for baclofen and placebo and by baclofen dose level and gender as measured by the T-SHAS score (total score on Subjective High Assessment Scale)

a) Mean Total-SHAS score at peak PD response (2-3h)
b) Time-course of T-SHAS

4) Symptom measures for baclofen and placebo and by each baclofen dose level and gender as measured by the Drug Effects Questionnaire (DEQ)
    a) Mean ‘Drug liking’ and ‘want more’ scores at peak PD response (2-3h)
    b) Time-course of DEQ scale

5) Correlation between methadone levels and (a) CNS depressant activity using measures of respiratory function, cardiovascular function and sedation, (b) abuse liability measured by DEQ and (c) subjective drug response (SHAS)

1.1.5 Exploratory objectives

Additional exploratory aims will be investigated by the clinical team. As analysis of these outcomes will not be undertaken by the statistical team exploratory outcomes and analysis details are omitted from this statistical analysis plan. Please see protocol version 1.0 for full details.

1.2 Trial Design

1.2.1 Description of overall study design

This will be a single-blind, adaptive, randomised, parallel group, placebo-controlled ascending dose study of a single dose of baclofen in opiate-dependent individuals stably maintained on methadone. Participants will be randomised in a 3:1 ratio to baclofen or placebo. Participants allocated to baclofen will be dosed (10, 30, 60 or 90mg) in groups of up to 3, with a maximum available sample size of 64 (up to 48 on baclofen and 16 on placebo). An adaptive model (see Section 2) will inform the dosage of baclofen for each patient group based on the trial data accumulated to date and the participant's methadone dose. The dose setting committee (DSC) retains the ability to override the model's dose recommendation if clinically indicated.

Following randomisation, participants will attend the Imperial Clinical Research Facility (ICRF) for a single experimental visit, during which they will consume their usual daily dose of methadone under observation soon after arrival, followed by an acute oral dose of baclofen or placebo approximately 1 hour later, as determined by the DSC. Measures of respiratory, cardiovascular, sedation and PK will be obtained periodically for up to 5 hours after baclofen dose. The following day, participants will receive a follow-up phone call to check welfare to complete DLT follow-up and will be required to return their actiwatch via pre-paid envelope, or to their local addiction service, as appropriate.

1.2.2 Study visits

All eligible participants will have:

- Pre-screening assessment by telephone or in their usual clinical addiction service, as appropriate
- One in-person clinical screening visit.
- Randomisation/enrolment
- One in-person experimental study visit (see Figure 2*)
- One follow-up phone-call following the experimental study visit to check for adverse events.

Figure 1: Study procedure schematic

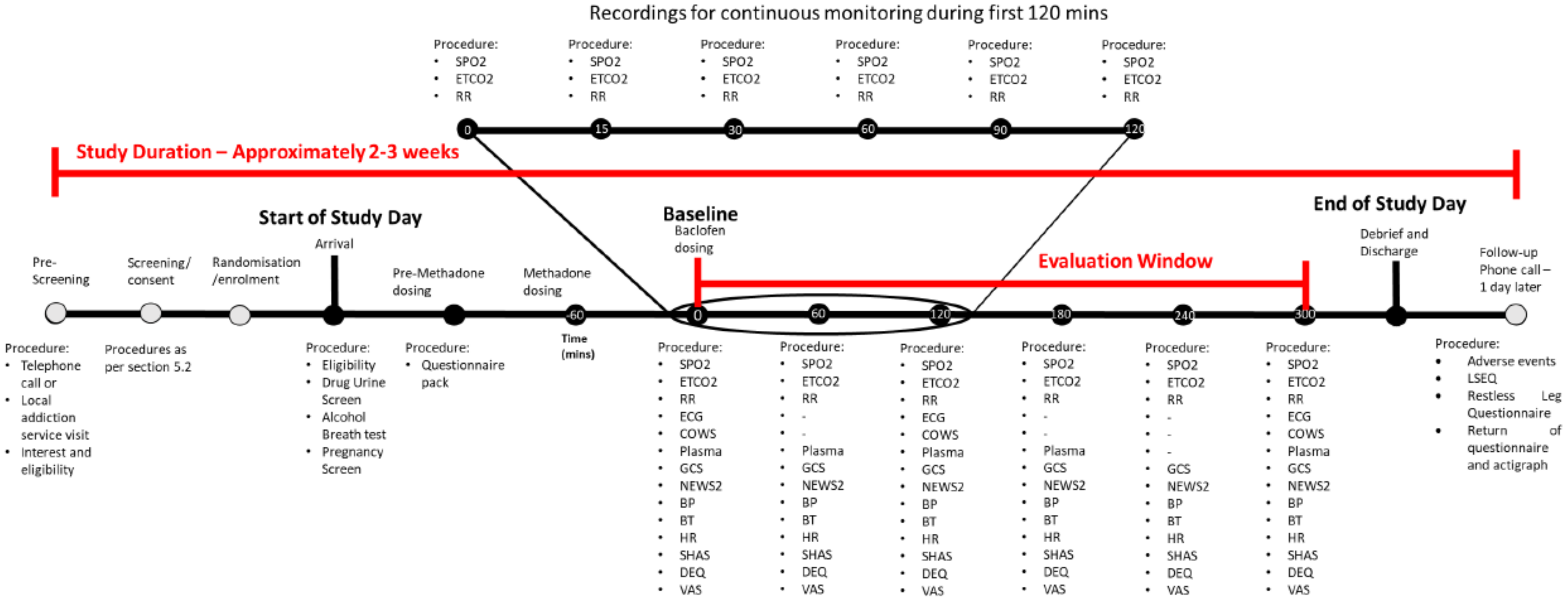

Key: **SPO2**; Blood Oxygen Saturation, **ETCO2**; End Tidal Carbon Dioxide, **RR**; Respiration Rate, **ECG**; Electrocardiogram, **COWS**; Clinical Opiate Withdrawal Scale, **Plasma**; Blood Sample, **GCS**; Glasgow Coma Scale, **NEWS2**; National Early Warning Score 2, **BP**; Blood Pressure, **BT**; Body Temperature, **HR**; Heart Rate, **SHAS**; Subjective high assessment scale, **DEQ**; Drug Evaluation, Questionnaire, **VAS**; Visual Analogue Scale, **LSEQ**; Leeds Sleep Evaluation Questionnaire.

Figure 2: Experimental visit schematic

*Experimental study day duration will be approximately 6-7 hours. Start times will be determined according to participant's preference but a typical study day would look like this:

Procedure	**Timepoint (mins)**								
	#Baseline	15	30	60	90	120	180	240	300
NEWS2	x	-	-	x	-	x	x	x	x
SpO_2	x	x*	x*	x	x*	x	x	x	x
RR	x	x*	x*	x	x*	x	x	x	x
BP	x	-	-	x	-	x	x	x	x
HR	x	-	-	x	-	x	x	x	x
Body Temp	x	-	-	x	-	x	x	x	x
LOC	x	-	-	x	-	x	x	x	x
GCS	x	-	-	x	-	x	x	x	x
ECG	x	-	-	-	-	x	-	-	x
CO_2	x	x	x	x	x	x	x	x	x
SHAS	x	-	-	x	-	x	x	x	x
DEQ	x	-	-	x	-	x	x	x	x
VAS	x	-	-	x	-	x	x	x	x
Blood Plasma	x	-	-	x	-	x	x	-	x
COWS	x	-	-	-	-	x	-	-	x

#Baseline refers to measurements obtained prior to Time 0, which represents the time at which baclofen/placebo is administered. Shaded area are measures comprising the NEWS2 score. *At these time points SpO_2 and RR measures will be taken in addition to those associated with the NEWS2.

1.3 Study population

Opiate dependent participants engaged in treatment for their opiate dependence from a specialist community addiction service and receiving stable doses of opiate substitution therapy with methadone. Therefore, the participants will be regularly attending an addiction service, receiving psychosocial support. Therefore, participants will not be chaotic with regard to their drug/alcohol use, unreliable in their attendance at appointments or have substantial physical or mental health needs.

In particular, the research team will endeavour to recruit participants from across the methadone-maintenance dose range of 1 to 120mg, including both males and females, to maintain a balanced design and to capture data that allows a representative assessment of safety across the clinical population.

1.3.1 Inclusion Criteria

1. Aged over 21
2. Willing and able to comply with protocol
3. Able to read, comprehend and record information written in English
4. Capable of giving written informed consent, which includes compliance with the requirements and restrictions listed in the consent form.
5. Healthy as determined by a responsible physician, based on a medical evaluation which includes medical history, a physical examination, laboratory tests (if required), and a psychiatric evaluation. A volunteer with clinical parameters outside the reference range for the population being studied may be included, only if the investigators concur that the finding is unlikely to jeopardize either subject safety or study integrity.
6. DSM-5 diagnosis of current severe opioid use disorder
7. Treated with methadone substitution therapy and able to maintain the same stable dose for screening and experimental visit.
8. Ability to receive an acute dose of up to 90mg baclofen or up to 4800IU vitamin D (placebo).

1.3.2 Exclusion Criteria

An individual will not be eligible for inclusion in this study if any of the following criteria apply:

1. Intoxication on any of the visits, as assessed by difficulty in walking, the slurring of speech, difficulty concentrating or drowsiness. This exclusion criteria would exclude a subject from that study day only and not the whole study, at the discretion of the research team.
2. Positive urine drug screens or breath alcohol at screening or experimental testing visits. A minimum list of drugs that will be screened for include amphetamines, cocaine, opiates, methadone, cannabinoids and benzodiazepines. Positive results for methadone will be allowed for those opiate dependent participants still undergoing OST. Positive results for cannabinoids will be allowed given the long half-life of cannabinoid metabolites. This exclusion criteria would exclude a subject from that study day only and not the whole study, at the discretion of the research team.
3. Current DSM-5 substance dependence disorder for any other substance except for opiates and nicotine. Lifetime history of dependence on other substances will be allowed given very high incidence of co-dependence.
4. Regular on-top use of heroin or other opiates or other illicit substances in combination with OST, which in the opinion of the investigators will interfere with subject safety or study integrity.
5. Any participant taking over 120mg/day of prescribed methadone.
6. Current severe DSM-5 mental health disorder (excluding opiate dependence). Current moderate or mild DSM-5 depressive, anxiety, sleep or personality disorders will be allowed given the high levels of comorbidity, provided in the opinion of the investigators, the participant is able to complete study procedures satisfactorily.
7. Current or past history of enduring severe mental illness e.g. psychotic disorder (excluding drug induced), schizophrenia, bipolar affective disorder).
8. Active suicidality.
9. Use of regular prescription medications which in the opinion of the investigators will interfere with subject safety or study integrity. Regular use of psychotropic medication will be permitted e.g. antidepressants, provided the participant is compliant with administration and the investigators concur that they will not interfere with subject safety or study integrity.
10. Participants are taking any medication that is contraindicated with baclofen or placebo (vitamin D_3), or are hypersensitive to them or any of their excipients.
11. Participants that are taking any medication that in the opinion of the investigators may impact on the outcome measures during the experimental session.

12. Use of intermittent psychotropic medication which in the opinion of the investigators will interfere with subject safety or study integrity.
13. End stage or acute renal failure.
14. Severe chronic obstructive pulmonary disease (COPD) or Type 2 respiratory failure.
15. Pulse rate <40 or >100 BPM OR systolic blood pressure >160 and <100 and a diastolic blood pressure >95 and <60 in the semi-supine position.
16. Oxygen saturation <92% at rest
17. A screening ECG with a QTcB or QTcF > 500 msec or an ECG that is not suitable for QT measurements (e.g. poorly defined termination of the T-wave) and/or with another ECG abnormality which in the opinion of the study physician is clinically significant and represents a safety risk. Note that if the initial QTc value is prolonged, the ECG should be repeated two more times (with 5 minutes between ECG readings) and the average of the 3 QTc values used to determine eligibility.
18. Clinically significant head injury (e.g., requiring medical or surgical intervention) that in the opinion of the investigators, contraindicates their participation .
19. Active hepatitis or HIV.
20. Active peptic ulceration.
21. Significant current or past medical history that, in the opinion of the investigators, contraindicates their participation.
22. The subject has participated in a clinical trial and has received an investigational product within 30 days, 5 half-lives or twice the duration of the biological effect of the investigational product (whichever is longer) prior to the first experimental visit.
23. Pregnancy or breast-feeding
24. Unwillingness or inability to follow the procedures outlined in the protocol.

1.4 Treatment allocation

Participants will be randomised (single-blind) to baclofen or placebo in a 3:1 ratio. If allocated to baclofen, a Bayesian dose-escalation adaptive model will be used to determine the baclofen dose allocation and will be informed by the accumulating occurrences of dose-limiting toxicity (DLT) events at increasing doses of baclofen (10, 30, 60, 90mg) with the corresponding doses of methadone.

Figure 3: Schematic for baclofen dose determination

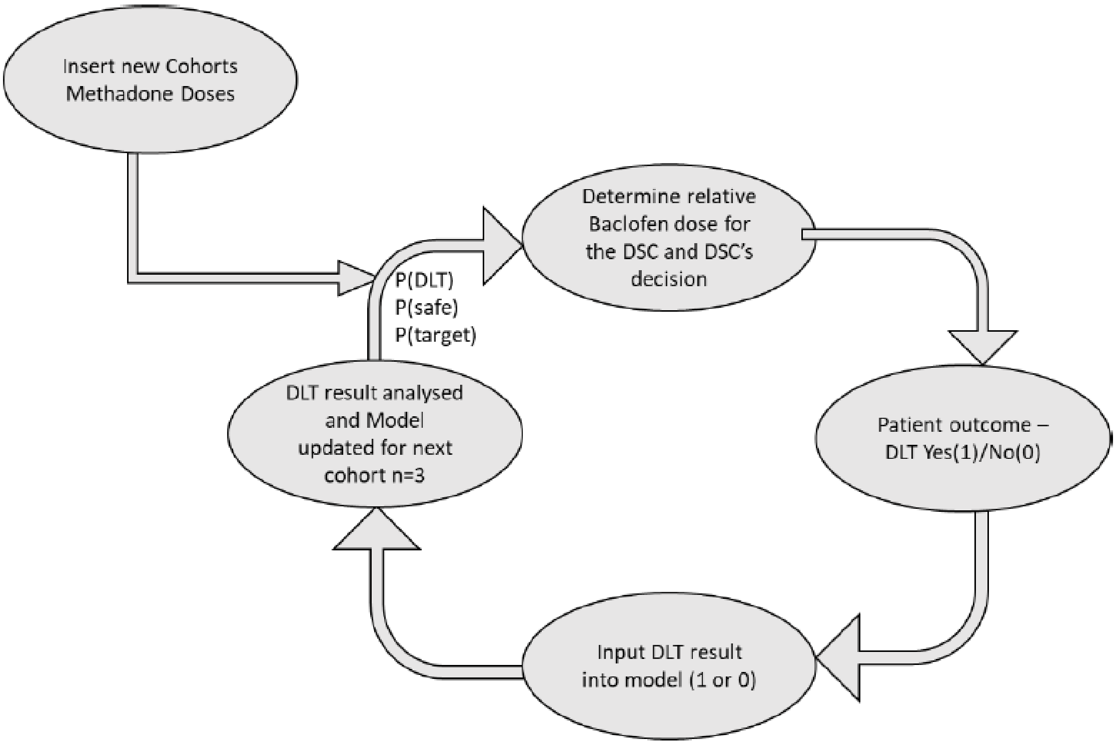

1.5 Baclofen dose decision

Starting dose

The lowest dose of baclofen (10mg) will be allocated to the first group of 3 participants assigned to receive baclofen.

Criteria for dose escalation

The doses of Baclofen to be potentially explored in the trial are 10, 30, 60 and 90mg. A Bayesian dose-toxicity model will be used to guide dose escalation (see Section 2 for full model specification). A DLT rate of 20% with an equivalence interval of 15-25% will be targeted in order to estimate the current maximum tolerated dose (MTD), conditional on the participant's methadone dose, in dose escalation.

The dose-toxicity model will be continuously updated after each group of at most 3 participants on baclofen, to recommend dosing for the subsequent group of participants.

Escalation constraints include (1) Doses of baclofen to be tested cannot be skipped, and (2) If any of the current cohort experience a DLT, then the next group of patients will be assigned to the current (or lower) dose of baclofen at which the DLT was experienced, without dose escalation, depending on the prescribed dose of methadone for the next individual that enters the study. If there is no DLT, a dose escalation may be advised by the model, again, depending on the prescribed dose of methadone for the next individual that enters the study.

The model will recommend to stop the trial earlier due to safety concerns if the probability that the risk of DLT for 60mg of methadone in combination with 30mg of baclofen exceeds the target range is 25% or more. The model will recommend to stop the trial earlier due to all doses of baclofen being safe if the probability that the risk of DLT for 120mg of methadone in combination with 90mg of baclofen does not exceed the target range is 92.5% or more, representing the ‘all-safe-stop criteria’.

The DSC (comprised of a statistician, clinician and member of the research team) will meet to discuss all baclofen dosing decisions, and can decide to over-ride any recommendation made by the adaptive model, if clinically indicated. Outside of the model parameters, clinical judgement will determine whether responses to baclofen are sufficiently concerning to cease escalation, escalate to a lower dose than planned by the model, or to reduce the average methadone dose for each dose of baclofen examined.

Should the study team witness multiple DLTs, using their clinical experience they can halt the trial early over safety concerns.

In the scenario that there is a very high probability that all doses are safe (90mg baclofen with up to and including 120mg methadone) before reaching the maximum recruitment of n=48 receiving baclofen, the study will retain the ability to override the all-safe-stop criteria to continue recruitment until we have tested a sufficient range of doses of methadone to ensure we have covered the clinical range of potential methadone doses and/or to explore baclofen dose separation to achieve maximum precision on secondary outcomes.

Given no safety concerns, we expect the *minimum* numbers of participants at each of the doses to be as follows: n=3 @10mg baclofen, n=3 @ 30mg baclofen, n=12 @ 60mg baclofen and n=12 @ 90mg baclofen, with n=10 receiving placebo, providing sufficient data to allow adequate exploration of dose separation on primary and secondary outcomes across a range of methadone dose levels, and an assessment of GABA-B receptor sensitivity and any gender effects. These numbers might be exceeded if the variability in response is larger than anticipated, female representation is lower than we would like, or we have not yet been able to test the full range of methadone doses. If a particular baclofen dose is deemed unsafe, then the lower doses would be similarly explored.

1.6 Evaluation window

The evaluation window for primary and secondary outcome measures will begin at dosing and end at 5 hours post-dose, with the exception of the ‘intervention level’ (as defined in the DLT definition) which will begin at dosing and continue until the last follow-up phone call. This call will be conducted the following day, and the window will be extended if the participant is experiencing sedation or other symptoms.

1.7 Evaluable patient

An evaluable participant is defined as one who has received study medication and has provided sufficient data to meet the primary endpoint of determining the presence or absence of a DLT, *and* sufficient data relating to the main secondary outcome measures. Whether sufficient data has been obtained for this purpose will be determined by clinical judgement on an individual participant basis.

In the unlikely event that a participant decided to self-discharge themselves after dosing but prior to the end of the 5 hour evaluation window, this participant could be counted as an evaluable patient with no DLTs provided they had completed the 2-3h time-point and that this self-discharge had occurred within a clinical picture of stable or normalising

observations. Attempts to complete the follow-up phone call would be made to confirm absence of DLT as defined by 'intervention level'.

In the event of an inability to acquire sufficient data, as defined above, the decision on whether to include an individual's data in the primary (DLT) or secondary analysis, or whether that individual would be replaced, would be made on a case by case basis.

1.8 Data collection

All data will be collected in a pseudo-anonymised and coded manner and stored within paper-based CRFs and/or via electronic data capture systems within purpose built secure-access online databases, OpenClinica and REDCap, or saved electronically on secure University (Imperial College) computer systems and facilities. Analysis described in this SAP relates to the data captured in the OpenClinica database.

1.9 Sample size estimation

The sample size is not fixed. Up to a total of 64 patients are planned to be enrolled in the study with up to 48 patients on the experimental doses and up to 16 on placebo, as recommended by the trial model in consultation with clinicians and the study team. The performance of the Bayesian dose-finding trial design based on 48 participants was assessed via simulations under several clinically relevant scenarios in terms of accuracy of the number of patients that would receive (i) their individual target dose combination and (ii) a combination that is safe for them in the subsequently planned phase 2 trial. Simulations confirm desirable operating characteristics with a sample size of 48; the average proportion of patients allocated to target doses was 62%-93%, and average proportion of patients allocated to safe doses was 74%-99%. Under unsafe scenario, the design terminates trial with probability 90%. (Mozgunov et al., 2021)

The drop-out rate from the study is estimated to be approximately 20% following screening. We therefore anticipate screening up to 100 methadone-maintained opioid dependent participants for entry into the study, with ~75% eligibility rate at screening, and a further ~20% drop out prior to, or at the experimental session.

1.10 Method of randomisation

The randomisation schedule will be generated by the trial statistician, using a 3:1 ratio (baclofen: placebo). Blocked randomisation will be used to maintain the 3:1 ratio throughout the study. Randomisation will be conducted via the online software application Sealed Envelope to maintain allocation concealment.

1.11 Blinding

This is a single blind study so only participants will be blind to their treatment allocation. The study clinicians and researchers will be aware of the drug allocation. Analysis described in this SAP will be undertaken by the trial statistician who will be unblind to treatment allocation.

1.12 Brief description of proposed analysis

No formal statistical testing will be conducted for this early phase study and no power calculation has been performed; the analyses for this study will be descriptive except for possible exploratory analyses as deemed appropriate. All statistical analyses for primary and secondary outcomes are to be viewed as exploratory. Across primary and secondary analyses, no imputation will be performed for missing data; analyses will be based on observed data only. If necessary, sensitivity analyses will be conducted to address the impact of any missing data on the primary outcome (see Section 4.7).

1.12.1 Analysis sets

Full analysis set

The full analysis set includes all randomised patients. This is equivalent to the ITT (intent-to-treat) population. The full analysis set will be used to summarise study conduct and patient disposition.

Safety set

The safety analysis set will consist of all subjects who are randomised and received study drug. Subjects in this analysis set will be used for demographic, baseline characteristics, and safety summaries. Patients will be grouped according to actual treatment received.

DLT evaluable safety set

The DLT evaluable safety analysis set will consist of all subjects who are randomised and received study drug and are evaluable for a DLT. Subjects in this analysis set will be used for the primary dose-combination toxicity response analysis (included in the Bayesian analysis model). Patients will be grouped according to actual treatment received.

The analysis set for each secondary outcome will be based on the subset of patients from the safety set for whom at least one measurable outcome has been obtained.

2. Dose toxicity model

The relationship between the dose of baclofen and methadone and the probability of observing a DLT will be modelled via a 5-parameter Bayesian logistic regression model with an interaction parameter as follows (Neuenschwander B, 2015).

The dose-toxicity models for each agent given as monotherapies are parametrised by a two-parameter logistic log-normal model with the actual dosage of the agent included directly in the model on the log-scale as;

$$logit\big(p(M)\big) = \alpha_{01} + \alpha_{11} \times \log(M/M_*)$$

$$logit\big(p(B_k)\big) = \alpha_{02} + \alpha_{12} \times \log(B_k/B_*)$$

where $M_* = 60$ and $B_* = 30$ are reference doses and with prior,

$$(\alpha_{0i}, \log(\alpha_{1i})) \sim N(\mu_i, \Sigma_i)$$

$$where\ (\mu_{01} = -3.75\ , \mu_{11} = 0.40) \text{ and } (\mu_{02} = -3.25\ , \mu_{12} = 0.05)$$

and

$$\Sigma_1 = \Sigma_2 = \begin{bmatrix} 0.5^2 & 0 \\ 0 & 0.35^2 \end{bmatrix}$$

The combination toxicity model under the assumption of independence of baclofen and methadone can be written as,

$$p_0(M, B_k) = 1 - (1 - p(M))(1 - p(B_k))$$

Including the interaction term in the combination-toxicity model, the model is as follows,

$$odds\big(p(M, B_k)\big) = odds(p_0(M, B_k)) \times exp\left(\eta \frac{M}{M_*} \frac{B_k}{B_*}\right)$$

where η is the interaction coefficient with prior,

$$\eta \sim N(0, 1.25)$$

Positive values of η correspond to synergistic toxicity, whilst negative values correspond to antagonistic toxicity, and zero corresponds to additive effect.

The parameters $\alpha_{01}, \alpha_{11}, \alpha_{02}, \alpha_{12}$ are the unknown quantities that define the combination-toxicity relationship. Using the prior distributions and data collected during the trial and Bayes theorem the posterior distribution of the parameters is sequentially updated using MCMC methods. The value of the hyper-parameters for prior distribution have been obtained via extensive calibrations over the number of qualitatively different and clinically relevant combination-toxicity scenarios. The chosen values result in a high proportion of correct individual MTD recommendation across many clinical scenarios (Mozgunov et.al. 2021).

The model will output the mean posterior probability of a patient experiencing a DLT (p(DLT)) at a given methadone-baclofen dose, the posterior probability of the given dose being safe (p(safe)), and the posterior probability of the dose being within the target toxicity interval (p(target)). For each given participant with a prescribed dose of methadone, doses of baclofen are deemed to be safe if the risk of DLT being at least 25% (the upper bound of the target interval), is less than 25%, where the latter bound was calibrated to result in appropriate safety properties of the model. The target toxicity interval is 15-25%.

The first cohort (4 participants) will be randomised to 10mg of baclofen or placebo (3:1 allocation ratio) with subsequent planned dose escalation of baclofen up to 90mg. The model will be sequentially updated after at most every 3 participants using the DLT/no DLT data from all previous cohorts in the trial before making the dose recommendation for the next cohort. The recommendation of the model will be to assign patients in the next cohort to their individual safe doses of baclofen that are most likely to have a toxicity risk of between 15% and 25% according to their prescribed dose of methadone subject to the escalation constraints. The escalation will occur only in the case of acceptable safety and tolerability at the next lowest dose of baclofen for the prescribed dose of methadone and if no DLTs are observed for the current cohort of participants. Doses of baclofen cannot be skipped. Dose escalation will be based on the review of safety data collected during the experimental visit and the subsequent follow-up phone call on the DLT evaluable safety set. Participants from cohort 2 or beyond may receive different doses of baclofen depending on their individual doses of methadone and previous data contributing to the model.

The model can recommend stopping the trial earlier with all doses of baclofen found to be safe, if the probability that the risk of toxicity at 90mg baclofen in combination with 120mg methadone is 25% or below, is above 92.5%. The trial can be recommended to be stopped earlier for safety concerns if the probability that the risk of toxicity at 30mg baclofen in combination with 60mg methadone (the lowest clinically viable combination) being above 25%, is 25% or more. These probability thresholds (92.5% and 25%) were found to result in accurate early termination while limited erroneous termination. Full model specifications can be found in Mozgunov et al., 2021.

Throughout the trial model results and recommendations will be shared with the clinical team after each model update undertaken by the statistical team. See '*template – model results_recommendations-report # v1.0.docx'* in 'S:\2021_FORWARDS_1\15. DSC_(non-TMF)\2. Report Template' for the template and example report. The DSC can over-rule the recommendation of the model in the light of other safety and tolerability information. After each report update is shared with the clinical team it will be archived and saved in a unique folder in 'S:\2021_FORWARDS_1\7. Analysis [to be locked]'. See '*FORWARDs participant flow and model recommendation process v1.0.docx'* in 'S:\2021_FORWARDS_1\15. DSC_(non-TMF)\1. DSC SOP' for model update processes.

3. Estimands

3.1 Primary estimand

The primary estimand for the primary objective described in Section 1.1.1 is as follows:

- **Population**: Opiate-dependent individuals stably maintained on methadone meeting the inclusion/exclusion criteria who are able to receive baclofen.
- **Treatment condition**: Baclofen at the dose received. The allocation dose is specific to the entry cohort as recommended by the DSC based upon the dose-combination toxicity model
- **Outcome**: Incidence of DLT, as defined in Section 1.1.2.1.
- **Handling of intercurrent events**:
    - Treatment not received - It is our intention to use the principal stratum strategy and target incidence of DLT only within the principal stratum (i.e. subset) of patients who would receive study treatment (baclofen at any dose). Thus only patients who receive treatment will be included in the analysis.
    - Dose received not as recommended – It is our intention to target the incidence of DLT for received doses (see treatment condition). Thus, if a dose is not received as recommended, patients will be analysed using the actual dose received. The dose-combination toxicity model is flexible and can include continuous doses of baclofen.
- **Population level summary measures:** The target safe baclofen dose from the dose-combination toxicity model which may or may not vary by methadone dose; if this varies by methadone dose the target safe baclofen doses will be accompanied by associated methadone dose ranges. Supporting population level summary measures: The mean probability of DLT with accompanying 95% Credible Intervals, probability of DLT being in the target range of 15%-25% and the probability of DLT being above the target range of 25% for 60mg of methadone in combination with 30mg of baclofen and for 120mg of methadone in combination with 90mg of baclofen from the dose-combination toxicity model. The frequency and percentage of patients experiencing a DLT, and the DLT parameters they occurred in, over all doses of baclofen, by baclofen dose cohort (as received) and by gender (across all methadone doses).

3.2 Secondary estimands

The secondary estimands for the secondary objectives described in Section 1.1.3 are all defined with the same population, treatment and handling of intercurrent event attributes as follows:

- **Population**: Opiate dependent individuals stably maintained on methadone meeting the inclusion/exclusion criteria who are able to receive baclofen.
- **Treatment condition**: Baclofen or placebo at the dose received. The allocated dose is specific to the entry cohort as recommended by the DSC based upon the dose-combination toxicity model

- **Handling of intercurrent events:**
    - Treatment not received - It is our intention to use the principal stratum strategy and target treatment effects only within the principal stratum (i.e. subset) of patients who would receive study treatment (baclofen or placebo at any dose). Thus only patients who receive treatment will be included in the analysis.

    - Dose received not as recommended – It is our intention to target the incidence of DLT for received doses (see treatment condition). Thus, if a dose is not received as recommended, patients will be analysed descriptively by closest dose received of 10mg 30mg 60mg and 90mg

There are 8 secondary estimands with continuous outcome variables, for which the outcome attribute is defined as follows:

- **Outcome**: NEWS2, recorded at time points: baseline, 60, 120, 180, 240 and 300 minutes
- **Outcome**: GCS, recorded at time points: baseline, 60, 120, 180, 240 and 300 minutes
- **Outcome**: QTc on ECG trace, recorded at time points: baseline, 120, and 300 minutes
- **Outcome**: SpO_2, recorded at time points: baseline, 15, 30, 60, 90, 120, 180, 240 and 300 minutes
- **Outcome**: CO_2, recorded at time points: baseline, 15, 30, 60, 90, 120, 180, 240 and 300 minutes
- **Outcome**: RR, recorded at time points: baseline, 15, 30, 60, 90, 120, 180, 240 and 300 minutes
- **Outcome**: TSHAS, recorded at time points: baseline, 60, 120, 180, 240 and 300 minutes
- **Outcome**: DEQ liking and wanting score, recorded at time points: baseline, 60, 120, 180, 240 and 300 minutes

There are 6 secondary estimands with count outcome variables which are defined as follows:

- **Outcome**: Incidence of a reduction in SPO_2 [≤91% for more than 30 seconds or >5% reduction in SpO_2 for more than 30 seconds] recorded at time points 60, 120, 180, 240 and 300 minutes
- **Outcome**: Incidence of reduced respiratory rate (≤8/min) recorded at time points 60, 120, 180, 240 and 300 minutes
- **Outcome**: Incidence of apnoea absence of inspiratory airflow for >30s combined with a sustained fall in SpO_2 at any point over follow-up], recorded at time points: 60, 120, 180, 240 and 300 minutes
- **Outcome**: SPO2 instances of <92% or of >5% reduction for more than 10 seconds, recorded at time points: 60, 120, 180, 240 and 300 minutes
- **Outcome**: CO_2 - instances of $tcCO_2$% exceeding a partial pressure CO_2 increase by 1kPa, recorded at time points: 60, 120, 180, 240 and 300 minutes
- **Outcome**: instances of absence of inspiratory airflow for more than 10 seconds or respiratory rate drops <9/min, recorded at time points: 60, 120, 180, 240 and 300 minutes

There is 1 secondary estimand with an ordinal outcome variables defined as follows:

- **Outcome**: Intervention level (0 to 4), recorded at time points: 15, 30, 60, 90, 120, 180, 240 and 300 minutes

The **population level summary** measures for the **continuous** outcomes are defined as:

- The mean, standard deviation, median and IQR of the [*outcome*] by treatment group (baclofen or placebo) at each time point. Additionally, at each time point, the mean, standard deviation, median and IQR of the [*outcome*] by baclofen dose group (as received) and gender.

The **population level summary** measures for the **count** outcomes are defined as:

- The frequency and percentage of individuals with *[outcome]* by treatment group (baclofen or placebo) at each time point. Additionally, at each time point, the frequency and percentage of individuals with *[outcome]* by baclofen dose group (as received) and gender. The mean total count of *[outcome]* per participant across all time points by treatment and additionally by baclofen dose group (as received) and gender.

The **population level summary** measures for the **ordinal** outcome is as follows:

- The frequency and proportion of individuals with Intervention level (3 or above) by treatment group (baclofen or placebo) at each time point. Additionally, at each time point, the number and proportion of individuals with Intervention level (3 or above) by baclofen dose group (as received) and gender. The mean standard deviation, median and IQR of *[outcome]* level by treatment arm at each time point. Additionally, at each time point, the mean, standard deviation, median and IQR of the [*outcome*] level by baclofen dose group (as received) and gender.

4. Data analysis plan

Dummy tables which will display the results of the following data analysis can be found in Appendix 1.

4.1 Recruitment and participant flow

A Consolidated Standards of Reporting Trials (CONSORT) flow chart will be prepared (figure A.1) summarising the number of eligible patients, the number of patients consenting to enter the trial, the number of patients randomised, the number of patients withdrawing (with reasons) and lost to follow-up, the numbers progressing through the trial and the numbers included in the analyses.(Schulz et al., 2010)

Numbers randomised will also be summarised by randomised treatment group (Table A.1).

4.2 Baseline characteristics

Baseline characteristics will be reported by treatment arm. This will include demographic characteristics, disease status and medical history. The full list of variables to be reported are listed in tables A.2, A.3 and A.4 for the FAS (see section 1.12.1). Continuous variables will be summarised as means and standard deviations (SD) and median and inter-quartile ranges. Categorical variables will be presented using frequencies and proportions (presented as percentages). These summaries will be based on observed values and the number of missing observations for each characteristic will be reported. No statistical significance testing will be performed.

4.3 Withdrawals, loss to follow-up and other missing data

The number withdrawing (including lost to follow-up) from assigned treatment will be tabulated by treatment arm (table A.5) and the reasons for withdrawal will be summarised by treatment arm (table A.6). The proportion of participants missing the primary outcome of DLT status will be summarised in each arm (table A.11).

4.4 Adherence

Adherence to assigned treatment will be calculated for individual participants as the proportion of dose received of the prescribed dose, and presented as the percentage received (table A.10). The average proportion of dose received (of prescribed) will be summarised as means and SDs and the frequency and proportion (presented as percentages) of participants who received dose as prescribed will be presented by treatment arm (table A.11).

4.5 Descriptive statistics for outcome measures

4.5.1 Primary outcome

Once the trial has been completed, summaries of the dose combination-toxicity model recommendations on the target safe baclofen dose over the course of the trial will be prepared. A table of estimated methadone ranges for each recommended baclofen dose as per model output for each cohort based on the observed data on DLT occurrences will be presented, along with corresponding probabilities that the risk of DLT for 60mg of

methadone in combination with 30mg of baclofen exceeds the target range (probability unsafe) and the probability that the risk of DLT for 120mg of methadone in combination with 90mg of baclofen does not exceed the target range (probability safe). See table A.7. The last row of this table will provide the target safe baclofen dose (primary outcome), which may or may not vary by methadone dose.

The individual participant recommended baclofen doses subject to their given methadone dose and the escalation constraints [(i) doses of baclofen cannot be skipped; (ii) the next group of patients cannot receive higher dose of baclofen than received by the individual(s) experiencing the DLT (if any) in the previous group] will also be presented for all trial participants, alongside the mean estimate of the DLT probability, the probability of DLT being in the target range of 15%-25% and the probability of DLT being above the target range of 25% for the recommended baclofen dose. See table A.8.

Using the final model update the mean estimate of the DLT probability with accompanying 95% Credible Intervals, the probability of DLT being in the target range of 15%-25% and the probability of DLT being above the target range of 25% for 60mg of methadone in combination with 30mg of baclofen and the probability of DLT not exceeding the target range for 120mg of methadone in combination with 90mg of baclofen will be presented. See table A.9.

Individual participant prescribed and received treatment doses alongside DLT status will be presented for all trial participants (table A.10).

The dose (de)escalation path over the course of the trial will be presented graphically as per template figure A.2. This will depict individual participant's baclofen dose by methadone range and DLT status.

Frequency and percentages of participants experiencing any DLT and the parameter in which the DLT occurred will be presented by treatment arm (table A.12). For baclofen participants frequency and percentage of participants experiencing any DLT and parameter in which the DLT occurred by baclofen dose (10mg, 30mg, 60mg and 90mg) and gender will be presented (table A.13 and table A.14 respectively).

4.5.2 Secondary outcomes

4.5.2.1 Components of DLT

Stimulus intervention level

The mean (SD) and median (IQR) of intervention level at each time point will be presented by treatment arm (table A.15). For baclofen participants the mean (SD) and median (IQR) of intervention level number at each time point by baclofen dose (10mg, 30mg, 60mg and 90mg) and gender will be summarised and presented (table A.16 and table A.17 respectively). The frequency and percentage of participants requiring intervention levels 3 or above at each time point will be presented by treatment arm (table A.15) and also by baclofen dose (table A.16) and gender (table A.17).

National Early Warning Score (NEWS2)

NEWS2 values will be summarised by treatment arm over time and presented as means (SD) and medians (IQR) (table A.18). For baclofen participants NEWS2 scores will be summarised by baclofen dose (10mg, 30mg, 60mg and 90mg) and gender (table A.20 and table A.22 respectively).

Individual participant trajectories of raw values over time, plotted by treatment arm (will include one plot per treatment arm) with patient identifiers clearly labelled on each trajectory will be presented (figure A.3). For baclofen participants individual participant trajectories of raw values over time by baclofen dose (will include one plot per baclofen dose) (figure A.4) and gender (separate plots for females and males will be presented) (figure A.5).

Summaries (mean (SD)) of NEWS2 scores over time across treatment arms will be plotted (will include one plot with distinct lines plotted for each treatment arm) (figure A.6). This plot will also be produced stratified by baclofen dose (placebo, 10mg, 30mg, 60mg and 90mg) (figure A.7) and gender (figure A.8).

Provided that data allows (i.e. adequate data is obtained on baclofen and placebo) an exploratory linear mixed effect model (model 1) will be used to estimate the mean difference in NEWS2 scores between intervention arm (baclofen) and placebo over follow-up time points. Participants will be included as a random intercept with fixed effects for time (60, 120, 180, 240, 300 minutes), treatment group (placebo or baclofen), time and treatment group interactions, methadone dose, methadone dose and treatment group interaction and baseline NEWS2 scores. The baseline adjusted treatment group differences with 95% confidence intervals for each time point will be presented (table A.19). No formal statistical testing will be conducted at this point. The model to be fitted to estimate the treatment effect at each time point will be:

Model 1

$$Y_{it} = \beta_0 + \beta_1 BAC_i + \beta_2 METH_i + \beta_3 NEWS_i^0 + \beta_4 time_{120} + \beta_5 time_{180} + \beta_6 time_{240} + \beta_7 time_{300} + \beta_8 BAC_i * time_{120} + \beta_9 BAC_i * time_{180} + \beta_{10} BAC_i * time_{240} + \beta_{11} BAC_i * time_{300} + \beta_{12} METH_i * BAC_i + b_{1,i} + e_{it}$$

for

t = 1 to 5 time points (60, 120, 180, 240 and 300 minutes), i = 1 up to 64 participants

BAC_i: dummy variable for treatment ($BAC_i = 0\ or\ 1$) for participant i

$METH_i$: methadone dose for participant i

$NEWS_i^0$: baseline NEWS value for participant i

$time_x$: dummy variable for time (= 0 or 1) at time point x minutes. 60 minutes is represented by $time_{120} = 0\ and\ time_{180} = 0\ and\ time_{240} = 0\ and\ time_{300} = 0$

$b_{1,i} \sim N(0, \sigma_{b1}^2)$, $e_{it} \sim N(0, \sigma_e^2)$

Where $b_{1,i}$ is the random intercept at the participant level. Each of $b_{1,i}$ and e_{it} are assumed to follow normal distributions. An unstructured covariance matrix will be used and the model will be fitted using restricted maximum likelihood (REML). This model makes assumptions about random effects distributions, correlation structures and residuals, which will all be investigated. If the model fails to converge, in the first instance we will remove the methadone by treatment group interaction, if convergence remains a problem we will remove the methadone covariate. In the final instance, if the model fails to converge the model estimates will not be presented.

If adequate data is obtained at multiple baclofen doses we will fit a separate linear mixed model (model 2) where the fixed treatment group covariate in model 1 is replaced with the continuous variable of baclofen dose (where placebo is included as 0). The baseline

adjusted treatment group differences with 95% confidence intervals for each time point will be presented (table A.21). The model to be fitted to estimate the treatment effect at each time point will be:

Model 2

$$Y_{it} = \beta_0 + \beta_1 BAC\_CTS_i + \beta_2 METH_i + \beta_3 NEWS_i^0 + \beta_4 time_{120} + \beta_5 time_{180} + \beta_6 time_{240} + \beta_7 time_{300} + \beta_8 BAC\_CTS_i * time_{120} + \beta_9 BAC\_CTS_i * time_{180} + \beta_{10} BAC\_CTS_i * time_{240} + \beta_{11} BAC\_CTS_i * time_{300} + \beta_{12} METH_i * BAC\_CTS_i + b_{1,i} + e_{it}$$

for

t = 1 to 5 time points (60, 120, 180, 240 and 300 minutes), i = 1 up to 64 participants

BAC_CTS_i: variable for baclofen ($BAC\_CTS_i = 0\ for\ placebo$) for participant i

$METH_i$: methadone dose for participant i

$NEWS_i^0$: baseline NEWS value for participant i

$time_x$: dummy variable for time (= 0 or 1) at time point x minutes. 60 minutes is represented by $time_{120} = 0\ and\ time_{180} = 0\ and\ time_{240} = 0\ and\ time_{300} = 0$

$b_{1,i} \sim N(0, \sigma_{b1}^2)$, $e_{it} \sim N(0, \sigma_e^2)$

If model results are unstable due to sparseness of data in the first instance we will remove the methadone by treatment group interaction, if convergence remains a problem we will remove the methadone covariate. In the final instance, if the model fails to converge we will remove the time by baclofen dose interaction and estimate the effect of baclofen dose over all time points.

Associations between the NEWS2 score and methadone dose at 120 minutes will be presented as correlations for each treatment arm using the Pearson's correlation if NEWS2 are approximately normally distributed (as assessed by graphical summaries) or the Spearman correlation if non-normal (table A.23) and presented graphically using a scatterplot (figure A.9). This may also be examined at the time point of the highest average NEWS2 score in the baclofen arm if appropriate.

The baseline adjusted treatment group difference by methadone dose (model 1 covariate - β_{12}) with 95% confidence interval will also be presented (table A.24).

Glasgow Coma Scale (GCS)

GCS scores will be summarised by treatment arm over time and presented as means (SD) and medians (IQR) (table A.25). For baclofen participants GCS scores will be summarised by baclofen dose (10mg, 30mg, 60mg and 90mg) and gender (table A.27 and table A.29 respectively).

Individual participant trajectories of raw GCS values over time, plotted by treatment arm (will include one plot per treatment arm) with patient identifiers clearly labelled on each trajectory will be presented (figure A.10). For baclofen participants individual participant trajectories of raw values over time by baclofen dose (will include one plot per baclofen dose) (figure A.11) and gender (separate plots for females and males will be presented) (figure A.12).

Summaries (mean (SD)) of GCS scores over time across treatment arms will be plotted (will include one plot with distinct lines plotted for each treatment arm) (figure A.13). This plot will also be produced stratified by baclofen dose (placebo, 10mg, 30mg, 60mg and 90mg) (figure A.14) and gender (figure A.15).

Provided that data allows (i.e. adequate data is obtained on baclofen and placebo) a linear mixed effect model (model 1 above) will be used to estimate the mean difference in GCS scores between intervention arm (baclofen) and placebo over follow-up time points. The model to be fitted to estimate the treatment effect at each time point will be as per model 1 described above but with outcome replaced with GCS score and baseline NEWS2 scores replaced with baseline GCS scores (table A.26).

If adequate data is obtained at multiple baclofen doses we will fit a separate linear mixed model where the fixed treatment group covariate in model 1 is replaced with the continuous variable of baclofen dose (where placebo is included as 0) as per model 2 above with GCS scores replacing NEWS2 scores (table A.28). If model results are unstable due to sparseness of data in the first instance we will remove the methadone by treatment group interaction, if convergence remains a problem we will remove the methadone covariate. In the final instance, if the model fails to converge we will remove the time by baclofen dose interaction and estimate the effect of baclofen dose over all time points.

Associations between the GCS scores and methadone dose at 120 minutes will presented as correlations for each treatment arm using the Pearson's correlation if GCS scores are approximately normally distributed (as assessed by graphical summaries) or the Spearman correlation if non-normal (table A.30) and presented graphically using a scatterplot (figure A.16). This may also be examined at the time point of the highest average GCS score in the baclofen arm if appropriate.

The baseline adjusted treatment group difference by methadone dose (model 1 covariate - β_{12}) with 95% confidence interval will also be presented (table A.31).

QTc on ECG trace

QTc scores will be summarised by treatment arm over time and presented as means (SD) and medians (IQR) (table A.32). For baclofen participants QTc scores will be summarised by baclofen dose (10mg, 30mg, 60mg and 90mg) and gender (table A.34 and table A.36 respectively).

Individual participant trajectories of QTc values over time, plotted by treatment arm (will include one plot per treatment arm) with patient identifiers clearly labelled on each trajectory will be presented (figure A.17). For baclofen participants individual participant trajectories of raw values over time by baclofen dose (will include one plot per baclofen dose) (figure A.18) and gender (separate plots for females and males will be presented) (figure A.19).

Summaries (mean (SD)) of QTc over time across treatment arms will be plotted (will include one plot with distinct lines plotted for each treatment arm) (figure A.20). This plot will also be produced stratified by baclofen dose (placebo, 10mg, 30mg, 60mg and 90mg) (figure A.21) and gender (figure A.22).

Provided that data allows (i.e. adequate data is obtained on baclofen and placebo) a linear mixed effect model (model 1) will be used to estimate the mean difference in QTc scores between intervention arm (baclofen) and placebo over follow-up time points. The model to be fitted to estimate the treatment effect at each time point will be as per model 1 described

above but with outcome replaced with QTc score, baseline NEWS2 scores replaced with baseline QTc scores and with only 120 and 300 minute time points (table A.33).

If adequate data is obtained at multiple baclofen doses we will fit a separate linear mixed model where the fixed treatment group covariate in model 1 is replaced with the continuous variable of baclofen dose (where placebo is included as 0) as per model 2 above with QTc scores replacing NEWS2 scores with only 120 and 300 minute time points (table A.35). If model results are unstable due to sparseness of data in the first instance we will remove the methadone by treatment group interaction, if convergence remains a problem we will remove the methadone covariate. In the final instance, if the model fails to converge we will remove the time by baclofen dose interaction and estimate the effect of baclofen dose over all time points.

Associations between the QTc scores and methadone dose at 120 minutes will presented as correlations for each treatment arm using the Pearson's correlation if QTc scores are approximately normally distributed (as assessed by graphical summaries) or the Spearman correlation if non-normal (table A.37) and presented graphically using a scatterplot (figure A.23). This may also be examined at the time point of the highest average QTc score in the baclofen arm if appropriate.

The baseline adjusted treatment group difference by methadone dose (model 1 covariate - β_{12}) with 95% confidence interval will also be presented (table A.38).

Measures of respiratory function (measured continuously)

Oxygen saturation (SpO_2), $tcCO_2$ and respiratory rates (RR) will be summarised by treatment arm over time and presented as means (SD) and medians (IQR) (tables A.39, A.46 and A.353). For baclofen participants SpO_2, $tcCO_2$ and RR will be summarised by baclofen dose (10mg, 30mg, 60mg and 90mg) (tables A.41, A.48 and A.55 respectively) and gender (tables A.43, A.50 and A.57 respectively).

Individual participant trajectories of SpO_2, $tcCO_2$ and RR values over time, plotted by treatment arm (will include one plot per treatment arm) with patient identifiers clearly labelled on each trajectory will be presented (figure A.24, A.317 and A.38). For baclofen participants individual participant trajectories of SpO_2, tcO_2 and RR values over time by baclofen dose (will include one plot per baclofen dose) (figure A.25, A.32 and A.39) and gender (separate plots for females and males will be presented) (figure A.26, A.33 and A.40).

Summaries (mean (SD)) of SpO_2, $tcCO_2$ and RR values over time across treatment arms will be plotted (will include one plot with distinct lines plotted for each treatment arm) (figure A.27, A.34 and A.41). This plot will also be produced stratified by baclofen dose (placebo, 10mg, 30mg, 60mg and 90mg) (figure A.28, A.35 and A.42) and gender (figure A.29, A.36 and A.43).

Provided that data allows (i.e. adequate data is obtained on baclofen and placebo) separate linear mixed effect models (model 1) will be used to estimate the mean difference in each of SpO_2, $tcCO_2$ and RR values between intervention arm (baclofen) and placebo over follow-up time points. The models to be fitted to estimate the treatment effect at each time point will be as per model 1 described above but with outcome replaced with one of $SpO_{2,}$ $tcCO_2$ or RR values score, baseline NEWS2 scores replaced with one of baseline SpO_2, $tcCO_2$ or RR values and additional time points of 15 minutes, 30 minutes and 90 minutes (table A.40, A.47 and A.54).

If adequate data is obtained at multiple baclofen doses we will fit a separate linear mixed model where the fixed treatment group covariate in model 1 is replaced with the continuous variable of baclofen dose (where placebo is included as) as per model 2 above with one of SpO_2, $tcCO_2$ and RR values replacing NEWS2 scores and additional time points of 15 minutes, 30 minutes and 90 minutes (table A.42, A.49, and A.56). If model results are unstable due to sparseness of data in the first instance we will remove the methadone by treatment group interaction, if convergence remains a problem we will remove the methadone covariate. In the final instance, if the model fails to converge we will remove the time by baclofen dose interaction and estimate the effect of baclofen dose over all time points.

Associations between the SpO_2, $tcCO_2$ and RR values and methadone dose at 120 minutes will presented as correlations for each treatment arm using the Pearson's correlation if scores are approximately normally distributed (as assessed by graphical summaries) or the Spearman correlation if non-normal (table A.44, A.51 and A.58) and presented graphically using a scatterplot (figure A.30, A.37 and A.44). This may also be examined at the time point of the highest average SpO_2, $tcCO_2$ and RR values in the baclofen arm if appropriate.

The baseline adjusted treatment group difference by methadone dose (model 1 covariate - β_{12}) with 95% confidence interval will also be presented (tables A.45, A.52 and A5.9).

The mean (SD) number of events per participant and frequency and percentages of participants experiencing any of the following respiratory changes at least once including, a reduction in SPO_2 [≤91% for more than 30 seconds or >5% reduction in SpO_2 for more than 30 seconds], reduced respiratory rate (≤8/min) or apnoea [absence of inspiratory airflow for >30s combined with a sustained fall in SpO_2 at any point over follow-up] will be presented by treatment arm (table A.60). For baclofen participants the mean (SD) number of events per participant and frequency and percentage of participants experiencing any of these respiratory changes at least once by baclofen dose (10mg, 30mg, 60mg and 90mg) and gender will be presented (table A.61 and table A.62 respectively).

4.5.2.2 Respiratory measures for signs of sub-threshold respiratory depression

SpO2

The mean (SD) number of events per participant and frequency and percentages of participants experiencing instances of SpO_2 <92% or of >5% reduction for more than 10 seconds at any point over follow-up will be presented by treatment arm (table A.63). For baclofen participants the mean (SD) number of events per participant and the frequency and percentage of participants experiencing instances of SpO_2 <92% or of >5% reduction for more than 10 seconds at any point over follow-up by baclofen dose (10mg, 30mg, 60mg and 90mg) and gender will be presented (table A.64 and table A.65 respectively).

CO_2

The mean (SD) number of events per participant and frequency and percentages of participants experiencing instances of $tcCO_2$% exceeding a partial pressure CO_2 increase by 1kPa at any point over follow-up will be presented by treatment arm (table A.63). For baclofen participants the mean (SD) number of events per participant and the frequency and percentage of participants experiencing instances of $tcCO_2$% exceeding a partial pressure CO_2 increase by 1kPa at any point over follow-up by baclofen dose (10mg, 30mg, 60mg and 90mg) and gender will be presented (table A.64 and table A.65 respectively).

Respiratory rate
The mean (SD) number of events per participant and frequency and percentages of participants experiencing instances of absence of inspiratory airflow for more than 10 seconds or respiratory rate drops <9/min at any point over follow-up will be presented by treatment arm (table A.63). For baclofen participants the mean (SD) number of events per participant and the frequency and percentage of participants experiencing instances of absence of inspiratory airflow for more than 10 seconds or respiratory rate drops <9/min at any point over follow-up by baclofen dose (10mg, 30mg, 60mg and 90mg) and gender will be presented (table A.64 and table A.65 respectively).

4.5.2.3 Sedation measures

T-SHAS score
Total SHAS score will be summarised by treatment arm over time and presented as means (SD) and medians (IQR) (table A.66). For baclofen participants total SHAS will be summarised by baclofen dose (10mg, 30mg, 60mg and 90mg) and gender (table A.68 and table A.70 respectively).

Individual participant trajectories of total SHAS scores over time, plotted by treatment arm (will include one plot per treatment arm) with patient identifiers clearly labelled on each trajectory will be presented (figure A.45). For baclofen participants individual participant trajectories of total SHAS scores over time by baclofen dose (will include one plot per baclofen dose) and gender (separate plots for females and males will be presented) (figures A.46 and A.47).

Summaries (mean (SD)) of total SHAS scores over time across treatment arms will be plotted (will include one plot with distinct lines plotted for each treatment arm) (figure A.48). This plot will also be produced stratified by baclofen dose (10mg, 30mg, 60mg and 90mg) (figure A.49) and gender (figure A.50).

Provided that data allows (i.e. adequate data is obtained on baclofen and placebo) a linear mixed effect model (model 1) will be used to estimate the mean difference in total SHAS scores between intervention arm (baclofen) and placebo over follow-up time points. The model to be fitted to estimate the treatment effect at each time point will be as per model 1 described above but with outcome replaced with total SHAS score and baseline NEWS2 scores replaced with baseline total SHAS scores (table A.67).

If adequate data is obtained at multiple baclofen doses we will fit a separate linear mixed model where the fixed treatment group covariate in model 1 is replaced with the continuous variable of baclofen dose (where placebo is included as 0) as per model 2 above with total SHAS scores replacing NEWS2 scores (table A.69). If model results are unstable due to sparseness of data in the first instance we will remove the methadone by treatment group interaction, if convergence remains a problem we will remove the methadone covariate. In the final instance, if the model fails to converge we will remove the time by baclofen dose interaction and estimate the effect of baclofen dose over all time points.

Associations between the total SHAS scores and methadone dose at 120 minutes will presented as correlations for each treatment arm using the Pearson's correlation if total SHAS scores are approximately normally distributed (as assessed by graphical summaries) or the Spearman correlation if non-normal (table A.71) and presented graphically using a

scatterplot (figure A.51). This may also be examined at the time point of the highest average SHAS score in the baclofen arm if appropriate.

The baseline adjusted treatment group difference by methadone dose (model 1 covariate - β_{12}) with 95% confidence interval will be presented (table A.72).

4.5.2.4 Symptom measures

Drug effects questionnaire

Scores on DEQ items relating to 'liking' and 'wanting more' will be summarised by treatment arm over time and presented as means (SD) and medians (IQR) (table A.73 and table A.46). For baclofen participants 'liking' and 'wanting more' scores will be summarised by baclofen dose (10mg, 30mg, 60mg and 90mg) (table A.77 and table A.78 respectively) and gender (table A.81 and table A.82 respectively).

Individual participant trajectories of 'liking' and 'wanting more' scores over time, plotted by treatment arm (will include one plot per treatment arm) with patient identifiers clearly labelled on each trajectory will be presented (figures A.52 and A.53). For baclofen participants individual participant trajectories of 'liking' and 'wanting more' scores over time by baclofen dose (will include one plot per baclofen dose) (figures A.54 and A.55) and gender (separate plots for females and males will be presented) (figures A.56 and A.57).

Summaries (mean (SD)) of 'liking' and 'wanting more' scores over time across treatment arms will be plotted (will include one plot with distinct lines plotted for each treatment arm) (figures A.58 and A.59). This plot will also be produced stratified by baclofen dose (10mg, 30mg, 60mg and 90mg) (figures A.60 and A.61) and gender (figures A.62 and A.63).

Provided that data allows (i.e. adequate data is obtained on baclofen and placebo) separate linear mixed effect models (model 1) will be used to estimate the mean difference in DEQ items relating to 'liking' and 'wanting more' between intervention arm (baclofen) and placebo over follow-up time points. The model to be fitted to estimate the treatment effect at each time point will be as per model 1 described above but with outcome replaced with one of DEQ items relating to 'liking' and 'wanting more' and baseline NEWS2 scores replaced with corresponding baseline DEQ items relating to 'liking' and 'wanting more' (table A.75 and A.76).

If adequate data is obtained at multiple baclofen doses we will fit separate linear mixed models for both DEQ items relating to 'liking' and 'wanting more' where the fixed treatment group covariate in model 1 is replaced with the continuous variable of baclofen dose (where placebo is included as 0) as per model 2 above with DEQ items relating to 'liking' and 'wanting more' replacing NEWS2 scores (table A.79 and A.80). If model results are unstable due to sparseness of data in the first instance we will remove the methadone by treatment group interaction, if convergence remains a problem we will remove the methadone covariate. In the final instance, if the model fails to converge we will remove the time by baclofen dose interaction and estimate the effect of baclofen dose over all time points.

Associations between each of the DEQ items relating to 'liking' and 'wanting more' and methadone dose at 120 minutes will presented as correlations for each treatment arm using the Pearson's correlation if DEQ scores are approximately normally distributed (as assessed by graphical summaries) or the Spearman correlation if non-normal (table A.83 and A.84) and presented graphically using a scatterplot (figure A.64 and A.65). This may also be examined at the time point of the highest average DEQ score in the baclofen arm if appropriate.

The baseline adjusted treatment group difference by methadone dose (model 1 covariate - β_{12}) with 95% confidence interval will also be presented (tables A.85 and A.86).

4.6 Adverse events and serious adverse events

Adverse events will be tabulated by type (e.g. adverse event, adverse reaction, unexpected adverse reaction, serious adverse event, serious adverse reaction, unexpected serious adverse reaction) and by treatment arm (table A.87).

Adverse events coded as per the MedDra dictionary will be summarised at the preferred term level. Events will be tabulated by treatment arm including information on the number with at least one event and the number of events to account for recurrent events (table A.88). This will be repeated by treatment arm and severity grade and will include information on the number with at least one event by maximum grade and the number of events to account for recurrent events. This will be presented in both a table (table A.89) and graphically using a stacked bar chart (figure A.66).

In addition, risk differences between those experiencing at least one event for each event at preferred time between treatment arms with corresponding 95% confidence intervals (CIs) will be calculated and incident rate ratios to account for recurrent events with 95% CIs using a suitable model e.g. a negative binomial or zero inflated Poisson model as appropriate. These results will be presented in both a table (table A.90) and graphically using a dot plot (figure A.67).

Raw data for each SAE will be listed with information on event type, timing duration and outcome (table A.56).

4.7 Missing data

Every effort will be made to obtain all follow-up data for all participants. We anticipate minimal missing data as the evaluation window for primary and secondary outcome measures will begin at dosing and end at 5 hours post-dose, with the exception of the ‘intervention level’ (as defined in the DLT definition) which will begin at dosing and continue until the last follow-up phone call. This call will be conducted the following day, and the window will be extended if the participant is experiencing sedation or other symptoms.

Participants who withdraw from the study before receiving study treatment will be replaced if necessary. In the unlikely event that a participant decided to withdraw after dosing but prior to the end of the 5 hour evaluation window, this participant could be counted as an evaluable patient with no DLTs provided they had completed the 2-3h time point and that this self-discharge had occurred within a clinical picture of stable or normalising observations. Attempts to complete the follow-up phone call would be made to confirm absence of DLT as defined by ‘intervention level’. In the event of an inability to acquire sufficient data, as defined above, the decision on whether to include an individual’s data in the primary (DLT) or secondary analysis, or whether that individual would be replaced, would be made on a case-by-case basis.

No imputation will be performed for any missing data for primary and secondary analyses. All data summaries will be based on observed data only and will assume any data is missing completely at random (MCAR). Linear mixed model analyses employ maximum likelihood estimation and thus are efficient for handing missing outcome data under a missing at random (MAR) assumption.

In the case of missing data on the primary outcome (DLT) for any participant who withdraws after dosing, but before adequate data is obtained, sensitivity analysis will be conducted for the primary analysis to explore the robustness to alternative missing data assumptions as follows:

(i) Participants who receive treatment with missing data included in the sensitivity analysis and assumed to have experienced a DLT

(ii) Participants who receive treatment with missing data included in the sensitivity analysis and assumed to not have experienced a DLT

The dose combination-toxicity model will be updated in the sensitivity analysis and we will report the estimated methadone ranges for each baclofen dose as per model output based on the observed and imputed data, along with corresponding probabilities that the risk of DLT for 60mg of methadone in combination with 30mg of baclofen exceeds the target range is 25% or more (probability unsafe) and the probability that the risk of DLT for 120mg of methadone in combination with 90mg of baclofen does not exceed the target range is 92.5% or more (probability safe) (repeat last row of table A.7).

5. Software

The Bivariate Bayesian Logistic Regression Adaptive model will be updated for the primary objective using R software The code for implementation of the primary analysis is adapted from that found in Mozgunov et al. 2021. All other analysis will be undertaken across either R or Stata.

6. Appendix 1: Template tables and figures

Figure A.1: Participant flow

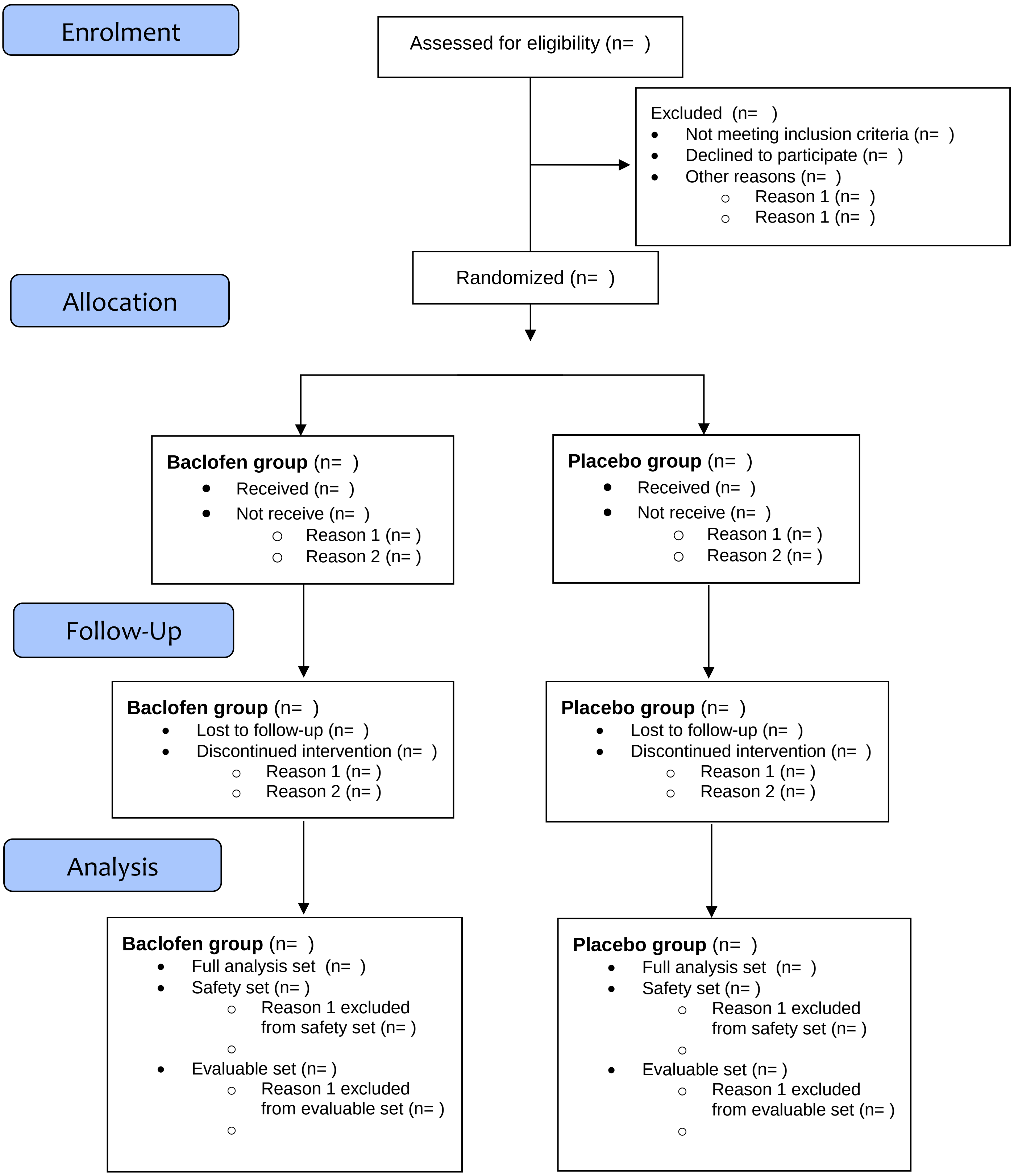

Table A.1: Randomisation by treatment arm

	**Baclofen N (%)**	**Placebo N (%)**	**Total N**
Number randomised to date (dd/mm/yyyy)			

Table A.2: Baseline characteristics by treatment arm

Characteristic, n (%)	Baclofen (N=xxx)	Placebo (N=xxx)	Total (N=xxx)
Age (years)			
Nmissing			
Mean (SD)			
Median (IQR)			
Sex			
Nmissing			
Male			
Female			
Ethnicity			
Nmissing			
White			
Black, African, Caribbean or Black British			
Mixed or Multiple ethnic groups			
Asian or Asian British			
Other			
Education (years)			
Nmissing			
Mean (SD)			
Median (IQR)			
Height (metres)			
Nmissing			
Mean (SD)			
Median (IQR)			
Weight (kg)			
Nmissing			
Mean (SD)			
Median (IQR)			
BMI (kg/m2)			
Nmissing			
Mean (SD)			
Median (IQR)			
Temperature (°C)			
Nmissing			
Mean (SD)			
Median (IQR)			
Pulse (bpm)			
Nmissing			
Mean (SD)			
Median (IQR)			
Systolic BP			
Nmissing			
Mean (SD)			
Median (IQR)			
Diastolic BP			
Nmissing			

Mean (SD)	
Median (IQR)	
BP position (%)	
Nmissing	
Supine	
Standing	
Sitting	
Oxygen saturation (%)	
Nmissing	
Mean (SD)	
Median (IQR)	
Drugs of abuse	
Nmissing	
COC	
AMP	
THC	
MTD	
OPI	
BZD	
ECG	
Nmissing	
Normal	
Abnormal NCS	
Abnormal CS	
QTc interval	
Nmissing	
Mean (SD)	
Median (IQR)	
QTc interval	
Nmissing	
Normal	
Abnormal NCS	
Abnormal CS	
BDI	
Nmissing	
Mean (SD)	
Median (IQR)	
STAI	
Nmissing	
Mean (SD)	
Median (IQR)	
VAS anxiety	
Nmissing	
Mean (SD)	
Median (IQR)	
VAS craving	
Nmissing	
Mean (SD)	
Median (IQR)	
PSQI	

Nmissing	
Mean (SD)	
Median (IQR)	
ESS	
Nmissing	
Mean (SD)	
Median (IQR)	
RLS	
Nmissing	
Mean (SD)	
Median (IQR)	
FTND	
Nmissing	
Mean (SD)	
Median (IQR)	
AUDIT	
Nmissing	
Mean (SD)	
Median (IQR)	
SDS	
Nmissing	
Mean (SD)	
Median (IQR)	
OCDUS-H	
Nmissing	
Mean (SD)	
Median (IQR)	
Methadone dose at screening (taken from drug and alcohol history)	
Nmissing	
Mean (SD)	
Median (IQR)	

Table A.3: Drug and alcohol diagnoses by treatment arm

Characteristic, n (%)	Baclofen (N=xxx)	Placebo (N=xxx)	Total (N=xxx)
Alcohol status			
Nmissing			
Current regular drinker			
Current occasional drinker			
Ex-dependent drinker			
Ex-regular drinker			
Never			
Smoking status			
Nmissing			
Current regular smoker			
Current occasional smoker			
Ex-dependent smoker			
Ex-occasional smoker			
Never			

Table A.4: Other medical diagnoses by treatment arm

Characteristic, n (%)	Baclofen (N=xxx)	Placebo (N=xxx)	Total (N=xxx)
Cardiovascular diagnosis			
Yes			
Respiratory diagnosis			
Yes			
Digestive diagnosis			
Yes			
Urinary/renal diagnosis			
Yes			
Reproductive diagnosis			
Yes			
Musculoskeletal diagnosis			
Yes			
Nervous/sensory diagnosis			
Yes			
Endocrine diagnosis			
Yes			
Integumentary diagnosis			
Yes			
Circulatory/lymphatic diagnosis			
Yes			
Immune diagnosis			
Yes			
MINI total score			
Nmissing			
Mean (SD)			
Median (IQR)			
MINI question 4			
Nmissing			
Yes			
Risk factors for COPD			
Nmissing			
Yes			
COPD history			
Nmissing			
History of persistent cough or breathlessness at rest			
Previous verified diagnosis of chronic obstructive pulmonary disease (COPD)			
Hospital admission due to COPD, non-invasive ventilation for type 2 respiratory failure			
Use of inhalers for respiratory compromise			

Ventilation due to COVID-19
History of inhalation/smoking of substances e.g. heroin, tobacco, crack cocaine
Other

Table A.5: Withdrawals (including lost to follow-up) by treatment arm

Withdrawal:	**Baclofen N (%)**	**Placebo N (%)**	**Total N**
Yes			

Table A.6: Reasons for withdrawal by treatment group

Reason, n (%)	**Baclofen N (%)**	**Placebo N (%)**	**Total N**
Completed study			
Lost to follow-up			
Patient withdrew consent from further administration of study medication only but has consented to future data collection			
Patient withdrew consent from further administration of study medication and all future data collection			
Investigator Decision			
Serious/Adverse Event			
Pregnancy			
Death			
Other			

Table A.7: Estimated methadone ranges for each recommended baclofen dose as per model output*

#	Methadone doses to date (mg)			Baclofen doses to date (mg)			DLTs to date[1]			Probability unsafe	Probability safe	Min meth **10mgBAC**	Max meth **10mgBAC**	Min meth **30mgBAC**	Max meth **30mgBAC**	Min meth **60mgBAC**	Max meth **60mgBAC**	Min meth **90mgBAC**	Max meth **90mgBAC**
1	xxx	xxx	xxx	xx	xx	xx	x	x	x	x.xxx	x.xxx	xxx	xxx	xxx	xxx	xxx	xxx	xxx	xxx
2	xxx	xxx	xxx	xx	xx	xx	x	x	x	x.xxx	x.xxx	xxx	xxx	xxx	xxx	xxx	xxx	xxx	xxx
3																			
4																			
5																			
..																			

# - Baclofen group; DLT – dose limiting toxicity
[1] DLT= 0 indicates no DLT; DLT=1 indicates DLT

*Warning these ranges do not take into account non-skipping constraints and are pure model estimates. Dose recommendations subject to escalation constraints are given below in table A.6.

Table A.8: Participants recommended Baclofen doses subject to escalation constraints*

Participant ID	Treatment arm (Baclofen or placebo)	Methadone (mg)	**Recommended baclofen (mg)**	Mean estimate of the DLT probability	Probability of DLT		Probability of DLT being in the target (P in 15%, 25%)	Probability of DLT being above the target (P>25%)
					Lower bound of 95% Credible Interval	Upper bound of 95% Credible Interval		
001		xxx	xx	x.xxx	x.xxx	x.xxx	x.xxx	x.xxx
002		xxx	xx	x.xxx	x.xxx	x.xxx	x.xxx	x.xxx
003		xxx	xx	x.xxx	x.xxx	x.xxx	x.xxx	x.xxx
004		xxx	xx	x.xxx	x.xxx	x.xxx	x.xxx	x.xxx
005								
…								

*Escalation constraints: (i) doses of baclofen cannot be skipped; (ii) the next group of patients cannot receive higher dose of baclofen than received by the individual(s) experiencing the DLT (if any) in the previous group

NOTE: If patient allocated placebo then the recommended baclofen dose would be given as placebo matching the dosage

Table A.9: DLT probabilities for hypothetical scenarios

**Methadone (mg)**	**Baclofen (mg)**	Mean estimate of the DLT probability	Probability of DLT		Probability of DLT being in the target (P in 15%, 25%)	Probability of DLT being above the target (P>25%)
			Lower bound of 95% Credible Interval	Upper bound of 95% Credible Interval		
**60**	**30**	x.xxx	x.xxx	x.xxx	x.xxx	x.xxx
**120**	**90**	x.xxx	x.xxx	x.xxx	x.xxx	x.xxx

Table A.10: Participants prescribed and received doses as per DSC recommendations and DLT status

Participant ID	Treatment arm (Baclofen or placebo)	Methadone (mg)	**Prescribed baclofen dose (mg)**	Received baclofen dose (mg)	Percentage received of prescribed (%)	DLT[1] (0 or 1)
001		xxx	xx	xx		
002		xxx	xx	xx		
003		xxx	xx	xx		
004		xxx	xx	xx		
005						
…						

*Escalation constraints: (i) doses of baclofen cannot be skipped; (ii) the next group of patients cannot receive higher dose of baclofen than received by the individual(s) experiencing the DLT (if any) in the previous group
[1] DLT= 0 indicates no DLT; DLT=1 indicates DLT

NOTE: If patient allocated placebo then the recommended baclofen dose should be given as placebo matching the dosage

Table A.11: Adherence to prescribed dose

Characteristic		Baclofen (N=xxx)	Placebo (N=xxx)	Total (N=xxx)
Average proportion of dose received (of prescribed)				
	Nmissing			
	Mean (SD)			
Received dose as prescribed				
	Nmissing			
	Yes			

Figure A.2: Baclofen escalation/de-escalation path over the course of the trial

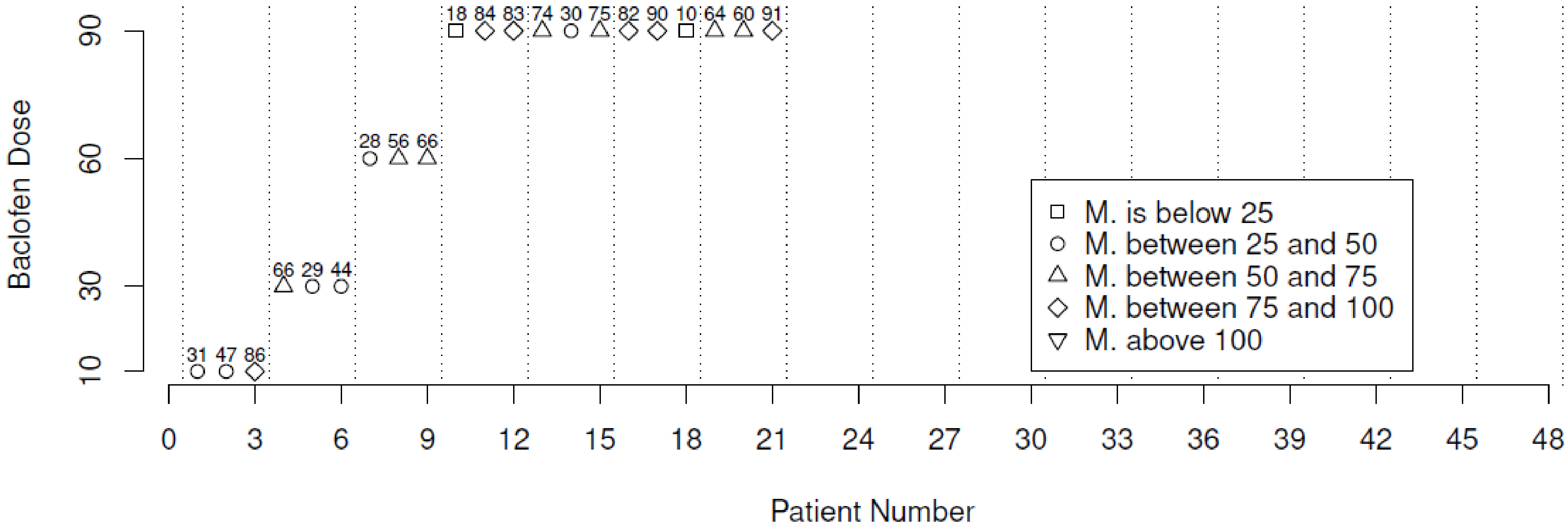

Table A.12: DLT status by baclofen dose

Characteristic, n (%)		Baclofen (N=xxx)	Placebo (N=xxx)	Total (N=xxx)
DLT status				
	Nmissing			
	DLT - yes			
DLT occurred in:				
	Situation requiring stimulus intervention ≥4			
	NEWS2 >4 or score of 3 in any parameter			
	GCS score <12			
	Persistent QTc prolongation			
	Measures of respiratory function with a persistent change			

Table A.13: DLT status by baclofen dose

		Baclofen dose (N=xxx)			
Characteristic, n (%)		10mg (N=xxx)	30mg (N=xxx)	60mg (N=xxx)	90mg (N=xxx)
DLT status					
	Nmissing				
	DLT - yes				
DLT occurred in:					
	Situation requiring stimulus intervention ≥4				
	NEWS2 >4 or score of 3 in any parameter				
	GCS score <12				
	Persistent QTc prolongation				
	Measures of respiratory function with a persistent change				

Table A.14: DLT status by gender for baclofen participants

		Gender (N=xxx)	
Characteristic, n (%)		Male (N=xxx)	Female (N=xxx)
DLT status			
	Nmissing		
	DLT - yes		
DLT occurred in:			
	Situation requiring stimulus intervention ≥4		
	NEWS2 >4 or score of 3 in any parameter		
	GCS score <12		
	Persistent QTc prolongation		
	Measures of respiratory function with a persistent change		

Table A.15: Stimulus intervention level by treatment arm

Time		Baclofen	Placebo	Total
Baseline				
	N (Nmissing)			
	Situation requiring stimulus intervention ≥3, n (%)			
	Mean (SD)			
	Median (IQR)			
15 mins				
	N (Nmissing)			
	Situation requiring stimulus intervention ≥3, n (%)			
	Mean (SD)			
	Median (IQR)			
30 mins				
	N (Nmissing)			
	Situation requiring stimulus intervention ≥3, n (%)			
	Mean (SD)			
	Median (IQR)			
60 mins				
	N (Nmissing)			
	Situation requiring stimulus intervention ≥3, n (%)			
	Mean (SD)			
	Median (IQR)			
90 mins				
	N (Nmissing)			
	Situation requiring stimulus intervention ≥3, n (%)			
	Mean (SD)			
	Median (IQR)			
120 mins				
	N (Nmissing)			

	Situation requiring stimulus intervention ≥3, n (%)			
	Mean (SD)			
	Median (IQR)			
180 mins				
	N (Nmissing)			
	Situation requiring stimulus intervention ≥3, n (%)			
	Mean (SD)			
	Median (IQR)			
240 mins				
	N (Nmissing)			
	Situation requiring stimulus intervention ≥3, n (%)			
	Mean (SD)			
	Median (IQR)			
300 mins				
	N (Nmissing)			
	Situation requiring stimulus intervention ≥3, n (%)			
	Mean (SD)			
	Median (IQR)			

Table A.16: Stimulus intervention level by baclofen dose

		Baclofen dose (N=xxx)			
Time		**10mg (N=xxx)**	**30mg (N=xxx)**	**60mg (N=xxx)**	**90mg (N=xxx)**
Baseline					
	N (Nmissing)				
	Situation requiring stimulus intervention ≥3, n (%)				
	Mean (SD)				
	Median (IQR)				
15 mins					
	N (Nmissing)				
	Situation requiring stimulus intervention ≥3, n (%)				
	Mean (SD)				
	Median (IQR)				
30 mins					
	N (Nmissing)				
	Situation requiring stimulus intervention ≥3, n (%)				
	Mean (SD)				
	Median (IQR)				
60 mins					
	N (Nmissing)				
	Situation requiring stimulus intervention ≥3, n (%)				
	Mean (SD)				
	Median (IQR)				
90 mins					
	N (Nmissing)				
	Situation requiring stimulus intervention ≥3, n (%)				
	Mean (SD)				
	Median (IQR)				
120 mins					

	N (Nmissing)				
	Situation requiring stimulus intervention ≥3, n (%)				
	Mean (SD)				
	Median (IQR)				
180 mins					
	N (Nmissing)				
	Situation requiring stimulus intervention ≥3, n (%)				
	Mean (SD)				
	Median (IQR)				
240 mins					
	N (Nmissing)				
	Situation requiring stimulus intervention ≥3, n (%)				
	Mean (SD)				
	Median (IQR)				
300 mins					
	N (Nmissing)				
	Situation requiring stimulus intervention ≥3, n (%)				
	Mean (SD)				
	Median (IQR)				

Table A.17: Stimulus intervention level by gender

		Gender (N=xxx)	
Time		**Male (N=xxx)**	**Male (N=xxx)**
Baseline			
	N (Nmissing)		
	Situation requiring stimulus intervention ≥3, n (%)		
	Mean (SD)		
	Median (IQR)		
15 mins			
	N (Nmissing)		
	Situation requiring stimulus intervention ≥3, n (%)		
	Mean (SD)		
	Median (IQR)		
30 mins			
	N (Nmissing)		
	Situation requiring stimulus intervention ≥3, n (%)		
	Mean (SD)		
	Median (IQR)		
60 mins			
	N (Nmissing)		
	Situation requiring stimulus intervention ≥3, n (%)		
	Mean (SD)		
	Median (IQR)		
90 mins			
	N (Nmissing)		
	Situation requiring stimulus intervention ≥3, n (%)		
	Mean (SD)		
	Median (IQR)		
120 mins			

	N (Nmissing)		
	Situation requiring stimulus intervention ≥3, n (%)		
	Mean (SD)		
	Median (IQR)		
180 mins			
	N (Nmissing)		
	Situation requiring stimulus intervention ≥3, n (%)		
	Mean (SD)		
	Median (IQR)		
240 mins			
	N (Nmissing)		
	Situation requiring stimulus intervention ≥3, n (%)		
	Mean (SD)		
	Median (IQR)		
300 mins			
	N (Nmissing)		
	Situation requiring stimulus intervention ≥3, n (%)		
	Mean (SD)		
	Median (IQR)		

Table A.18: NEWS2 score summaries by treatment arm

Time		**Baclofen**	**Placebo**	**Total**
Baseline				
	N (Nmissing)			
	Mean (SD)			
	Median (IQR)			
60 mins				
	N (Nmissing)			
	Mean (SD)			
	Median (IQR)			
120 mins				
	N (Nmissing)			
	Mean (SD)			
	Median (IQR)			
180 mins				
	N (Nmissing)			
	Mean (SD)			
	Median (IQR)			
240 mins				
	N (Nmissing)			
	Mean (SD)			
	Median (IQR)			
300 mins				
	N (Nmissing)			
	Mean (SD)			
	Median (IQR)			

Table A.19: Treatment effect estimates for NEWS2 scores at each time-point from the linear mixed effects model (model 1)

		**Baclofen versus placebo N=xxx**	
NEWS2		Adjusted mean difference*	95% CIs
	60 minutes	x.xx	x.xx, x.xx
	120 minutes	x.xx	x.xx, x.xx
	180 minutes	x.xx	x.xx, x.xx
	240 minutes	x.xx	x.xx, x.xx
	300 minutes	x.xx	x.xx, x.xx

*adjusted for baseline NEWS2 scores, methadone dose and methadone dose by treatment group (baclofen vs placebo) interaction

Table A.20: NEWS2 score summaries by baclofen dose

		Baclofen dose			
		**10mg**	**30mg**	**60mg**	**90mg**
Baseline					
	N (Nmissing)				
	Mean (SD)				
	Median (IQR)				
60 mins					
	N (Nmissing)				
	Mean (SD)				
	Median (IQR)				
120 mins					
	N (Nmissing)				
	Mean (SD)				
	Median (IQR)				
180 mins					
	N (Nmissing)				
	Mean (SD)				
	Median (IQR)				
240 mins					
	N (Nmissing)				
	Mean (SD)				
	Median (IQR)				
300 mins					
	N (Nmissing)				
	Mean (SD)				
	Median (IQR)				

Table A.21: Treatment effect estimates for NEWS2 scores at each time-point from the linear mixed effects model (model 2)

		**Baclofen dose (mg)<br>N=xxx**	
NEWS2		Adjusted mean difference*	95% CIs
	60 minutes	x.xx	(x.xx, x.xx)
	120 minutes	x.xx	(x.xx, x.xx)
	180 minutes	x.xx	(x.xx, x.xx)
	240 minutes	x.xx	(x.xx, x.xx)
	300 minutes	x.xx	(x.xx, x.xx)

*adjusted for baseline NEWS2 scores, methadone dose and methadone dose by baclofen dose (mg) interaction

Table A.22: NEWS2 score summaries by gender

		Gender	
		**Male**	**Female**
Baseline			
	N (Nmissing)		
	Mean (SD)		
	Median (IQR)		
60 mins			
	N (Nmissing)		
	Mean (SD)		
	Median (IQR)		
120 mins			
	N (Nmissing)		
	Mean (SD)		
	Median (IQR)		
180 mins			
	N (Nmissing)		
	Mean (SD)		
	Median (IQR)		
240 mins			
	N (Nmissing)		
	Mean (SD)		
	Median (IQR)		
300 mins			
	N (Nmissing)		
	Mean (SD)		
	Median (IQR)		

Table A.23: Correlation between NEWS2 scores and methadone dose by treatment arm

Table A.24: Estimates of methadone effect on NEWS2 scores between treatment arms from the linear mixed effects model (model 1)

		**Baclofen versus placebo N=xxx**	
NEWS2		Adjusted mean difference*	95% CIs
		x.xx	x.xx, x.xx

*adjusted for baseline NEWS2 scores

Figure A.3 Individual participant trajectories of NEWS2 scores over time by treatment arm

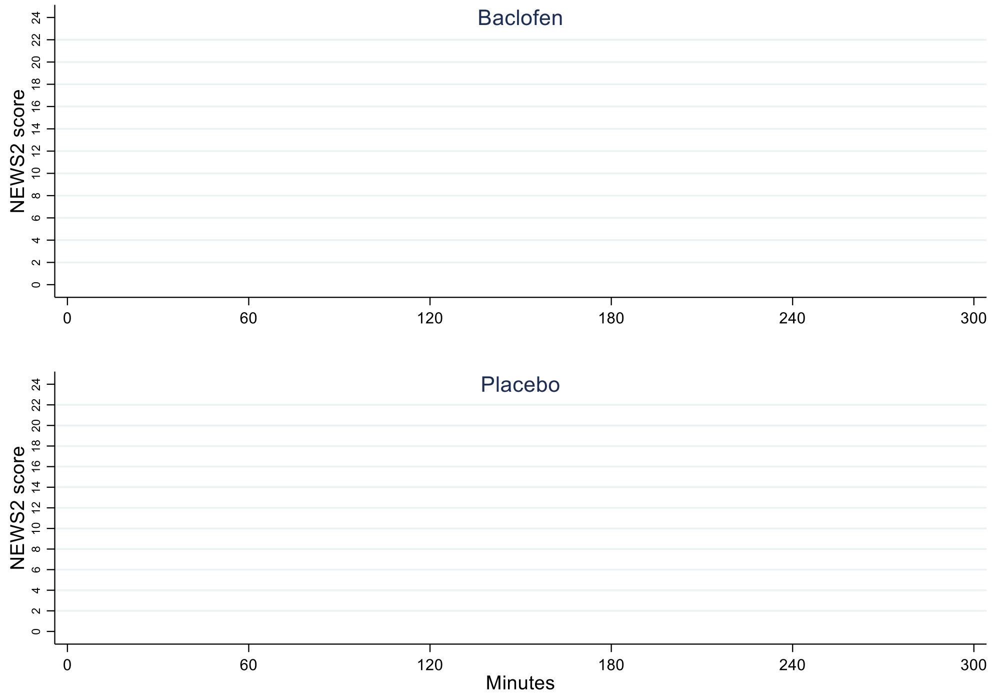

Figure A.4 Individual participant trajectories of NEWS2 scores over time by baclofen dose

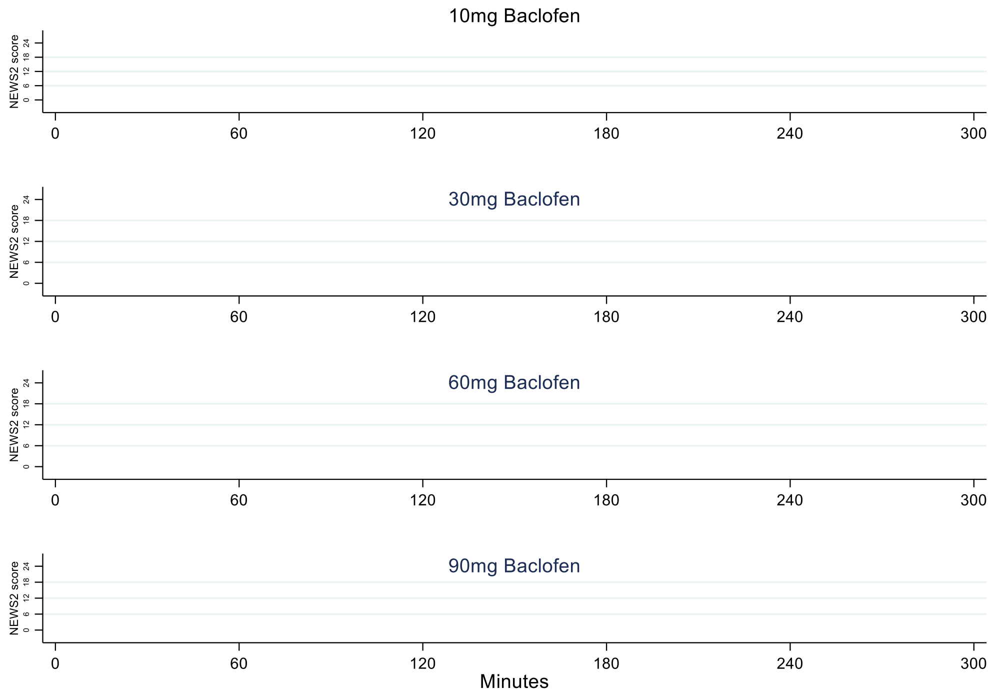

Figure A.5 Individual participant trajectories of NEWS2 scores over time by gender

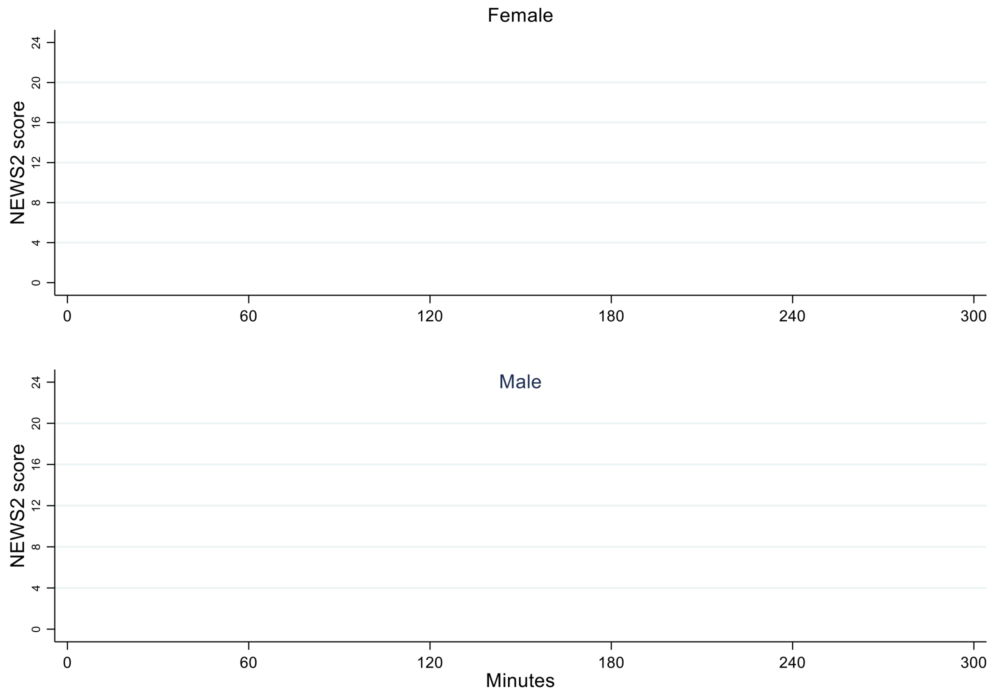

Figure A.6 Summaries of NEWS2 scores over time by treatment arm

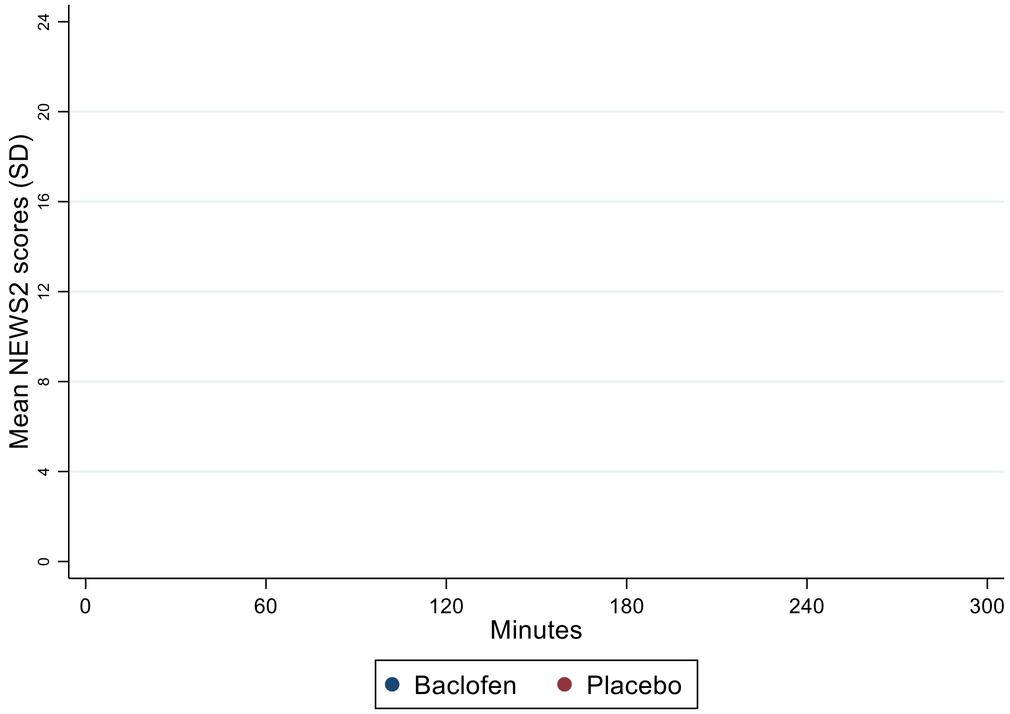

Figure A.7 Summaries of NEWS2 scores over time by baclofen dose

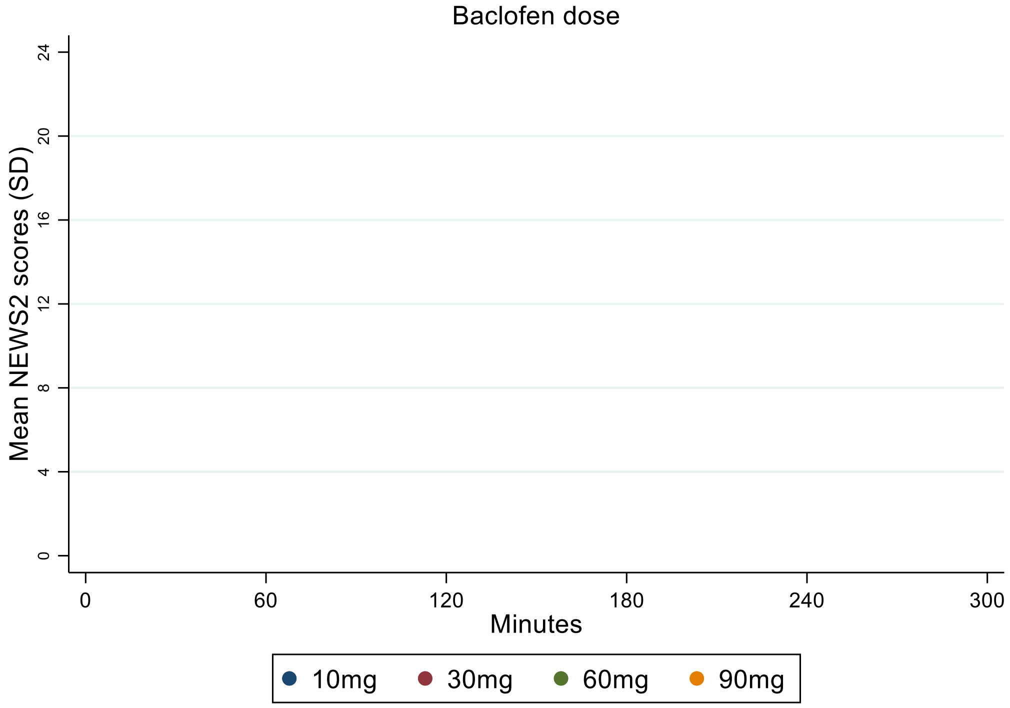

Figure A.8 Summaries of NEWS2 scores over time by gender

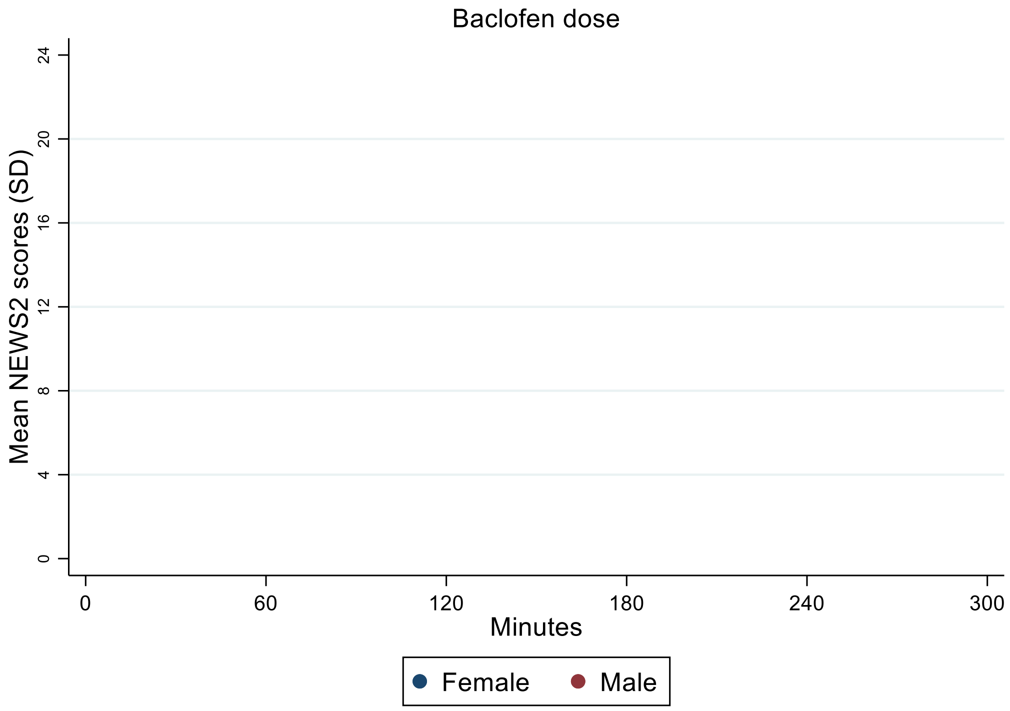

Figure A.9 Scatterplot of NEWS2 scores and methadone dose at 120 minutes

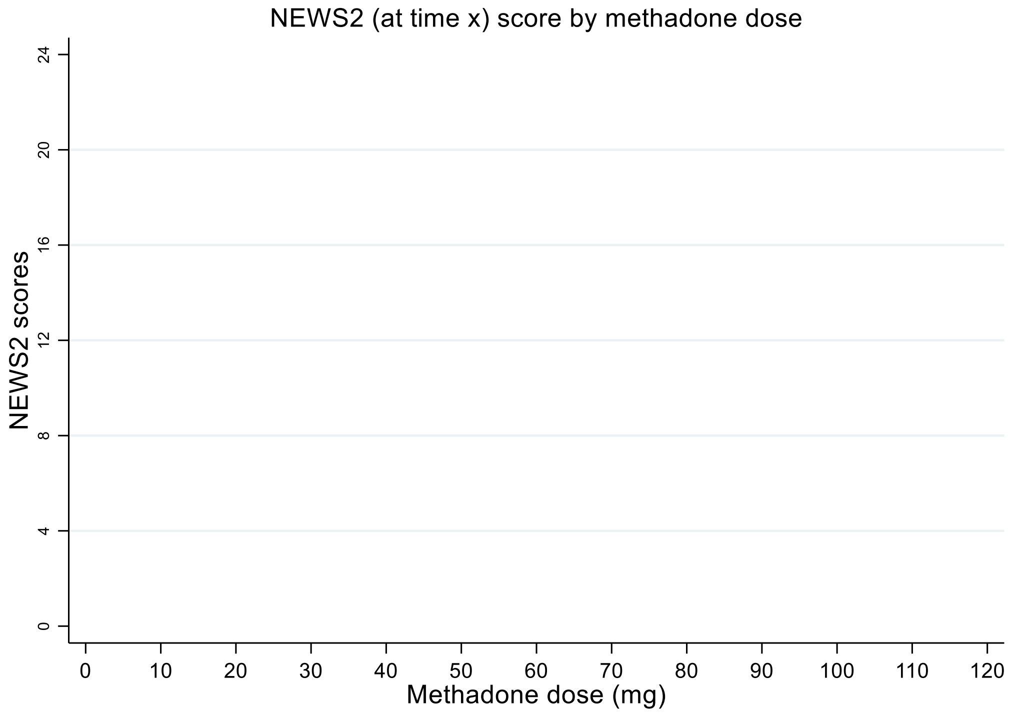

Table A.25: GCS score summaries by treatment arm

		Baclofen	Placebo	Total
Baseline				
	N (Nmissing)			
	Mean (SD)			
	Median (IQR)			
60 mins				
	N (Nmissing)			
	Mean (SD)			
	Median (IQR)			
120 mins				
	N (Nmissing)			
	Mean (SD)			
	Median (IQR)			
180 mins				
	N (Nmissing)			
	Mean (SD)			
	Median (IQR)			
240 mins				
	N (Nmissing)			
	Mean (SD)			
	Median (IQR)			
300 mins				
	N (Nmissing)			
	Mean (SD)			
	Median (IQR)			

Table A.26: Treatment effect estimates for GCS scores at each time-point from the linear mixed effects model (model 1)

		**Baclofen versus placebo N=xxx**	
GCS		Adjusted mean difference*	95% CIs
	60 minutes	x.xx	x.xx, x.xx
	120 minutes	x.xx	x.xx, x.xx
	180 minutes	x.xx	x.xx, x.xx
	240 minutes	x.xx	x.xx, x.xx
	300 minutes	x.xx	x.xx, x.xx

*adjusted for baseline GCS scores, methadone dose and methadone dose by treatment group (baclofen vs placebo) interaction

Table A.27: GCS score summaries by baclofen dose

		Baclofen dose			
		**10mg**	**30mg**	**60mg**	**90mg**
Baseline					
	N (Nmissing)				
	Mean (SD)				
	Median (IQR)				
60 mins					
	N (Nmissing)				
	Mean (SD)				
	Median (IQR)				
120 mins					
	N (Nmissing)				
	Mean (SD)				
	Median (IQR)				
180 mins					
	N (Nmissing)				
	Mean (SD)				
	Median (IQR)				
240 mins					
	N (Nmissing)				
	Mean (SD)				
	Median (IQR)				
300 mins					
	N (Nmissing)				
	Mean (SD)				
	Median (IQR)				

Table A.28: Treatment effect estimates for GCS scores at each time-point from the linear mixed effects model (model 2)

		**Baclofen dose (mg)<br>N=xxx**	
GCS		Adjusted mean difference*	95% CIs
	60 minutes	x.xx	(x.xx, x.xx)
	120 minutes	x.xx	(x.xx, x.xx)
	180 minutes	x.xx	(x.xx, x.xx)
	240 minutes	x.xx	(x.xx, x.xx)
	300 minutes	x.xx	(x.xx, x.xx)

*adjusted for baseline GCS scores, methadone dose and methadone dose by baclofen dose (mg) interaction

Table A.29: GCS score summaries by gender

		Gender	
		**Male**	**Female**
Baseline			
	N (Nmissing)		
	Mean (SD)		
	Median (IQR)		
60 mins			
	N (Nmissing)		
	Mean (SD)		
	Median (IQR)		
120 mins			
	N (Nmissing)		
	Mean (SD)		
	Median (IQR)		
180 mins			
	N (Nmissing)		
	Mean (SD)		
	Median (IQR)		
240 mins			
	N (Nmissing)		
	Mean (SD)		
	Median (IQR)		
300 mins			
	N (Nmissing)		
	Mean (SD)		
	Median (IQR)		

Table A.30: Correlation between GCS scores and methadone dose by treatment arm

Table A.31: Estimates of methadone effect on GCS scores between treatment arms from a linear mixed effects model (model 1)

		**Baclofen versus placebo N=xxx**	
GCS		Adjusted mean difference*	95% CIs
		x.xx	x.xx, x.xx

*adjusted for baseline GCS scores

Figure A.10 Individual participant trajectories of GCS scores over time by treatment arm

See template figure A.3

Figure A.11 Individual participant trajectories of GCS scores over time by baclofen dose

See template figure A.4

Figure A.12 Individual participant trajectories of GCS scores over time by gender

See template figure A.5

Figure A.13 Summaries of GCS scores over time by treatment arm

See template figure A.6

Figure A.14 Summaries of GCS scores over time by baclofen dose

See template figure A.7

Figure A.15 Summaries of GCS scores over time by gender

See template figure A.8

Figure A.16 Scatterplot of GCS scores and methadone dose at 120 minutes

See template figure A.9

Table A.32: QTc summaries by treatment arm

		Baclofen	Placebo	Total
Baseline				
	N (Nmissing)			
	Mean (SD)			
	Median (IQR)			
120 mins				
	N (Nmissing)			
	Mean (SD)			
	Median (IQR)			
300 mins				
	N (Nmissing)			
	Mean (SD)			
	Median (IQR)			

Table A.33: Treatment effect estimates for QTc scores at each time-point from the linear mixed effects model (model 1)

		Baclofen versus placebo N=xxx	
QTc		Adjusted mean difference*	95% CIs
	120 minutes	x.xx	x.xx, x.xx
	300 minutes	x.xx	x.xx, x.xx

*adjusted for baseline QTc scores, methadone dose and methadone dose by treatment group (baclofen vs placebo) interaction

Table A.34: QTc summaries by baclofen dose

		Baclofen dose			
		**10mg**	**30mg**	**60mg**	**90mg**
Baseline					
	N (Nmissing)				
	Mean (SD)				
	Median (IQR)				
120 mins					
	N (Nmissing)				
	Mean (SD)				
	Median (IQR)				
300 mins					
	N (Nmissing)				
	Mean (SD)				
	Median (IQR)				

Table A.35: Treatment effect estimates for QTc scores at each time-point from the linear mixed effects model (model 2)

		**Baclofen dose (mg) N=xxx**	
QTc		Adjusted mean difference*	(95% CIs)
	120 minutes	x.xx	(x.xx, x.xx)
	300 minutes	x.xx	(x.xx, x.xx)

*adjusted for baseline QTc scores, methadone dose and methadone dose by baclofen dose (mg) interaction

Table A.36: QTc summaries by gender

		Gender	
		**Male**	**Female**
Baseline			
	N (Nmissing)		
	Mean (SD)		
	Median (IQR)		
120 mins			
	N (Nmissing)		
	Mean (SD)		
	Median (IQR)		
300 mins			
	N (Nmissing)		
	Mean (SD)		
	Median (IQR)		

Table A.37: Correlation between QTc scores and methadone dose by treatment arm

Table A.38: Estimates of methadone effect on QTc between treatment arms from a linear mixed effects model (model 1)

		**Baclofen versus placebo N=xxx**	
QTc		Adjusted mean difference*	95% CIs
		x.xx	x.xx, x.xx

*adjusted for baseline QTc scores

Figure A.17 Individual participant trajectories of QTc over time by treatment arm

See template figure A.3

Figure A.18 Individual participant trajectories of QTc over time by baclofen dose

See template figure A.4

Figure A.19 Individual participant trajectories of QTc over time by gender

See template figure A.5

Figure A.20 Summaries of QTc over time by treatment arm

See template figure A.6

Figure A.21 Summaries of QTc over time by baclofen dose

See template figure A.7

Figure A.22 Summaries of QTc over time by gender

See template figure A.8

Figure A.23 Scatterplot of GCS scores and methadone dose at 120 minutes

See template figure A.9

Table A.39: Oxygen saturation (SpO2) levels by treatment arm

		**Baclofen**	**Placebo**	**Total**
Baseline				
	N (Nmissing)			
	Mean (SD)			
	Median (IQR)			
15 mins				
	N (Nmissing)			
	Mean (SD)			
	Median (IQR)			
30 mins				
	N (Nmissing)			
	Mean (SD)			
	Median (IQR)			
60 mins				
	N (Nmissing)			
	Mean (SD)			
	Median (IQR)			
90 mins				
	N (Nmissing)			
	Mean (SD)			
	Median (IQR)			
120 mins				
	N (Nmissing)			
	Mean (SD)			
	Median (IQR)			
180 mins				
	N (Nmissing)			
	Mean (SD)			
	Median (IQR)			
240 mins				
	N (Nmissing)			
	Mean (SD)			
	Median (IQR)			
300 mins				

	N (Nmissing)			
	Mean (SD)			
	Median (IQR)			

Table A.40: Treatment effect estimates for oxygen saturation (SpO2) levels at each time-point from the linear mixed effects model (model 1)

		**Baclofen versus placebo N=xxx**	
Oxygen saturation (SpO2) levels		Adjusted mean difference*	95% CIs
	15 minutes	x.xx	x.xx, x.xx
	30 minutes	x.xx	x.xx, x.xx
	60 minutes	x.xx	x.xx, x.xx
	90 minutes	x.xx	x.xx, x.xx
	120 minutes	x.xx	x.xx, x.xx
	180 minutes	x.xx	x.xx, x.xx
	240 minutes	x.xx	x.xx, x.xx
	300 minutes	x.xx	x.xx, x.xx

*adjusted for baseline oxygen saturation levels, methadone dose and methadone dose by treatment group (baclofen vs placebo) interaction

Table A.41: Oxygen saturation (Sp02) levels by baclofen dose

		Baclofen dose			
		**10mg**	**30mg**	**60mg**	**90mg**
Baseline					
	N (Nmissing)				
	Mean (SD)				
	Median (IQR)				
15 mins					
	N (Nmissing)				
	Mean (SD)				
	Median (IQR)				
30 mins					
	N (Nmissing)				
	Mean (SD)				
	Median (IQR)				
60 mins					
	N (Nmissing)				
	Mean (SD)				
	Median (IQR)				
90 mins					
	N (Nmissing)				
	Mean (SD)				
	Median (IQR)				
120 mins					
	N (Nmissing)				
	Mean (SD)				
	Median (IQR)				
180 mins					
	N (Nmissing)				
	Mean (SD)				
	Median (IQR)				
240 mins					
	N (Nmissing)				
	Mean (SD)				
	Median (IQR)				

300 mins					
	N (Nmissing)				
	Mean (SD)				
	Median (IQR)				

Table A.42: Treatment effect estimates for oxygen saturation (Sp02) at each time-point from the linear mixed effects model (model 2)

		**Baclofen dose (mg) N=xxx**	
Oxygen saturation (SpO2) levels		Adjusted mean difference*	(95% CIs)
	15 minutes	x.xx	(x.xx, x.xx)
	30 minutes	x.xx	(x.xx, x.xx)
	60 minutes	x.xx	(x.xx, x.xx)
	90 minutes	x.xx	(x.xx, x.xx)
	120 minutes	x.xx	(x.xx, x.xx)
	180 minutes	x.xx	(x.xx, x.xx)
	240 minutes	x.xx	(x.xx, x.xx)
	300 minutes	x.xx	(x.xx, x.xx)

*adjusted for baseline oxygen saturation levels, methadone dose and methadone dose by baclofen dose (mg) interaction

Table A.43: Oxygen saturation (Sp02) levels by gender

		Gender	
		**Male**	**Female**
Baseline			
	N (Nmissing)		
	Mean (SD)		
	Median (IQR)		
15 mins			
	N (Nmissing)		
	Mean (SD)		
	Median (IQR)		
30 mins			
	N (Nmissing)		
	Mean (SD)		
	Median (IQR)		
60 mins			
	N (Nmissing)		
	Mean (SD)		
	Median (IQR)		
90 mins			
	N (Nmissing)		
	Mean (SD)		
	Median (IQR)		
120 mins			
	N (Nmissing)		
	Mean (SD)		
	Median (IQR)		
180 mins			
	N (Nmissing)		
	Mean (SD)		
	Median (IQR)		
240 mins			
	N (Nmissing)		
	Mean (SD)		
	Median (IQR)		

360 mins			
	N (Nmissing)		
	Mean (SD)		
	Median (IQR)		

Table A.44: Correlation between oxygen saturation levels and methadone dose by treatment arm

Table A.45: Estimates of methadone effect on oxygen saturation (SpO2) levels between treatment arms from a linear mixed effects model (model 1)

		**Baclofen versus placebo N=xxx**	
Oxygen saturation (SpO2)		Adjusted mean difference*	95% CIs
		x.xx	x.xx, x.xx

*adjusted for baseline oxygen saturation (SpO2) levels

Figure A.24 Individual patient trajectories of oxygen saturation (Sp02) levels over time by treatment arm

See template figure A.3

Figure A.25 Individual patient trajectories of oxygen saturation (Sp02) levels over time by baclofen dose

See template figure A.4

Figure A.26 Individual patient trajectories of oxygen saturation (Sp02) levels over time by gender

See template figure A.5

Figure A.27 Summaries of oxygen saturation (Sp02) levels over time by treatment arm

See template figure A.6

Figure A.28 Summaries of oxygen saturation (Sp02) levels over time by baclofen dose

See template figure A.7

Figure A.29 Summaries of oxygen saturation (Sp02) levels over time by gender

See template figure A.8

Figure A.30 Scatterplot of oxygen saturation (Sp02) levels and methadone dose at 120 minutes

See template figure A.9

Table A.46: $tcCO_2$ by treatment arm

		Baclofen	Placebo	Total
Baseline				
	N (Nmissing)			
	Mean (SD)			
	Median (IQR)			
15 mins				
	N (Nmissing)			
	Mean (SD)			
	Median (IQR)			
30 mins				
	N (Nmissing)			
	Mean (SD)			
	Median (IQR)			
60 mins				
	N (Nmissing)			
	Mean (SD)			
	Median (IQR)			
90 mins				
	N (Nmissing)			
	Mean (SD)			
	Median (IQR)			
120 mins				
	N (Nmissing)			
	Mean (SD)			
	Median (IQR)			
180 mins				
	N (Nmissing)			
	Mean (SD)			
	Median (IQR)			
240 mins				
	N (Nmissing)			
	Mean (SD)			
	Median (IQR)			
300 mins				

	N (Nmissing)			
	Mean (SD)			
	Median (IQR)			

Table A.47: Treatment effect estimates for $tcCO_2$ levels at each time-point from the linear mixed effects model (model 1)

		**Baclofen versus placebo N=xxx**	
$tcCO_2$ levels		Adjusted mean difference*	95% CIs
	15 minutes	x.xx	x.xx, x.xx
	30 minutes	x.xx	x.xx, x.xx
	60 minutes	x.xx	x.xx, x.xx
	90 minutes	x.xx	x.xx, x.xx
	120 minutes	x.xx	x.xx, x.xx
	180 minutes	x.xx	x.xx, x.xx
	240 minutes	x.xx	x.xx, x.xx
	300 minutes	x.xx	x.xx, x.xx

*adjusted for baseline $tcCO_2$ level, methadone dose and methadone dose by treatment group (baclofen vs placebo) interaction

Table A.48: $tcCO_2$ by baclofen dose

		Baclofen dose			
		10mg	30mg	60mg	90mg
Baseline					
	N (Nmissing)				
	Mean (SD)				
	Median (IQR)				
15 mins					
	N (Nmissing)				
	Mean (SD)				
	Median (IQR)				
30 mins					
	N (Nmissing)				
	Mean (SD)				
	Median (IQR)				
60 mins					
	N (Nmissing)				
	Mean (SD)				
	Median (IQR)				
90 mins					
	N (Nmissing)				
	Mean (SD)				
	Median (IQR)				
120 mins					
	N (Nmissing)				
	Mean (SD)				
	Median (IQR)				
180 mins					
	N (Nmissing)				
	Mean (SD)				
	Median (IQR)				
240 mins					
	N (Nmissing)				
	Mean (SD)				
	Median (IQR)				

300 mins					
	N (Nmissing)				
	Mean (SD)				
	Median (IQR)				

Table A.49: Treatment effect estimates for $tcCO_2$ levels at each time-point from the linear mixed effects model (model 2)

		**Baclofen dose (mg) N=xxx**	
$tcCO_2$ levels		Adjusted mean difference*	(95% CIs)
	15 minutes	x.xx	(x.xx, x.xx)
	30 minutes	x.xx	(x.xx, x.xx)
	60 minutes	x.xx	(x.xx, x.xx)
	90 minutes	x.xx	(x.xx, x.xx)
	120 minutes	x.xx	(x.xx, x.xx)
	180 minutes	x.xx	(x.xx, x.xx)
	240 minutes	x.xx	(x.xx, x.xx)
	300 minutes	x.xx	(x.xx, x.xx)

*adjusted for baseline $tcCO_2$ levels, methadone dose and methadone dose by baclofen dose (mg) interaction

Table A.50: $tcCO_2$ by gender

		Gender	
		Male	Female
Baseline			
	N (Nmissing)		
	Mean (SD)		
	Median (IQR)		
15 mins			
	N (Nmissing)		
	Mean (SD)		
	Median (IQR)		
30 mins			
	N (Nmissing)		
	Mean (SD)		
	Median (IQR)		
60 mins			
	N (Nmissing)		
	Mean (SD)		
	Median (IQR)		
90 mins			
	N (Nmissing)		
	Mean (SD)		
	Median (IQR)		
120 mins			
	N (Nmissing)		
	Mean (SD)		
	Median (IQR)		
180 mins			
	N (Nmissing)		
	Mean (SD)		
	Median (IQR)		
240 mins			
	N (Nmissing)		
	Mean (SD)		
	Median (IQR)		

300 mins			
	N (Nmissing)		
	Mean (SD)		
	Median (IQR)		

Table A.51: Correlation between $tcCO_2$ levels and methadone dose by treatment arm

Table A.52: Estimates of methadone effect on $tcCO_2$ levels between treatment arms from a linear mixed effects model (model 1)

		**Baclofen versus placebo N=xxx**	
$tcCO_2$		Adjusted mean difference*	95% CIs
		x.xx	x.xx, x.xx

*adjusted for baseline $tcCO_2$ levels

Figure A.31 Individual patient trajectories of $tcCO_2$ over time by treatment arm

See template figure A.3

Figure A.32 Individual patient trajectories of $tcCO_2$ over time by baclofen dose

See template figure A.4

Figure A.33 Individual patient trajectories of $tcCO_2$ over time by gender

See template figure A.5

Figure A.34 Summaries of $tcCO_2$ over time by treatment arm

See template figure A.6

Figure A.35 Summaries of $tcCO_2$ over time by baclofen dose

See template figure A.7

Figure A.36 Summaries of $tcCO_2$ over time by gender

See template figure A.8

Figure A.37 Scatterplot of $tcCO_2$ levels and methadone dose at 120 minutes

See template figure A.9

Table A.53: Respiratory rate by treatment arm

		Baclofen	Placebo	Total
Baseline				
	N (Nmissing)			
	Mean (SD)			
	Median (IQR)			
15 mins				
	N (Nmissing)			
	Mean (SD)			
	Median (IQR)			
30 mins				
	N (Nmissing)			
	Mean (SD)			
	Median (IQR)			
60 mins				
	N (Nmissing)			
	Mean (SD)			
	Median (IQR)			
90 mins				
	N (Nmissing)			
	Mean (SD)			
	Median (IQR)			
120 mins				
	N (Nmissing)			
	Mean (SD)			
	Median (IQR)			
180 mins				
	N (Nmissing)			
	Mean (SD)			
	Median (IQR)			
240 mins				
	N (Nmissing)			
	Mean (SD)			
	Median (IQR)			
300 mins				

	N (Nmissing)			
	Mean (SD)			
	Median (IQR)			

Table A.54: Treatment effect estimates for respiratory rate at each time-point from the linear mixed effects model (model 1)

		**Baclofen versus placebo N=xxx**	
Respiratory rate		Adjusted mean difference*	95% CIs
	15 minutes	x.xx	x.xx, x.xx
	30 minutes	x.xx	x.xx, x.xx
	60 minutes	x.xx	x.xx, x.xx
	90 minutes	x.xx	x.xx, x.xx
	120 minutes	x.xx	x.xx, x.xx
	180 minutes	x.xx	x.xx, x.xx
	240 minutes	x.xx	x.xx, x.xx
	300 minutes	x.xx	x.xx, x.xx

*adjusted for baseline respiratory rate, methadone dose and methadone dose by treatment group (baclofen vs placebo) interaction

Table A.55: Respiratory rate by baclofen dose

		Baclofen dose			
		10mg	30mg	60mg	90mg
Baseline					
	N (Nmissing)				
	Mean (SD)				
	Median (IQR)				
15 mins					
	N (Nmissing)				
	Mean (SD)				
	Median (IQR)				
30 mins					
	N (Nmissing)				
	Mean (SD)				
	Median (IQR)				
60 mins					
	N (Nmissing)				
	Mean (SD)				
	Median (IQR)				
90 mins					
	N (Nmissing)				
	Mean (SD)				
	Median (IQR)				
120 mins					
	N (Nmissing)				
	Mean (SD)				
	Median (IQR)				
180 mins					
	N (Nmissing)				
	Mean (SD)				
	Median (IQR)				
240 mins					
	N (Nmissing)				
	Mean (SD)				
	Median (IQR)				

300 mins					
	N (Nmissing)				
	Mean (SD)				
	Median (IQR)				

Table A.56: Treatment effect estimates for respiratory rate at each time-point from the linear mixed effects model (model 2)

		**Baclofen dose (mg) N=xxx**	
Respiratory rate		Adjusted mean difference*	(95% CIs)
	15 minutes	x.xx	(x.xx, x.xx)
	30 minutes	x.xx	(x.xx, x.xx)
	60 minutes	x.xx	(x.xx, x.xx)
	90 minutes	x.xx	(x.xx, x.xx)
	120 minutes	x.xx	(x.xx, x.xx)
	180 minutes	x.xx	(x.xx, x.xx)
	240 minutes	x.xx	(x.xx, x.xx)
	300 minutes	x.xx	(x.xx, x.xx)

*adjusted for baseline respiratory rate, methadone dose and methadone dose by baclofen dose (mg) interaction

Table A.57: Respiratory rate by gender

		Gender	
		**Male**	**Female**
Baseline			
	N (Nmissing)		
	Mean (SD)		
	Median (IQR)		
15 mins			
	N (Nmissing)		
	Mean (SD)		
	Median (IQR)		
30 mins			
	N (Nmissing)		
	Mean (SD)		
	Median (IQR)		
60 mins			
	N (Nmissing)		
	Mean (SD)		
	Median (IQR)		
90 mins			
	N (Nmissing)		
	Mean (SD)		
	Median (IQR)		
120 mins			
	N (Nmissing)		
	Mean (SD)		
	Median (IQR)		
180 mins			
	N (Nmissing)		
	Mean (SD)		
	Median (IQR)		
240 mins			
	N (Nmissing)		
	Mean (SD)		
	Median (IQR)		

300 mins			
	N (Nmissing)		
	Mean (SD)		
	Median (IQR)		

Table A.58: Correlation between respiratory rate and methadone dose by treatment arm

Table A.59: Estimates of methadone effect on respiratory rate between treatment arms from a linear mixed effects model (model 1)

		**Baclofen versus placebo N=xxx**	
Respiratory rate		Adjusted mean difference*	95% CIs
		x.xx	x.xx, x.xx

*adjusted for baseline respiratory rates, methadone dose and methadone dose by treatment group (baclofen vs placebo) interaction

Figure A.38 Individual patient trajectories of respiratory rates over time by treatment arm

See template figure A.3

Figure A.39 Individual patient trajectories of respiratory rates over time by baclofen dose

See template figure A.4

Figure A.40 Individual patient trajectories of respiratory rates over time by gender

See template figure A.5

Figure A.41 Summaries of respiratory rates over time by treatment arm

See template figure A.6

Figure A.42 Summaries of respiratory rates over time by baclofen dose

See template figure A.7

Figure A.43 Summaries of respiratory rates over time by gender

See template figure A.8

Figure A.44 Scatterplot of respiratory rates and methadone dose at 120 minutes

See template figure A.9

Table A.60: Measures of respiratory function by treatment arm

Characteristic		Baclofen (N=xxx)		Placebo (N=xxx)		Total (N=xxx)	
		No. of events	No. of participants	No. of events	No. of participants	No. of events	No. of participants
		Mean (SD)	n (%)	Mean (SD)	n (%)	Mean (SD)	n (%)
**SpO_2**							
	Nmissing						
	Reduction in SpO_2 (≤91% for more than 30 seconds or >5% reduction in SpO_2 for more than 30 seconds)						
**Respiratory rate**							
	Nmissing						
	Reduced respiratory rate (≤8/min)						
**Apnoea**							
	Nmissing						
	Absence of inspiratory airflow for >30s combined with a sustained fall in SpO_2						

Table A.61: Measures of respiratory function by baclofen dose

		Baclofen dose (N=xxx)							
**Characteristic**		**10mg (N=xxx)**		**30mg (N=xxx)**		**60mg (N=xxx)**		**90mg (N=xxx)**	
		No. of events	No. of participants	No. of events	No. of participants	No. of events	No. of participants	No. of events	No. of participants
		Mean (SD)	n (%)	Mean (SD)	n (%)	Mean (SD)	n (%)	Mean (SD)	n (%)
**SpO_2**									
	Nmissing								
	Reduction in SpO_2 (≤91% for more than 30 seconds or >5% reduction in SpO_2 for more than 30 seconds)								
**Respiratory rate**									
	Nmissing								
	Reduced respiratory rate (≤8/min)								
**Apnoea**									
	Nmissing								
	Absence of inspiratory airflow for >30s combined with a sustained fall in SpO_2								

Table A.62: Measures of respiratory function by gender

		Gender (N=xxx)			
**Characteristic, n (%)**		**Male (N=xxx)**		**Female (N=xxx)**	
		No. of events	No. of participants	No. of events	No. of participants
		Mean (SD)	n (%)	Mean (SD)	n (%)
**SpO_2**					
	Nmissing				
	Reduction in SpO_2 (≤91% for more than 30 seconds or >5% reduction in SpO_2 for more than 30 seconds)				
**Respiratory rate**					
	Nmissing				
	Reduced respiratory rate (≤8/min)				
**Apnoea**					
	Nmissing				
	Absence of inspiratory airflow for >30s combined with a sustained fall in SpO_2				

Table A.63: Respiratory measures indicating sub-threshold respiratory depression by treatment arm

Characteristic		Baclofen (N=xxx)		Placebo (N=xxx)		Total (N=xxx)	
		No. of events	No. of participants	No. of events	No. of participants	No. of events	No. of participants
		Mean (SD)	n (%)	Mean (SD)	n (%)	Mean (SD)	n (%)
**SpO_2**							
	Nmissing						
	<92% or of >5% reduction for more than 10 seconds						
**CO_2**							
	Nmissing						
	$tcCO_2$% exceeding a partial pressure CO_2 increase by 1kPa or a partial pressure CO_2 increase by 1kPa						
**Respiratory rate**							
	Nmissing						
	Absence of inspiratory airflow for more than 10 seconds or respiratory rate drops <9/min						

Table A.64: Respiratory measures indicating sub-threshold respiratory depression by baclofen dose

	Baclofen dose (N=xxx)							
**Characteristic**	**10mg (N=xxx)**		**30mg (N=xxx)**		**60mg (N=xxx)**		**90mg (N=xxx)**	
	No. of events	No. of participants	No. of events	No. of participants	No. of events	No. of participants	No. of events	No. of participants
	Mean (SD)	n (%)	Mean (SD)	n (%)	Mean (SD)	n (%)	Mean (SD)	n (%)
**SpO_2**								
Nmissing								
<92% or of >5% reduction for more than 10 seconds								
**CO_2**								
Nmissing								
$tcCO_2$% exceeding a partial pressure CO_2 increase by 1kPa or a partial pressure CO_2 increase by 1kPa								
**Respiratory rate**								
Nmissing								
Absence of inspiratory airflow for more than 10 seconds or respiratory rate drops <9/min								

Table A.65: Respiratory measures indicating sub-threshold respiratory depression by gender

		Gender (N=xxx)			
**Characteristic**		**Male (N=xxx)**		**Female (N=xxx)**	
		No. of events	No. of participants	No. of events	No. of participants
		Mean (SD)	n (%)	Mean (SD)	n (%)
**SpO_2**					
	Nmissing				
	<92% or of >5% reduction for more than 10 seconds				
**CO_2**					
	Nmissing				
	$tcCO_2$% exceeding a partial pressure CO_2 increase by 1kPa or a partial pressure CO_2 increase by 1kPa				
**Respiratory rate**					
	Nmissing				
	Absence of inspiratory airflow for more than 10 seconds or respiratory rate drops <9/min				

Table A.66: Total SHAS score by treatment arm

		Baclofen	Placebo	Total
Baseline				
	N (Nmissing)			
	Mean (SD)			
	Median (IQR)			
60 mins				
	N (Nmissing)			
	Mean (SD)			
	Median (IQR)			
120 mins				
	N (Nmissing)			
	Mean (SD)			
	Median (IQR)			
180 mins				
	N (Nmissing)			
	Mean (SD)			
	Median (IQR)			
240 mins				
	N (Nmissing)			
	Mean (SD)			
	Median (IQR)			
300 mins				
	N (Nmissing)			
	Mean (SD)			
	Median (IQR)			

Table A.67: Treatment effect estimates for total SHAS scores at each time-point from the linear mixed effects model (model 1)

		**Baclofen versus placebo N=xxx**	
Total SHAS score		Adjusted mean difference*	95% CIs
	60 minutes	x.xx	x.xx, x.xx
	120 minutes	x.xx	x.xx, x.xx
	180 minutes	x.xx	x.xx, x.xx
	240 minutes	x.xx	x.xx, x.xx
	300 minutes	x.xx	x.xx, x.xx

*adjusted for baseline total SHAS score, methadone dose and methadone dose by treatment group (baclofen vs placebo) interaction

Table A.68: Total SHAS by baclofen dose

		Baclofen dose			
		**10mg**	**30mg**	**60mg**	**90mg**
Baseline					
	N (Nmissing)				
	Mean (SD)				
	Median (IQR)				
60 mins					
	N (Nmissing)				
	Mean (SD)				
	Median (IQR)				
120 mins					
	N (Nmissing)				
	Mean (SD)				
	Median (IQR)				
180 mins					
	N (Nmissing)				
	Mean (SD)				
	Median (IQR)				
240 mins					
	N (Nmissing)				
	Mean (SD)				
	Median (IQR)				
300 mins					
	N (Nmissing)				
	Mean (SD)				
	Median (IQR)				

Table A.69: Treatment effect estimates for total SHAS score at each time-point from the linear mixed effects model (model 2)

		**Baclofen dose (mg)<br>N=xxx**	
Total SHAS score		Adjusted mean difference*	(95% CIs)
	60 minutes	x.xx	(x.xx, x.xx)
	120 minutes	x.xx	(x.xx, x.xx)
	180 minutes	x.xx	(x.xx, x.xx)
	240 minutes	x.xx	(x.xx, x.xx)
	300 minutes	x.xx	(x.xx, x.xx)

*adjusted for baseline total SHAS score, methadone dose and methadone dose by baclofen dose (mg) interaction

Table A.70: Total SHAS score by gender

		Gender	
		**Male**	**Female**
Baseline			
	N (Nmissing)		
	Mean (SD)		
	Median (IQR)		
60 mins			
	N (Nmissing)		
	Mean (SD)		
	Median (IQR)		
120 mins			
	N (Nmissing)		
	Mean (SD)		
	Median (IQR)		
180 mins			
	N (Nmissing)		
	Mean (SD)		
	Median (IQR)		
240 mins			
	N (Nmissing)		
	Mean (SD)		
	Median (IQR)		
300 mins			
	N (Nmissing)		
	Mean (SD)		
	Median (IQR)		

Table A.71: Correlation between total SHAS score and methadone dose by treatment arm

Table A.72: Estimates of methadone effect on total SHAS score between treatment arms from a linear mixed effects model (model 1)

		**Baclofen versus placebo N=xxx**	
Total SHAS score		Adjusted mean difference*	95% CIs
		x.xx	x.xx, x.xx

*adjusted for baseline total SHAS score

Figure A.45 Individual patient trajectories of total SHAS scores over time by treatment arm

See template figure A.3

Figure A.46 Individual patient trajectories of total SHAS scores over time by baclofen dose

See template figure A.4

Figure A.47 Individual patient trajectories of total SHAS scores over time by gender

See template figure A.5

Figure A.48 Summaries of total SHAS scores over time by treatment arm

See template figure A.6

Figure A.49 Summaries of total SHAS scores over time by baclofen dose

See template figure A.7

Figure A.50 Summaries of total SHAS scores over time by gender

See template figure A.8

Figure A.51 Scatterplot of total SHAS score and methadone dose at 120 minutes

See template figure A.9

Table A.73: DEQ liking score by treatment arm

		Baclofen	Placebo	Total
Baseline				
	N (Nmissing)			
	Mean (SD)			
	Median (IQR)			
60 mins				
	N (Nmissing)			
	Mean (SD)			
	Median (IQR)			
120 mins				
	N (Nmissing)			
	Mean (SD)			
	Median (IQR)			
180 mins				
	N (Nmissing)			
	Mean (SD)			
	Median (IQR)			
240 mins				
	N (Nmissing)			
	Mean (SD)			
	Median (IQR)			
300 mins				
	N (Nmissing)			
	Mean (SD)			
	Median (IQR)			

Table A.74: DEQ **wanting more** score by treatment arm

		Baclofen	Placebo	Total
Baseline				
	N (Nmissing)			
	Mean (SD)			
	Median (IQR)			
60 mins				
	N (Nmissing)			
	Mean (SD)			
	Median (IQR)			
120 mins				
	N (Nmissing)			
	Mean (SD)			
	Median (IQR)			
180 mins				
	N (Nmissing)			
	Mean (SD)			
	Median (IQR)			
240 mins				
	N (Nmissing)			
	Mean (SD)			
	Median (IQR)			
300 mins				
	N (Nmissing)			
	Mean (SD)			
	Median (IQR)			

Table A.75: Treatment effect estimates for DEQ **liking** score at each time-point from the linear mixed effects model (model 1)

		**Baclofen versus placebo N=xxx**	
DEQ liking		Adjusted mean difference*	95% CIs
	60 minutes	x.xx	x.xx, x.xx
	120 minutes	x.xx	x.xx, x.xx
	180 minutes	x.xx	x.xx, x.xx
	240 minutes	x.xx	x.xx, x.xx
	300 minutes	x.xx	x.xx, x.xx

*adjusted for baseline DEQ liking score, methadone dose and methadone dose by treatment group (baclofen vs placebo) interaction

Table A.76: Treatment effect estimates for DEQ **wanting more** score at each time-point from the linear mixed effects model (model 1)

		**Baclofen versus placebo N=xxx**	
DEQ wanting more		Adjusted mean difference*	95% CIs
	60 minutes	x.xx	x.xx, x.xx
	120 minutes	x.xx	x.xx, x.xx
	180 minutes	x.xx	x.xx, x.xx
	240 minutes	x.xx	x.xx, x.xx
	300 minutes	x.xx	x.xx, x.xx

*adjusted for baseline DEQ wanting more score, methadone dose and methadone dose by treatment group (baclofen vs placebo) interaction

Table A.77: DEQ **liking** by baclofen dose

		Baclofen dose			
		10mg	30mg	60mg	90mg
Baseline					
	N (Nmissing)				
	Mean (SD)				
	Median (IQR)				
60 mins					
	N (Nmissing)				
	Mean (SD)				
	Median (IQR)				
120 mins					
	N (Nmissing)				
	Mean (SD)				
	Median (IQR)				
180 mins					
	N (Nmissing)				
	Mean (SD)				
	Median (IQR)				
240 mins					
	N (Nmissing)				
	Mean (SD)				
	Median (IQR)				
300 mins					
	N (Nmissing)				
	Mean (SD)				
	Median (IQR)				

Table A.78: DEQ **wanting more** by baclofen dose

		Baclofen dose			
		**10mg**	**30mg**	**60mg**	**90mg**
Baseline					
	N (Nmissing)				
	Mean (SD)				
	Median (IQR)				
60 mins					
	N (Nmissing)				
	Mean (SD)				
	Median (IQR)				
120 mins					
	N (Nmissing)				
	Mean (SD)				
	Median (IQR)				
180 mins					
	N (Nmissing)				
	Mean (SD)				
	Median (IQR)				
240 mins					
	N (Nmissing)				
	Mean (SD)				
	Median (IQR)				
300 mins					
	N (Nmissing)				
	Mean (SD)				
	Median (IQR)				

Table A.79: Treatment effect estimates for DEQ **liking** at each time-point from the linear mixed effects model (model 2)

		**Baclofen dose (mg) N=xxx**	
DEQ liking		Adjusted mean difference*	(95% CIs)
	60 minutes	x.xx	(x.xx, x.xx)
	120 minutes	x.xx	(x.xx, x.xx)
	180 minutes	x.xx	(x.xx, x.xx)
	240 minutes	x.xx	(x.xx, x.xx)
	300 minutes	x.xx	(x.xx, x.xx)

*adjusted for baseline DEQ liking score, methadone dose and methadone dose by baclofen dose (mg) interaction

Table A.80: Treatment effect estimates for DEQ **wanting more** at each time-point from the linear mixed effects model (model 2)

		**Baclofen dose (mg) N=xxx**	
DEQ wanting more		Adjusted mean difference*	(95% CIs)
	60 minutes	x.xx	(x.xx, x.xx)
	120 minutes	x.xx	(x.xx, x.xx)
	180 minutes	x.xx	(x.xx, x.xx)
	240 minutes	x.xx	(x.xx, x.xx)
	300 minutes	x.xx	(x.xx, x.xx)

*adjusted for baseline DEQ wanting more score, methadone dose and methadone dose by baclofen dose (mg) interaction

Table A.81: DEQ **liking** score by gender

		Gender	
		Male	Female
Baseline			
	N (Nmissing)		
	Mean (SD)		
	Median (IQR)		
60 mins			
	N (Nmissing)		
	Mean (SD)		
	Median (IQR)		
120 mins			
	N (Nmissing)		
	Mean (SD)		
	Median (IQR)		
180 mins			
	N (Nmissing)		
	Mean (SD)		
	Median (IQR)		
240 mins			
	N (Nmissing)		
	Mean (SD)		
	Median (IQR)		
300 mins			
	N (Nmissing)		
	Mean (SD)		
	Median (IQR)		

Table A.82: DEQ **wanting more** score by gender

		Gender	
		**Male**	**Female**
Baseline			
	N (Nmissing)		
	Mean (SD)		
	Median (IQR)		
60 mins			
	N (Nmissing)		
	Mean (SD)		
	Median (IQR)		
120 mins			
	N (Nmissing)		
	Mean (SD)		
	Median (IQR)		
180 mins			
	N (Nmissing)		
	Mean (SD)		
	Median (IQR)		
240 mins			
	N (Nmissing)		
	Mean (SD)		
	Median (IQR)		
300 mins			
	N (Nmissing)		
	Mean (SD)		
	Median (IQR)		

Table A.83: Correlation between DEQ **liking** and methadone dose by treatment arm

Table A.84: Correlation between DEQ **wanting more** and methadone dose by treatment arm

Table A.85: Estimates of methadone effect on DEQ **liking** between treatment arms from a linear mixed effects model (model 1)

		Baclofen versus placebo N=xxx	
DEQ liking		Adjusted mean difference*	95% CIs
		x.xx	x.xx, x.xx

*adjusted for baseline DEQ liking score

Table A.86: Estimates of methadone effect on DEQ **wanting more** between treatment arms from a linear mixed effects model (model 1)

		Baclofen versus placebo N=xxx	
DEQ wanting more		Adjusted mean difference*	95% CIs
		x.xx	x.xx, x.xx

*adjusted for baseline DEQ wanting more score

Figure A.52 Individual patient trajectories of DEQ **liking scores** over time by treatment arm

See template figure A.3

Figure A.53 Individual patient trajectories of DEQ **wanting more** scores over time by treatment arm

See template figure A.3

Figure A.54 Individual patient trajectories of DEQ **liking scores** over time by baclofen dose

See template figure A.4

Figure A.55 Individual patient trajectories of DEQ **wanting scores** over time by baclofen dose

See template figure A.4

Figure A.56 Individual patient trajectories of DEQ **liking scores** over time by gender

See template figure A.5

Figure A.57 Individual patient trajectories of DEQ **wanting scores** over time by gender

See template figure A.5

Figure A.58 Summaries of DEQ **liking scores** over time by treatment arm

See template figure A.6

Figure A.59 Summaries of DEQ **wanting more** scores over time by treatment arm

See template figure A.6

Figure A.60 Summaries of DEQ **liking** scores over time by baclofen dose

See template figure A.7

Figure A.61 Summaries of DEQ **wanting more** scores over time by baclofen dose

See template figure A.7

Figure A.62 Summaries of DEQ **liking** over time by gender

See template figure A.8

Figure A.63 Summaries of DEQ **wanting more** scores over time by gender

See template figure A.8

Figure A.64 Scatterplot of DEQ **liking** and methadone dose at 120 minutes

See template figure A.9

Figure A.65 Scatterplot of DEQ **wanting more** and methadone dose at 120 minutes

See template figure A.9

Table A.87: Summary of adverse event type by treatment arm

	**Baclofen**		**Placebo**		**Total**	
Event type	Number of events	Number of participants with events	Number of events	Number of participants with events	Number of events	Number of participants with events
	n	n (%)	n	n (%)	n	n (%)
All events						
AE						
AR						
UAR						
SAE						
SAR						
USAR						

Acronyms: AE – adverse event; AR – adverse reaction; UAR – unexpected adverse reaction; SAE – serious adverse event; SAR – serious adverse reaction; USAR – unexpected serious adverse reaction

Table A.88: Adverse events by preferred term and treatment arm

		Baclofen		Placebo		Total	
Preferred terms		Number of events	Number of participants with events	Number of events	Number of participants with events	Number of events	Number of participants with events
		n	n (%)	n	n (%)	n	n (%)
	Preferred term 1						
	Preferred term 2						
	Preferred term 1						
	Preferred term 2						
…..							

Table A.89: Adverse events by preferred term, severity grading and treatment arm

	**Baclofen**						**Placebo**					
	Mild		Moderate		Severe		Mild		Moderate		Severe	
Preferred terms	N events	N participants	N events	N participants	N events	N participants	N events	N participants	N events	N participants	N events	N participants
	n	n (%)	n	n (%)	n	n (%)	n	n (%)	n	n (%)	n	n (%)
Preferred term 1												
Preferred term 2												
Preferred term 1												
Preferred term 2												
…..												

Figure A.66: Stacked bar chart of adverse event severity

Table A.90: Adverse events by preferred term and treatment arm

		Baclofen		Placebo					
Preferred terms		Number of events	Number of participants with events	Number of events	Number of participants with events	Risk difference	95% CI	Incident rate ratio	95% CI
		n	n (%)	n	n (%)				
	Preferred term 1								
	Preferred term 2								
	Preferred term 1								
	Preferred term 2								
…..									

Figure A.67: Dot plot of adverse events

Table A.91: Serious adverse event listings for individual participants

Participant ID	Event	Arm	Onset date	Resolved date	Time since randomisation (days)	Severity	Relatedness (investigator)	Expected (investigator)	Action taken	Outcome
1										
1										

References

AGABIO, R., SINCLAIR, J. M., ADDOLORATO, G., AUBIN, H. J., BERAHA, E. M., CAPUTO, F., CHICK, J. D., DE LA SELLE, P., FRANCHITTO, N., GARBUTT, J. C., HABER, P. S., HEYDTMANN, M., JAURY, P., LINGFORD-HUGHES, A. R., MORLEY, K. C., MÜLLER, C. A., OWENS, L., PASTOR, A., PATERSON, L. M., PÉLISSIER, F., ROLLAND, B., STAFFORD, A., THOMPSON, A., VAN DEN BRINK, W., DE BEAUREPAIRE, R. & LEGGIO, L. 2018. Baclofen for the treatment of alcohol use disorder: the Cagliari Statement. *Lancet Psychiatry,* 5**,** 957-960.

JOLLEY, C. J., BELL, J., RAFFERTY, G. F., MOXHAM, J. & STRANG, J. 2015. Understanding Heroin Overdose: A Study of the Acute Respiratory Depressant Effects of Injected Pharmaceutical Heroin. *PLoS One,* 10**,** e0140995.

MOZGUNOV, P., CRO, S., LINGFORD-HUGHES, A., PATERSON, L. M. & JAKI, T. 2021. A dose-finding design for dual-agent trials with patient-specific doses for one agent with application to an opiate detoxification trial. *Pharmaceutical Statistics,* (in press).

NEUENSCHWANDER B, M. A., TANG Z, ROYCHOUDHURY S, WANDEL S, BAILEY S. 2015. A Bayesian industry approach to phase I combination trials in oncology. *Statistical methods in drug combination studies***,** 95–135.

ROLLAND, B., SIMON, N., FRANCHITTO, N. & AUBIN, H. J. 2020. France Grants an Approval to Baclofen for Alcohol Dependence. *Alcohol Alcohol,* 55**,** 44-45.

SCHULZ, K. F., ALTMAN, D. G., MOHER, D. & GROUP, C. 2010. Consort 2010 statement: Updated guidelines for reporting parallel group randomized trials. *Annals of Internal Medicine,* 152**,** 726-732.
