# Supplementary material for: FORWARDS-1: an adaptive, single-blind, placebo-controlled ascending dose study of acute baclofen on safety parameters in opioid dependence during methadone-maintenance treatment—a pharmacokinetic-pharmacodynamic study
Source: Trials. 2022 Oct 18;23:880. doi: 10.1186/s13063-022-06821-9 (PMC9579625; doi:10.1186/s13063-022-06821-9)
Supplement: Supplementary file 2 — Additional file 2. Exploratory objectives, hypotheses, outcomes and planned analyses. [file 13063_2022_6821_MOESM2_ESM.docx]

Additional Files 2

**Exploratory objectives, hypotheses, outcomes and planned analyses.**

**Exploratory objectives:**

Exploratory objectives will include determination of whether there is evidence of reduced sensitivity to baclofen through determination of objective and subjective pharmacokinetic-pharmacodynamic (PK-PD) responses using the following measures: [i] plasma growth hormone levels, [ii] plasma baclofen levels, [iii] plasma methadone levels. These measures, along with subjective drug response, will be compared with those of historic controls and those with alcohol dependence [41].

We will investigate the variability in PK-PD responses [i] to baclofen at different baclofen dose levels, [ii] to baclofen at different methadone levels, [iii] tolerability aspects, demographic factors (e.g. age) and to identify possible markers of efficacy of baclofen relative to placebo (e.g. sleep, anxiety, restless legs).

**Exploratory objective hypotheses:**

We hypothesise that opiate dependent participants will demonstrate a comparable pharmacokinetic profile in response to baclofen as compared with healthy controls and those with alcohol dependence (data from [41]).

We hypothesise that baclofen will reduce anxiety after acute dosing and improve sleep measures during the subsequent night of sleep, compared with placebo.

We hypothesise that we will observe signs of disturbed sleep and respiratory dysfunction e.g. sleep apnoea, sleep disordered breathing (SDB) in our opiate dependent cohort.

We hypothesise that opiate dependent individuals will demonstrate lower sensitivity to baclofen as compared with historic controls with reduced: a) growth hormone response relative to controls at peak effect (~2h post dose) following 60mg baclofen, b) sedation response (self-report measures) relative to controls at peak effect (2-3h post dose) following 60mg baclofen.

**Exploratory outcomes:**

**Sedation measures:**

- Mean Total-SHAS score at peak PD response (2-3h) at 60mg baclofen dose level, relative to historical controls
- Time-course of T-SHAS at each baclofen dose level, relative to placebo

**Plasma levels: PK and growth hormone measures**:

- Plasma baclofen concentrations
  - Mean peak baclofen plasma concentration (2-4h) at each baclofen dose level, relative to placebo
  - Time-course of plasma baclofen levels
  - Cmax; maximum (peak) plasma concentration
  - Tmax (time to reach Cmax)
  - t1/2 (elimination half-life)
  - Variability in PK parameters by gender
- Plasma methadone concentrations
  - Mean plasma concentration (2-4h) at each baclofen dose level, relative to placebo
  - Time-course of plasma methadone levels
- Plasma growth hormone (GH) concentrations, a surrogate marker of GABA-B receptor function
  - Mean plasma concentration (2-4h) at each baclofen dose level, relative to placebo
  - Mean peak plasma concentration (2h) at the 60mg baclofen dose level, relative to controls
  - Time-course of plasma GH levels
  - Variability in GH profile by gender

**Symptom & Other measures:**

- Visual analogue scales for anxiety, craving at each baclofen dose level, relative to placebo and associated time-course.
- Heart rate, blood pressure, body temperature at each baclofen dose level, relative to placebo and associated time-course.

**Sleep measures:**

- LSEQ at each baclofen dose level relative to placebo
  - Improvement in LSEQ score for ‘getting to sleep’ and ‘quality of sleep’ factors, no change in ‘awakening following sleep’ or behaviour following wake’ factors
- Actigraphy
  - 1 to 2 week rest-activity profile to establish sleep norms
  - Nocturnal activity following baclofen relative to sleep norm
- Overnight oximetry
  - Sleep outcomes (e.g. sleep time, efficiency, sleep cycles, sleep stages, hypnogram)
  - Estimate of severity of sleep disordered breathing (American Academy of Sleep Medicine Criteria); mild, moderate, severe
  - Apnoea-Hypopnoea index (AHI, overall score), SpO2 (%), heart rate (bpm), heart rate variability; continuous outputs will be collected (minute resolution) and averages calculated (mean/h)
  - Parameters will be compared across baclofen dose levels, between baclofen vs placebo sessions, and correlated with methadone dose, STOP-BANG score (validated SDB screening questionnaire with high sensitivity), other risk factors for SDB and objective and subjective measures of sleep quality

**Phenotypic measures**:

- demographic (gender, age), clinical (methadone dose), drug & alcohol history, validated questionnaire measures.
  1. These measures will be related to the primary and secondary outcomes to support the validity of the adaptive trial design, estimate variability in the signal across these demographic features, to establish novel relationships or conversely, to control for outlier effects.

**Analysis**

Exploratory analysis of sedation, PK/PD endpoints (plasma baclofen, methadone and growth hormone) to determine GABA-B sensitivity, and symptom measures will be analysed as previously described (Durant et al, 2018).

Actigraphy data will be collected and downloaded using Open Movement software (OMGui, Newcastle University, UK), and analysed using open source R package GGIR, available online: with activity data processed in 60-s epochs, and coded as sleep, sedentary behaviour, light, moderate and vigorous physical activity

Anonymised data outcomes derived from the overnight finger-worn oximetry device are calculated automatically based on movement and SpO2 via app software algorithms (SleepOn®) and will be tabulated and compared with published norms and/or matched control data, as appropriate.
